# Tissue-Specific Transcriptomic Profiling of *Sorghum propinquum* using a Rice Genome Array

**DOI:** 10.1371/journal.pone.0060202

**Published:** 2013-03-25

**Authors:** Ting Zhang, Xiuqin Zhao, Liyu Huang, Xiaoyue Liu, Ying Zong, Linghua Zhu, Daichang Yang, Binying Fu

**Affiliations:** 1 Institute of Crop Sciences/National Key Facility for Crop Gene Resources and Genetic Improvement, Chinese Academy of Agricultural Sciences, Beijing, China; 2 State Key Laboratory of Hybrid Rice, College of Life Sciences, Wuhan University, Wuhan, China; Kansas State University, United States of America

## Abstract

Sorghum (*Sorghum bicolor*) is one of the world's most important cereal crops. *S. propinquum* is a perennial wild relative of *S. bicolor* with well-developed rhizomes. Functional genomics analysis of *S. propinquum*, especially with respect to molecular mechanisms related to rhizome growth and development, can contribute to the development of more sustainable grain, forage, and bioenergy cropping systems. In this study, we used a whole rice genome oligonucleotide microarray to obtain tissue-specific gene expression profiles of *S. propinquum* with special emphasis on rhizome development. A total of 548 tissue-enriched genes were detected, including 31 and 114 unique genes that were expressed predominantly in the rhizome tips (RT) and internodes (RI), respectively. Further GO analysis indicated that the functions of these tissue-enriched genes corresponded to their characteristic biological processes. A few distinct *cis*-elements, including ABA-responsive RY repeat CATGCA, sugar-repressive TTATCC, and GA-responsive TAACAA, were found to be prevalent in RT-enriched genes, implying an important role in rhizome growth and development. Comprehensive comparative analysis of these rhizome-enriched genes and rhizome-specific genes previously identified in *Oryza longistaminata* and *S. propinquum* indicated that phytohormones, including ABA, GA, and SA, are key regulators of gene expression during rhizome development. Co-localization of rhizome-enriched genes with rhizome-related QTLs in rice and sorghum generated functional candidates for future cloning of genes associated with rhizome growth and development.

## Introduction

Sorghum [*Sorghum bicolor* (L.) Moench], which is widely grown throughout arid and semi-arid tropical regions, is the world's fifth most important cereal crop [1]. Because it is more resistant to drought, extreme temperature, and nutrient deficiency than maize, soybeans, wheat, and other crops, phenomena such as C4 photosynthesis, drought tolerance, signaling compound response, and aphid and high salinity resistance have been extensively investigated in sorghum [2], [3], [4], [5], [6], [7]. In addition, the use of sorghum in biofuel production promises to further increase the economic impact of this species.

*Sorghum bicolor* is the most economically important of the approximately 30 species in the genus. *Sorghum bicolor* is cultivated for grain and forage, while a wild relative native from Asia [8], *S. propinquum* (Kunth) Hitchc., is cultivated only for forage. *Sorghum propinquum* is a perennial with small seeds, high levels of tillering, narrow leaves, and well-developed rhizomes [9]. Rhizomes, which are underground stems, are associated with both perenniality and biomass partitioning; in Sorghum, their growth and development is controlled by multiple genes, as revealed by genetic analysis using a *S. bicolor* × *S. propinquum* mapping population [10].

Further elucidation of the genetic control of rhizome growth and development may contribute to the development of more sustainable grain, forage, and bioenergy cropping systems. A few efforts have been made to characterize the regulatory mechanisms of rhizome development. For example, using cDNA macroarray analysis, a number of genes rhizome-enriched in *S. propinquum* were implicated in secondary and hormone metabolism, abiotic stimuli, and development [11]. In another study, comparative analysis of coding regions and regulatory sequences for 54 rhizome-enriched genes in *S. propinquum* and *S. bicolor* indicated that several important *cis*-elements were more abundant in *S. propinquum* promoters than in those of non-rhizomatous *S. bicolor* or *Oryza sativa* [12]. Using *O. longistaminata* and *Phyllostachys praecox,* many efforts have been made to elucidate the genes and molecular mechanisms underlying the rhizomatous trait [13], [14], [15], [16], [17], [18], [19], [20]. Hu et al. [16] reported that the rhizome phenotype in *O. longistaminata* is controlled by two dominant-complementary genes, *Rhz2* and *Rhz3*, and comparative mapping studies indicated that each gene closely corresponds to a major quantitative trait locus (QTL) controlling rhizomatousness in *S. propinquum*. In addition, a set of rhizome-specific genes were identified by genome-wide differential expression analysis in *O. longistaminata*, suggesting a complex gene regulatory network underlying rhizome development and growth [18]. These results collectively provide a foundation for cloning genes governing rhizome-related traits.

Because of its high-throughput capability, the microarray platform has been widely used for transcriptome analysis. Previous studies have demonstrated that heterologous microarrays can be used to efficiently profile gene expressions when species-specific microarrays are not available [21], [22], [23], [24], [25], [26], [27]. For such cross-species gene expression studies, there is evidence suggesting that a long oligonucleotide-based (cDNA or 60-mer) microarray platform may be more suitable than a short oligonucleotide-based (25-mer) one [28]. Although the sorghum genome has been published [29], few transcriptome data are available for this species.

It has been estimated that rice diverged from the common ancestor of sorghum and maize approximately 50 million years ago [30], [31]. Sorghum-rice alignments based on the completely-sequenced *S. bicolor* and *O. sativa* genomes demonstrate high levels of DNA conservation between the two species. In addition, the number and sizes of sorghum gene families are similar to those of Arabidopsis and rice. It has been observed that 39.9% of rice-sorghum aligned sequences are conserved at the 70%/100 bp level, and 77.5% of the length of sorghum exon sequences overlap with those of rice [29], [32], [33]. Because sorghum and rice are members of the same plant family and, based on sequence similarity, are closely related to each other, we chose to hybridize sorghum RNA to a rice microarray. *Oryza sativa* (unlike *O. longistaminata*) is not rhizomatous, but the use of a rice microarray for an *S. propinquum* rhizome study is still worthwhile. Although some *S. propinquum* specific genes would be missed, any detected ones would probably represent multiple, evolutionarily conserved genes shared by *S. propinquum* and *O. sativa* [28]. Given the close phylogenetic relationship between the two species, the well-annotated rice genome and its known genome history [34], [35] can be exploited when profiling the tissue-specific genome expression of *S. propinquum.* In this manner, we can discover and characterize genes and putative pathways specifically responsible for rhizome initiation and elongation in sorghum.

## Materials and Methods

### Plant material and total RNA extraction

For this study, plants of a *S. propinquum* vegetative clone (unnamed accession) with vigorous rhizomes were cultured in the greenhouse. We collected samples at the vegetative growth stage as described by Hu et al. [18]. Five different tissues—rhizome tips (RT), rhizome internodes (RI), shoot tips (ST), shoot internodes (SI), and young leaves (YL)—were sampled for total RNA extraction. Three independent biological replicates for each sample from individual sorghum plants were collected and snap-frozen in liquid nitrogen. Total RNA was extracted using TRIzol reagent (Invitrogen), and then purified and concentrated using an RNeasy MinElute cleanup kit (Qiagen). RNA quality and concentrations were determined using a Bioanalyzer (Agilent).

### Microarray hybridization

Because no microarray platform is available for any *Sorghum* species, an Agilent rice gene expression microarray (product number: G2519F, 44K) was used in this study. The array contained 45,220 independent probes (60-mer) corresponding to 21,495 *O. sativa* mRNA sequences available in GenBank [34], [35], [36]. RNA amplification, labeling, and hybridization, and microarray imaging were carried out by a specialized biotech company, CapitalBio Corporation (Beijing, China). Equal amounts of RNA samples from five tissues (RT, RI, ST, SI or YL) were pooled and used as a common control. Each experimental sample and control (mix of the five experimental samples) labeled with Cy5-dCTP and Cy3-dCTP separately were produced by Eberwine's linear RNA amplification method and subsequent enzymatic reactions [37], [38]. Array hybridization was performed overnight at 42°C with 8 rpm rotation in a CapitalBio BioMixer II Hybridization Station, followed by two washes. Images were obtained with a confocal LuxScan scanner and then analyzed using LuxScan 3.0 software.

### Data analysis

For individual channel data extraction, faint spots with intensities below 400 units after background subtraction in both channels (Cy3 and Cy5) were removed, and a space- and intensity-dependent normalization based on the LOWESS algorithm [39] was then carried out. Significance analysis of microarrays (SAM, version 3.02, [40]) was performed to determine significantly differentially expressed genes. To identify tissue-enriched genes, the microarray data were first subjected to preliminary screening using the multiclass method in SAM with a false discovery rate (FDR) <5% used as a cutoff. The remaining data were then further screened to include only those genes with expression values for a given tissue showing more than 1.5-fold change compared with other tissues (*p* <0.05) in Wilcoxon rank-sum tests. The whole set of original microarray data has been deposited in NCBI's Gene Expression Omnibus and can be freely accessed through GEO Series number GSE40380.

### Functional classification and prediction of *cis*-acting regulatory elements for the tissue-specific genes

Hierarchical clustering was conducted by Cluster 3.0 and the ratio between experimental sample and control for the fifteen arrays were log transformed. Functional enrichment/overrepresentation analysis was carried out using the agriGO database (http://bioinfo.cau.edu.cn/agriGO/, [41]). GO Slim, representing a reduced version of the GO ontologies containing a subset of the terms in the whole GO were used. For overrepresentation determination, the FDR-adjusted significance level cutoff was set at 0.05. Mapping of rhizome-enriched genes to the 338 currently defined metabolic pathways in the RiceCyc database (http://www.gramene.org/pathway/) was accomplished using the Pathway Tools software package (version 15.5; [42]). *Cis*-elements of tissue-enriched genes were identified from both strands of upstream 1-kb promoter sequences retrieved from the rice homologous genes with the aid of the PLACE *cis*-element database (http://www.dna.affrc.go.jp/PLACE/) and a Perl program (‘regulatory’) provided by CapitalBio (CapitalBio Corporation, Beijing). To determine overrepresentation of putative *cis*-regulatory elements between two groups of genes, two-sample tests of proportion were conducted and the significance threshold was 0.05.

### Quantitative RT-PCR and RNA *in situ* hybridization

Quantitative real-time PCR was performed using an ABI Prism 7900 Sequence Detection System (Applied Biosystems). Diluted cDNA was amplified using gene specific primers and SYBR Green Master Mix (Applied Biosystems); expression levels of tissue-enriched transcripts were normalized with endogenous *Actin* transcripts. Each set of experiment was performed three times, and the delta-delta Ct (ddCt) relative quantification strategy was used to evaluate quantitative variation. Primers used are listed in Table S1.

*In situ* hybridization was carried out using the method described by Jackson [43]. Apical portions (1 cm long) of rhizomes were excised and fixed without RNase contamination. Two different templates were constructed by separately cloning Sb01g047010 and Sb06g028820 DNA coding sequences into pBluescript plasmids (Invitrogen). Gene primers used were Sb01g047010F (5′-CCCAGTGTTTTACGTGTATTGG-3′), Sb01g047010R (5′-CTTCATCTTTTTAACCTTGCTT-3′), Sb06g028820F (5′-TGGCAT CGTTGAGCACTGGGTG-3′), and Sb06g028820R (5′-GCCTGGGCAGGTTCATGTCTGG-3′). Following linearization of plasmids, antisense and sense RNA probes were transcribed by T3 and T7 RNA polymerase, respectively, in the presence of dig-UTP (Roche). Each experiment was performed three times using independent samples.

## Results

### Hybridization of *S. propinquum* cDNA to the rice microarray

As sufficient genomic data are available for both sorghum (*S. bicolor*) and rice (*O. sativa*), we remapped the microarray probes to the sorghum genome and found *S. bicolor* homologs for all of the rice genes on the microarray. The microarray data could therefore be used to provide information about gene expression in *S. propinquum*. After removing control probes, the remaining 43,803 independent probes corresponded to 21,495 *O. sativa* mRNA sequences in Genbank, with their homologs in sorghum then identified using the database at the Sorghum Genome Project (Department of Energy Joint Genome Institute, www.phytozome.net). Finally, 12,842 rice genes were found to be homologous to genes in *S. bicolor*; 10,147 (79.0%) of these possessed exactly one orthologous gene and 2,695 were in a one-to-many relation (data not shown). Of these, 3,406 independent probes on the rice array were mapped to the *S. bicolor* genome with 100% sequence identity, resulting in 1,703 *O. sativa* genes corresponding to 2,199 *S. bicolor* annotated genes (416 probes mapped to 2 or 3 *S. bicolor* genes, data not shown).

In this study, tissue from rhizome tips (RT), rhizome internodes (RI), shoot tips (ST), shoot internodes (SI), and young leaves (YL) of *S. propinquum* was chosen to discover specific sets of genes responsible for unique tissue development and growth. Of the genes on the rice microarray, 4,234 (10.1%) were found to be expressed in at least one tissue in this experiment (3,405 in RT, 3,803 in RI, 3,870 in ST, 3,509 in SI, and 3,452 in YL). Among these expressed genes, 2,760 were homologous to genes in *S. bicolor,* with 2,182 (79.1%) possessing exactly one orthologous gene and 578 in a one-to-many relation (data not shown). GO analysis demonstrated that genes detected in each tissue were all well-represented in every GO category, except for cellular nitrogen compound metabolic processes. In addition, 2,322 (5.1%) were present in all tissues, and 548 were specifically expressed in only one of the five tissues (Table S2, S3).

### Identification of tissue-specific gene expression in *S. propinquum*

**Figure 1:**
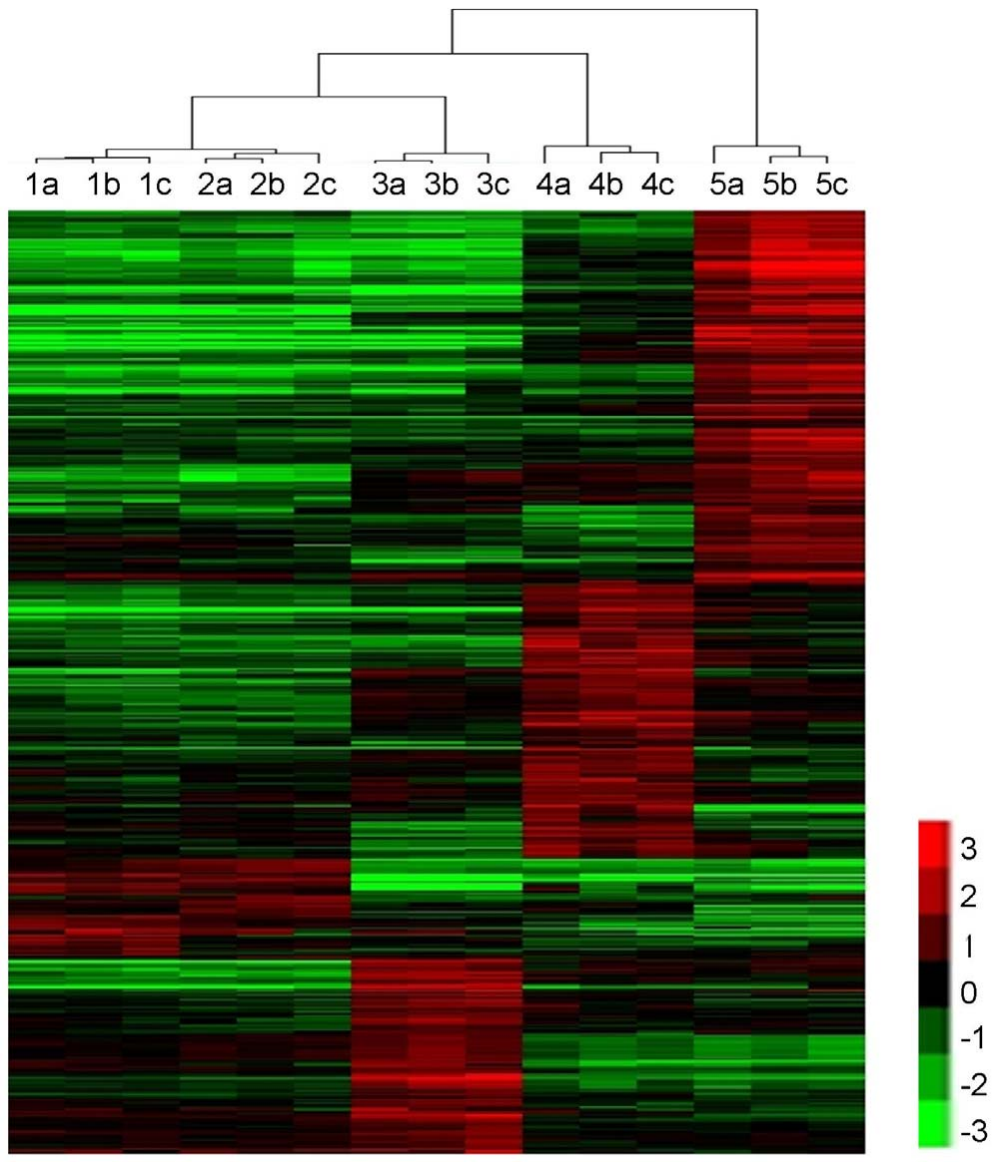
Dendrogram of 548 tissue-specifically expressed genes in five tissues of *Sorghum propinquum*. 1. Rhizome tips, 2. Shoot tips, 3. Rhizome internodes, 4. Stem internodes, 5. Young leaves. The suffixes a, b, and c indicate the three biological replicates. In the color panels, each horizontal line represents a single gene and the color of the line indicates the expression level (in a log scale) of the gene relative to the median in a specific sample: high expression in red, low expression in green. The raw data represented here are detailed in Table S3.

A total of 548 tissue-enriched genes were identified using SAM, including 31, 26, 114, 159, and 218 unique genes specifically enriched in RT, ST, RI, SI, and YL, respectively (Table 1; Table S3, S4, S5, S6, S7). Based on the Sorghum Genome Project, these rice genes on the array corresponded to 23, 14, 76, 106, and 133 annotated sorghum genes. With the exception of RT and ST tissues, which had nearly identical transcriptomic patterns, hierarchical clustering of gene expression profiles revealed that expression was tissue-driven (Figure 1 and Table S2). These results indicate that the identity of specific tissues is derived from their respective transcriptomes.

**Table 1:** The list of genes enriched specifically in rhizome tips relative to other tissues.

Oryza GI	FC^a^	q-value	Best Sorghum BLAST hit	Function Annotation
LOC_Os10g38020	1.66	0	Sb01g031130	expressed protein
LOC_Os03g07480	1.84	0	Sb01g045720	sucrose transporter
LOC_Os03g05480	2.54	0	Sb01g047010	Zinc finger, C2H2 type family protein
LOC_Os05g08600	1.79	0	Sb02g012970	pre-mRNA splicing factor PRP38 family protein
LOC_Os09g19910	1.55	0	Sb02g022990	expressed protein
LOC_Os08g33640	1.76	0	Sb02g024480	expressed protein
LOC_Os09g25810	1.75	0	Sb02g025200	Integral membrane protein DUF6 containing protein
LOC_Os07g33660	1.54	0	Sb02g034910	expressed protein
LOC_Os02g46750	1.52	0	Sb04g031140	expressed protein
LOC_Os02g52780	1.52	0	Sb04g034190	bZIP transcription factor family protein
LOC_Os11g39000	1.59	0	Sb05g023765	Helix-loop-helix DNA-binding domain containing protein
LOC_Os11g10800	2.13	0	Sb05g024940	dirigent-like protein, expressed
LOC_Os11g41670	1.59	0	Sb06g014780	expressed protein
LOC_Os01g54080	1.56	0	Sb06g019450	kinesin motor domain containing protein
LOC_Os04g52830	4.88	0	Sb06g028810	kelch repeat-containing F-box family protein
LOC_Os08g42950	1.94	0	Sb07g025430	haloacid dehalogenase-like hydrolase family protein
LOC_Os12g42700	1.82	0	Sb08g022070	expressed protein
LOC_Os05g02520	1.65	0	Sb09g001680	legumin, putative
LOC_Os05g10840	1.53	0.01	Sb09g006130	calmodulin-binding family protein
LOC_Os05g12474	1.55	0.03	Sb09g006935	expressed protein
LOC_Os05g39840	1.58	0	Sb09g023350	expressed protein
LOC_Os03g28960	1.75	0	Sb10g006995	DNA-directed RNA polymerase III 130 kDa polypeptide
LOC_Os02g10350	1.62	0	Sb10g023955	Mlo family protein, expressed
LOC_Os01g16250	1.55	0	unknown	expressed protein
LOC_Os09g07640	1.59	0	unknown	retrotransposon protein
LOC_Os01g01520	2.08	0	unknown	Transferase family protein
LOC_Os07g26100	1.86	0	unknown	expressed protein
LOC_Os02g33840	1.59	0	unknown	F-box domain containing protein
LOC_Os05g41870	1.77	0	unknown	glycine-rich cell wall protein
LOC_Os12g08760	1.55	0.07	unknown	Carboxyvinyl-carboxyphosphonate phosphorylmutase
LOC_OS03g05620	1.75	0	unknown	inorganic phosphate transporter

a FC:Fold Change, represents the ratio of Avg_RT vs. MAX (Avg_ST, Avg_RI, Avg_SI, and Avg_YL), and q-value (%) ≤5%, while Avg_x represents the average ratio of the three biological replicates while RT for Rhizome tips/control, ST for Shoot tips/control, RI for Rhizome internodes/control, SI for Stem internodes/control and YL for Young leaves/control.

Of the 218 genes highly enriched in the leaves, most were related to stimulus response, biological regulation, localization, metabolic processes, and cellular processes (Table S4). These included 17 genes encoding transcription factor proteins, such as WLIM1, auxin response factor, ethylene responsive element binding factor 1, and TCP family transcription factor, whose homologous genes were responsible for leaf differentiation in Arabidopsis [44]. In addition, a few genes, such as *JMJ706*, *FMO1*, *HRB1*, *FT*, and *GAMMA_CA2*, were found to be enriched in YL. Of these genes, *JMJ706*, which encodes heterochromatin-associated H3K9 demethylase, is involved in regulation of flower development in rice [45]. Functions for three genes have been identified in Arabidopsis: *FMO1*, encoding flavin-containing monooxygenase family protein, is critical for the development of systemic acquired resistance (SAR)[46], and *HRB1* and *GAMMA_CA2* have a modulatory role in the flowering pathway mediated by phyB and in photorespiration [47], [48].

A total of 26 enriched genes in ST were identified in our study (Table S5). These genes were found to be functionally associated with catalytic and binding activity. Among them, the LHC-related gene *LIL3:1* (Sb04g002190) plays an essential role in chlorophyll and tocopherol biosynthesis [49]. In addition, *KEG* (Sb09g019370), a regulator of abscisic acid signaling, and *CaS* (Sb04g029100), encoding a calcium-sensing receptor protein, are essential for plant growth and development [50], [51].

We detected 159 genes enriched in SI (Table S6); these genes included those involved in phytohormone signaling and metabolism, such as *SAUR10*, *IAA9*, the gibberellin receptor *GID1L2*, an auxin efflux carrier component (Sb03g029320), and two genes coding for AUX/IAA family proteins. Two circadian clock-related genes were also enriched in SI: an LHY-related gene (Sb06g026500) and Sb09g003090, a homolog of the Arabidopsis gene *PIF3* that functions in early phytochrome signaling at the dark-to-light transition [52]. In addition, based on near homologs reported in Arabidopsis and rice, a few genes related to plant growth and development, such as *WRKY70*, *PRMT11*, *APL2, GAPCP-2, PDAT, SAMC1, PTP1*, and *OsSub31,* were also determined to be highly enriched in SI, indicating their important role in shoot growth and development.

There were 113 genes identified as RI-enriched (Table S7). These included five genes functionally related to transport, including *ATCHX17, MST6, SPK1, TIM17-2*, and *ClCa*; seven genes related to cell wall biogenesis and the cell cycle, including *EXT3, WAK1, IRX10, ECI1, BTF3, VIM1*, and *PHS1*; two genes involved in oxidative damage and disease response (*GolS2* and *SGT1*); eight functioning in plant growth and development, including *CYP707As, NAM, GH3.9, OsLOG, NAC1, SEU, PTR2*, and *SSII*; and three genes related to photosynthesis, including *DAL1, CYP97A3*, and *PHOT2*. *CYP707As* encodes a cytochrome P450 family protein and is essential for proper control of seed dormancy and germination [53]. The rice *LONELY GUY* (*LOG*) gene is required to maintain meristem activity; its loss of function causes premature termination of the shoot meristem [54]. Previous reports have demonstrated that molecular mechanisms underlying cold temperature regulation of flowering time in Arabidopsis are controlled by *NAC1* [55].

A relatively small set of 31 genes were identified as enriched in RT (Table 1). These genes include *RELATIVE OF EARLY FLOWERING 6* (*REF6*, Sb01g047010), *AREB1,SUT1* (Sb01g045720), and *SUC3* (Sb09g006130). *REF6* functions as an FLC (*FLOWERING LOCUS C*) repressor in the regulation of Arabidopsis flowering [56], [57]. *AREB1*, a homologous gene of Sb04g034190, is a key positive regulator of ABA signaling in vegetative tissues of Arabidopsis under drought stress [58]. In addition, the two sucrose transporters *SUT1* and *SUC3* are involved in filling grain, germination, early seedling growth, and phloem loading of sucrose retrieved from the apoplast along the transport pathway [59], [60]. Furthermore, genes encoding kinesin protein, inorganic phosphate transporter, seven transmembrane MLO13, dirigent-like protein, glycine-rich cell wall protein, retrotransposon protein, and SRL1 (splicing factor) conferring biotic and abiotic stress tolerance, were also enriched.

### Identification of distinct *cis*-regulatory elements in tissue-enriched genes

Using the PLACE *cis*-element database and a Perl program, the *cis*-elements of tissue-enriched genes were identified on both strands of upstream 1-kb promoter sequences of rice homologous genes. A *cis*-element comparative analysis was performed on 29, 17, 86, and 117 genes enriched in RT, ST, RI, and SI, respectively, and several distinct elements were found between RT and ST, RI, and SI (Table 2).

**Table 2:** Identification of distinct *cis*-regulatory elements in the tissue-enriched genes.

*Cis*-element	RT (%)	ST (%)	p value^b^	Function
No. of tested genes	29	17		
C[ACGT]GTT[AG]	65.5	35.3	0.023	water stress
TGAC[CT]	62.1	88.2	0.029	wounding
AACGG	55.2	23.5	0.018	M phase
AACCAA	44.8	17.6	0.031	phytochrome
CATGCA	41.4	17.6	0.049	ABA
TTWTWTTWTT^a^	41.4	11.8	0.018	scaffold
ACGTG	37.9	64.7	0.04	ABRE
TTATCC	37.9	11.8	0.029	axillary
CATGCA[CT]	34.5	5.9	0.014	storage protein
TAACA[AG]A	31	5.9	0.023	amylase
TAACAA[AG]	27.6	5.9	0.037	GA
	RI (%)	SI (%)		
No. of tested genes	86	117		
[CT]TCA[ACGT]T[CT][CT]	67.4	53	0.019	initiater
TTATCC	26.7	16.2	0.034	axillary
AGCAGC	20.9	36.8	0.008	anaerobic
TGGGC[CT]	48.8	32.5	0.009	cytochrome

a W stands for [AT], ie A or T. ^b^P value represents the significance between RT and ST, or RI and SI.

Nine *cis-*elements were significantly more abundant in RT-enriched genes compared with ST, whereas two were more predominant in ST. The RT abundant *cis*-elements included the ABA-responsive RY repeat CATGCA, the sugar-repressive element TTATCC associated with axillary bud outgrowth, and the GA-responsive element TAACAA [AG] [61], [62], [63]. Interestingly, in a previous study [18] the RY repeat CATGCA was also found to be significantly more abundant in RT up-regulated genes than in the other four tissues of *S. propinquum*. In addition, three *cis*-elements were significantly more abundant in RI-enriched genes compared with SI: sugar-repressive element TTATCC, initiator element [CT] TCA [ACGT] T [CT] [CT], and Site II element TGGGC [CT] related to cytochrome [64].

### Rhizome-enriched genes in QTL regions related to rhizome traits

To identify rhizome-enriched genes corresponding to rhizome-related QTLs previously reported in *S. propinquum* [10], all identified RT- and RI-enriched genes were mapped onto the sorghum chromosomes. In a similar manner, the rice homologs of these rhizome-enriched genes in *S. propinquum* were mapped onto rhizome-related QTL regions of rice chromosomes identified by Hu et al. [16].

A total of 74 rhizome-enriched genes were physically mapped onto 11 rhizome-related QTL regions of sorghum. Among these, 9 genes were mapped onto rice rhizome-related QTLs (Table S8). Thirty-one rhizome-enriched genes were mapped onto the LAR QTL interval influencing the number of rhizomes producing above-ground shoots; these genes encode cell wall biogenesis and cell cycle related proteins such as BTF3, BAHD, SPIKE1, sucrose transporter (SUT1), and a protein required for starch breakdown (BAM4) [59], [65]. There were 59 rhizome-enriched genes mapped onto 8 QTL intervals (LSR) affecting subterranean rhizomatousness. Among these, there were three transporter protein encoding genes, including the sucrose transporter *SUC3,* the K+ transporter *CHX17*, and the silicon influx transporter *Lsi6* identified in rice [66]. Additional genes involved in plant growth and development, including *CYP707A1*, *OPR3*, and *SSII,* as well as *PHS1* related to the cell cycle, were also identified to be involved in LSR.

In addition, the rice homologues of 26 rhizome-enriched genes in *S. propinquum* were uniquely mapped onto 7 rice rhizome-related QTLs including the *Rhz3* interval. These genes include *NAM-B1* (Os07g37920), encoding a no apical meristem (NAM) protein, which was isolated as a QTL gene accelerating senescence and increasing nutrient remobilization from leaves to developing grains in wheat [67]. Another identified gene was *PTR4* (Os07g41330), encoding a peptide transporter, the homolog of which in Arabidopsis is the protein importer *AtTIM17-2* [68], [69]. All these genes should provide putative functional candidates for identified rhizome-related QTLs.

### Validation of tissue-enriched gene expression by quantitative RT-PCR and *in situ* hybridization

**Figure 2:**
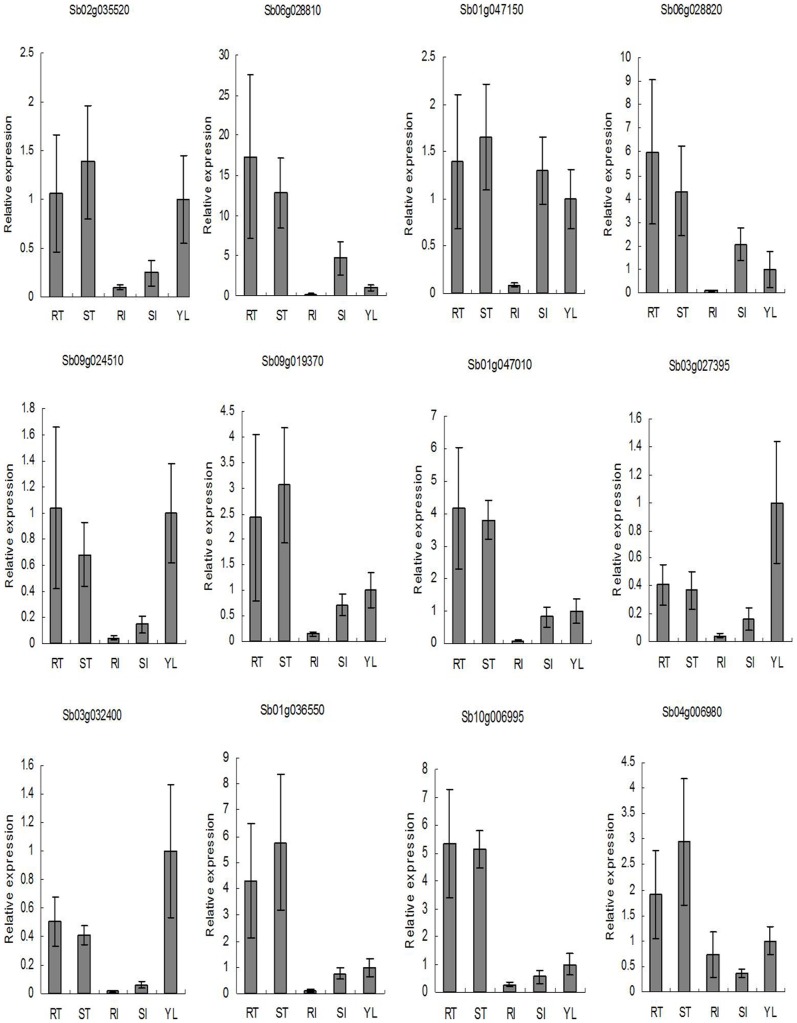
Real time PCR profiles of 12 selected tissue-enriched genes. RT, ST, RI, SI, and YL represent the rhizome tip, shoot tip, rhizome internodes, shoot internodes, and young leaves, respectively. Expression levels were calculated based on the expression level of YL genes set to 1. Expression profiles obtained by real time PCR for one gene, Sb01g036550, were not consistent with data obtained from microarray analysis. Correlation coefficients (r) for the remaining 11 genes were 0.74, 0.74, 0.74, 0.74, 0.77, 0.74, 0.82, 0.76, 0.76, 0.94, and 0.76, from left to right, respectively. Bars donate standard deviation.

To confirm the microarray data, 12 tissue-enriched genes were selected for quantitative RT-PCR analysis (Figure 2). This set included 5 RT-, 3 ST-, 1 RI-, 1 SI- and 2 YL-enriched genes. Overall, gene expression profiles of the five tissues detected by the microarray experiments were very similar to those obtained from the qRT-PCR analyses. Correlation coefficients (r) between 0.74 and 0.94 were calculated, thereby indicating the reliability and robustness of the microarray data.

**Figure 3:**
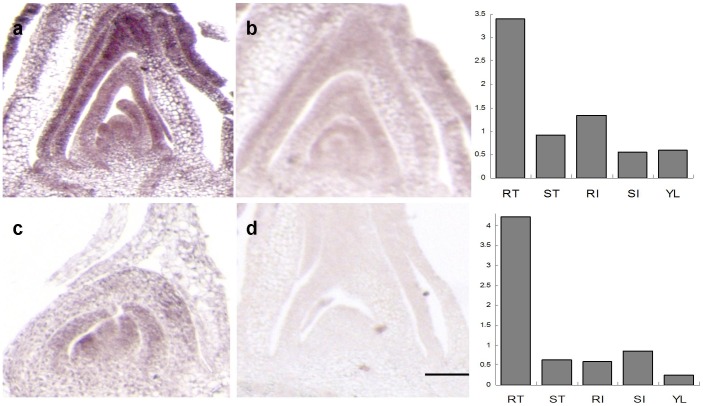
Validation of microarray data by *in situ* hybridization. *In situ* localization of transcripts corresponding to the genes (a) Sb01g047010 and (c) Sb06g028820 in *S. propinquum* rhizome tips are illustrated; (b) and (d) represent the sense probe for control. Corresponding microarray-based expression profiles of these two genes are also shown as bar graphs for comparison.

We additionally validated our microarray expression results by *in situ* hybridization analysis of two *S. propinquum* RT-enriched genes (Figure 3). The transcript of Sb01g047010, which codes for a C2H2-type family protein, was highly expressed in the RT apical meristem. Another gene (Sb06g028820), encoding a kelch repeat-containing F-box family protein, exhibited slightly lower expression in the RT apical meristem. The *in situ* expression patterns of these two genes confirm the *S. propinquum* RT-enriched profiles obtained from the microarray analysis.

### Comparative analysis of rhizome-enriched genes in *O. longistaminata* and *S. propinquum*

We comparatively analyzed the rhizome-enriched genes identified in this study along with genes characterized as rhizome-specifically or differentially expressed genes (DEGs) in previous studies. Genes used for comparison were those found in *O. longistaminata* using transcriptome sequencing (Li et al., unpublished) and Affymetrix microarray analysis [18], and in *S. propinquum* based on a cDNA macroarray analysis [11].

**Figure 4:**
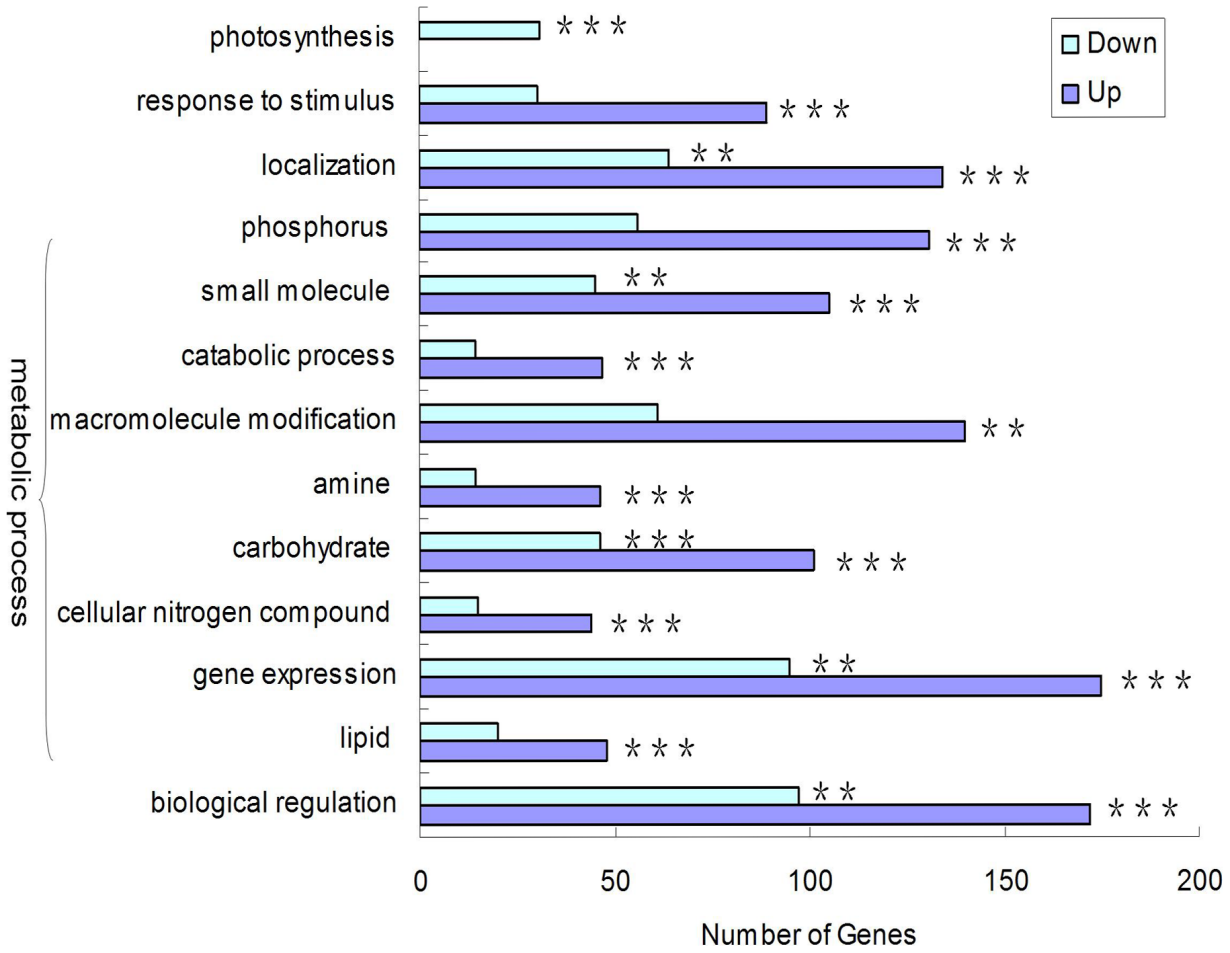
GO slim categories in up- and down-regulated RT genes combined from this experiment and three other reported studies. Bars show number of genes with significantly higher relative transcript abundance. All GO slim categories significantly over- or underrepresented are calculated based on a hypergeometric distribution. Significant over- or under-represented categories are indicated by * for *p* ≤ 0.05, ** for *p* ≤ 0.01, and *** for *p* ≤ 0.001.

All these DEGs, comprising 1,856 up-regulated and 1,172 down-regulated genes in rhizomes, were first combined for GO analysis (Figure 4). Results indicated that genes related to photosynthesis were significantly abundant only in the down-regulated set, while genes related to stimulus response and metabolic processes, including cellular nitrogen compound metabolic processes and lipid metabolic processes, were significantly represented only in the up-regulated group. GO Slim terms such as localization, metabolic processes (including small molecule metabolic processes and carbohydrate metabolic processes, gene expression), and biological regulation, were significant in both up- and down-regulated genes. In addition to the GO analysis, all rhizome up-regulated genes were mapped to currently defined metabolic pathways in the RiceCyc database (http://www.gramene.org/pathway/). Many different metabolic pathways were broadly represented at every step by rhizome up-regulated genes. In contrast, secondary metabolic pathways and cofactors, prosthetic groups, and electron carrier biosynthesis showed the least representation by RT up-regulated genes (Figure S1). This suggests that major metabolic pathways are involved in RT formation and development.

**Figure 5:**
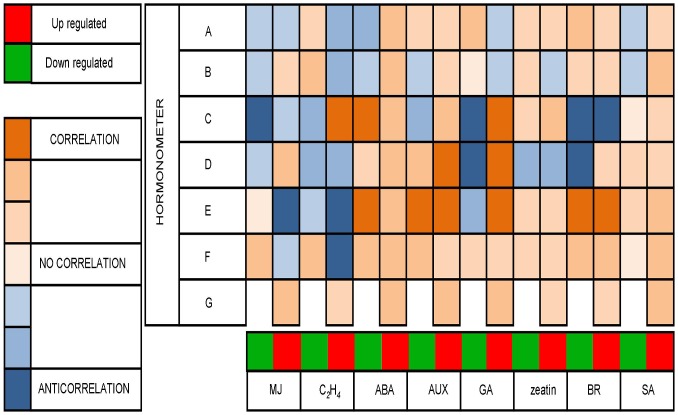
Heat map showing the relationship between rhizome related differentially expressed genes (DEGs) and hormone target genes. The heat map was produced by analyzing genes comprising rhizome related DEGs for methyl jasmonate (MJ), ethylene (C*2*H*4*), abscisic acid (ABA), auxin (AUX), gibberellic acid (GA), zeatin, brassinosteroids (BR), and salicylic acid (SA). Subfigures a–g represent the seven gene sets analyzed: DEGs of RT vs. ST in *O. longistaminata* from (a) transcriptome sequencing and (c) microarray analysis, and (e) results of the present study in *S. propinquum*; DEGs of underground tissues (RT and RI) vs. above-ground tissues (ST, SI, and YL) in *O. longistaminata* from (b) transcriptome sequencing and (d) microarray analysis, and (f) results of the present study in *S. propinquum*; and (g) candidate rhizome-enriched genes in *S. halepense* (pSH) and *S. propinquum*. In the HORMONOMETER analysis, orange (1) = complete correlation, white (0) = no correlation, and blue (-1) = anti-correlation.

Hormone-related genes possibly involved in cross-talk during rhizome formation and development were identified using HORMONOMETER analyses (http://genome.weizmann.ac.il/hormonometer/, [70]). Because HORMONOMETER is based upon a list of hormone indexes available for Arabidopsis, we aligned all DEGs to the Arabidopsis genome using Phytozome v7.0 before starting the analysis. A heat map was produced by analyzing the correlation between rhizome up- and down-regulated genes in all studied gene sets and a curated set of ATH1 arrays for different hormone treatments (Figure 5). Significant correlation was found between up-regulated genes and transcriptional response to abscisic acid (ABA), auxin, gibberellic acid (GA), and salicylic acid (SA).

Using the comparative genomics tool Phytozome v7.0 (http://www.phytozome.net/), all the rhizome-enriched genes identified in our heterologous microarray study were compared with rhizome-enriched genes or DEGs detected with the other three platforms. There were a number of genes showing the same expression pattern in at least two different platforms (Table 3, S9); 43 of these exhibited rhizome-enriched expression patters (Table 3) and 61 were repressed (Table S9). As shown in Table 3, two genes encoding NAM proteins (Os01g64310 and Os07g37920) were found to be highly rhizome-enriched in the three gene sets. Genes related to transcription regulation and signal transduction, including WRKY TF, eIF6, protein kinase, and HSP, were identified as rhizome-enriched genes in at least two experiments, indicating their important role in rhizome growth and development.

**Table 3:** The list of up regulated genes in the comparison of the rhizome related DEGs.

Oryza ID	E^a^	F	A	B	C	G	Annotation
LOC_Os10g38850	4					Up	Adrenodoxin reductase family protein
LOC_Os05g06660	2.8			1.3			Peptidase S10, serine carboxypeptidase family protein
LOC_Os03g27590	2.21		2.45				Peptidase S10, serine carboxypeptidase family protein
LOC_Os02g44770	2.13			1.87			MscS Mechanosensitive ion channel family protein
LOC_Os06g25250	2.02			3.74			Ribonuclease III domain containing protein
LOC_Os02g56250	1.86			1.53			Conserved hypothetical protein
LOC_Os09g39960	1.83			1.74			Dynamin-like protein 4 (ADL4)
LOC_Os12g08760	1.77		1.63				Isocitrate lyase and phosphorylmutase family protein
LOC_Os09g28230	1.72		3.45	1.68			Esterase/lipase/thioesterase domain containing protein
LOC_Os03g21710	1.7		2.61	1			WRKY DNA binding protein
LOC_Os09g20000	1.59	1.63	1.86	1.24			Heavy metal transport/detoxification protein
LOC_Os01g17330	1.59			4.45			Eukaryotic translation initiation factor 6 (eIF-6)
LOC_Os07g48100	1.58		1.16	2.3		Up	Serine/threonine protein kinase
LOC_Os02g27760	1.57					Up	40S ribosomal protein S15a
LOC_Os02g43430	1.53			1.42			Protein serine/threonine kinase
LOC_Os01g73470	1.53			1.17			Conserved hypothetical protein
LOC_Os07g37920	1.53	2.75		9.9			No apical meristem (NAM) protein
LOC_Os04g32920		2.56		2.67			Potassium transporter 5 (AtPOT5)
LOC_Os06g07630		2.3				Up	26S protease regulatory subunit 6A
LOC_Os02g05330		2.19				Up	Eukaryotic initiation factor 4A (eIF4A)
LOC_Os08g03020		2.18		3.53			Resistance protein candidate (Fragment)
LOC_Os04g39410		2.12		1.71			TPR-like domain containing protein
LOC_Os11g47830		2.09		1.28			RNA-binding region RNP-1
LOC_Os05g01280		1.95	3.32				AT.I.24-5 protein (Fragment)
LOC_Os11g05190		1.9		1.79			Phytosulfokines 2 precursor
LOC_Os04g01740		1.89		5.3			Heat shock protein 81-1 (HSP81-1)
LOC_Os02g12420		1.87		1.15			Protein kinase domain containing protein
LOC_Os08g37520		1.86		1.51			TPR-like domain containing protein
LOC_Os12g31850		1.85	1.7				Allantoin permease
LOC_Os05g02060		1.8		1.34			Amino acid selective channel protein
LOC_Os01g59920		1.78		1.18			Cysteine synthase, chloroplast precursor
LOC_Os03g05260		1.78				Up	Ankyrin repeat containing protein
LOC_Os02g07680		1.69	1.24	1.41			Cytochrome P450 family protein
LOC_Os08g30820		1.67		1.04			Conserved hypothetical protein
LOC_Os05g45350		1.65		3.86			Heat shock protein DnaJ family protein
LOC_Os05g43970		1.65		2.61			28 kDa heat- and acid-stable phosphoprotein
LOC_Os03g10210		1.65	1.17		4.04		Homeodomain leucine zipper protein CPHB-7
LOC_Os01g64310		1.63		2.3	4.35		No apical meristem (NAM) protein
LOC_Os08g36310		1.58	2.11	2.37			E-class P450, group I family protein
LOC_Os06g46030		1.58			2.2		Ribulose bisphosphate carboxylase
LOC_Os04g35540		1.51	2.74	2.52			Amino acid/polyamine transporter I family protein
LOC_Os07g14150	1.51			1.17			Nitrogen fixation protein

a A∼G represent the expression level of the 7 gene sets including DEGs of rhizome tip verse shoot tip in *O. longistaminata* from transcriptome sequencing data (A), microarray analysis data (C) and results of the present study in *Sorghum propinquum* (E), DEGs of underground tissues (rhizome tip and rhizome internode) verse above ground tissues (shoot tip, shoot internode and young leaf) in *O. longistaminata* from transcriptome analysis (B), microarray analysis (D) and results of the present study in *Sorghum propinquum* (F), and candidate rhizome-enriched genes in *S. Halepense* (pSH) and *S. propinquum* (G).

We further analyzed the *cis*-elements of these identified rhizome DEGs, and found that the sugar-repressive element TTATCC, related to axillary bud outgrowth and previously characterized as an RT- and RI-abundant *cis*-element [71], was significantly more abundant in the up-regulated genes (Table 4). A GA-responsive element, TATCCA, was also predominantly enriched in the rhizome up-regulated genes; this is consistent with the results of an earlier study in which the GA-responsive element TAACAA [AG] was found to be abundant in RT [72]. In addition, the CTCTT motif related to nodulin and the cytokinin responsive element TATTAG [73], [74] were abundantly represented in the up-regulated genes. In contrast, two motifs related to root CAACA and [GT] CACG [TA] [75], [76] were more abundant in the down-regulated gene sets.

**Table 4:** Identification of distinct *cis*-regulatory elements in the DEGs shown the same expression pattern in at least two different platforms.

*Cis*-element	Up (%)	Down (%)	p value^a^	Function
No. of tested genes	40	53		
CTCTT	85	67.9	0.029	nodulin
TATCCA	30	11.3	0.012	gibberellin
TTATCC	35	13.2	0.006	axillary bud outgrowth
TATTAG	27.5	13.2	0.042	cytokinin
CAACA	55	73.6	0.031	root; leaf; shoot
[GT]CACG[TA]	27.5	49.1	0.018	root; hair

a P value represents the significance between up regulated and down regulated genes.

## Discussion

Rhizomes are organs of fundamental importance to plant competitiveness and invasiveness, playing the contrasting roles of overwintering and vegetative propagation in perennial grasses such as *O. longistaminata* and *S. propinquum*. A thorough understanding of the molecular mechanisms of rhizome initiation and elongation can aid in the development of perennial rhizomatous grain crops and improve agricultural productivity. In this study, we used an Agilent rice gene expression microarray to profile the tissue-specific genome expression of *S. propinquum*. The goal of our study was the discovery and characterization of genes and putative pathways specifically responsible for rhizome development in sorghum. Utilizing the well-studied rice genome, we identified two distinct sets of genes enriched in RT and RI, and explored their functional annotation, regulatory motifs, and association with QTLs conferring rhizomatousness.

Several reports have demonstrated that the usefulness of heterologous microarrays in the investigation of gene expression in certain species for which unique microarrays had not yet been developed. Wang et al. [17] identified six genes related to the development of the bamboo rhizome bud based on rice cross-species microarray hybridization; Bagnaresi et al. [77] used a tomato microarray to analyse tuber transcriptome in potato. In both caes, the organ-specific gene expression was confirmed by *q*PCR analysis. Because the heterologous oligonucleotide microarray analysis would certainly miss some *S. propinquum* specific genes, a comparison of rhizome-enriched genes in *O. longistaminata* and *S. propinquum —* derived from our study as well as previous studies—was also carried out to analyze and categorize genes by function, pathway metabolism, hormone response, and regulatory motif. Although the small set of rhizome-specifically and differentially expressed genes detected in this study is incomplete, the data represent an important contribution to the determination of rhizome formation and development in *S. propinquum*. Detailed examination and comparative analysis of the functions of these genes should provide insights into molecular mechanisms associated with *S. propinquum* rhizome development and growth.

While gene expression patterns in the two studied distinct rhizome regions (RT and RI) were not very similar, expression levels in RT more closely resembled those in the above-ground plant part ST. In a previous study of *S. propinquum*, Jang et al. [11] also found that expression patterns in RT more closely resembled those in ST than those in RI. Because RT and ST are actively growing organs and virtually the entire rhizome tip eventually emerges from the ground to become a shoot tip, it is not surprising that RT and ST show similar expression patterns. Our observation that RT and RI have extremely different expression patterns is consistent with the fact that while RT is an actively growing organ, RI is largely a storage organ with new buds springing up from the joint of rhizome internodes only occasionally.

The rhizome tip is the most important tissue for rhizome initiation and development, with rhizome internodes playing a lesser but still important role. A small portion of these tissue-enriched genes without annotation in sorghum might result from incomplete annotation of sorghum genome or sequence diversity between *S. propinquum* and reference *S. bicolor*. RT- and RI-enriched genes, both of known (including homologs) and unknown function are therefore important candidates for further study. Unlike RI-enriched genes, the function of most RT-enriched genes, other than an *AREB1* homologous gene, an *REF6* homologous gene, and two sucrose transporters, is unknown. *AREB1* is active in ABA signaling, suggesting that this hormone plays a part in rhizome initiation and development. Most RI-enriched genes function in major metabolic pathways, including transport, cell wall biogenesis, cell cycle, and plant growth and development, indicating the fundamental role RI plays as a storage organ.

Several RT- and RI-enriched genes, however, have no discernible function in rhizome growth and development. These include the RT-enriched gene *REF6* functioning in the flowering pathway and three RI-enriched photosynthesis genes that are related to *NAC1*, another flowering pathway conferring gene. When RT up-regulated genes were compared for pathway analysis, five genes were identified that are involved in photorespiration: Os12g22030, Os11g26860, Os05g35440, Os03g52841, and Os01g65410 (Figure S1). Photosynthesis- and flowering-related genes, which function in the presence of light or in chloroplasts, should not be enriched in underground tissues. Other researchers [11], [31], [78] have speculated that these incongruities are due to ancient duplication of the transcriptome followed by subfunctionalization of expression patterns. However, the gene *FLOWERING LOCUS T* controls both flowering and storage organ formation in potato [79]. Considering that the rhizome eventually transforms into a shoot, perhaps some genes related to flowering and photosynthesis also function in rhizome initiation and development, possibly during the rhizome-to-shoot transition. This phenomenon requires further study.

Identification of *cis*-regulatory elements in tissue-enriched genes revealed that the ABA-responsive RY repeat CATGCA and the GA-responsive element TAACAA [AG] were abundant in RT, implying cross-talk of plant hormones during rhizome development. In addition, several other elements were significantly abundant in RT-enriched genes, i.e., sugar-repressive element TTATCC involved in axillary bud outgrowth, Myb core motif AACGG related to the M phase of the cell cycle, T-Box TT[AT]T[AT]TT[AT]TT found in the scaffold attachment region, AACCAA motif required for phytochrome regulation, RY repeat CATGCA [CT] relevant to storage protein, and amylase box TAACA [AG] A related to amylase. The presence of these elements indicates that compared with ST, RT cells are more active during developmental stages such as bud outgrowth and cell division [80], [81], [82], [83], [84]. In addition, the motif C [ACGT] GTT [AG], responsive to water stress, was also abundant in RT, whereas the W box TGAC [CT], involved in wounding, and the ABRE-like sequence ACGTG, related to early response to dehydration, were abundant in ST. This demonstrates that environmental responses of subterranean and aerial plant tissues differ in the types of signaling proteins involved [85], [86], [87]. Unlike the tip tissues, there were only three motifs significantly more abundant in RI than in SI. These were sugar-repressive element TTATCC related to axillary bud outgrowth, initiator element [CT] TCA [ACGT] T [CT] [CT], and Site II element TGGGC [CT] related to cytochrome. These also emphasize the fundamental role of RI as a storage organ.

Several lines of evidence point to plant hormones, such as ABA, GA, auxin, cytokinin, and SA, as key regulators of rhizome gene expression and development. Jang et al. [11] reported that a GA responsive *cis*-element was RT enriched in relatively highly expressed genes, implicating GA in regulation of many rhizome-specific genes. Hu et al. [18] found that several genes involved in GA biosynthesis were highly enriched in RT as compared to ST, while *cis-*elements related to auxin, JA, and ABA were abundant in rhizome-enriched genes. In our study, genes active in ABA signaling were RT enriched, and the *cis-*elements ABA-responsive RY repeat CATGCA and GA-responsive element TAACAA [AG] were significantly abundant in RT-enriched genes. Comparative analysis of rhizome-enriched genes revealed that hormone biosynthesis was the predominant pathway of integrated RT up-regulated genes in *S. propinquum* and *O. longistaminata*. According to HORMONOMETER analyses, the rhizome up-regulated genes were significantly correlated with transcriptional responses to ABA, GA, and SA, indicating that phytohormones may play important roles in rhizome initiation and development.

In this study, 74 rhizome-enriched genes co-localized with 11 rhizome-related QTL regions of sorghum, and 26 genes co-localized with 7 rice rhizome-related QTLs. These rhizome-enriched genes identified by our cross-species microarray represent multiple, evolutionarily conserved genes between *S. propinquum* and *O. sativa*, and appear to be heavily involved in rhizome development in perennial grasses. In addition, the 104 DEGs showing the same expression pattern on at least two different platforms, especially the 43 up-regulated ones, confirmed expression levels in the same species as well as those of evolutionarily conserved genes in both rice and sorghum.

In conclusion, a whole rice genome oligonucleotide microarray was used to profile gene expression across five tissues of the perennial wild sorghum *S. propinquum*. Expression patterns of the five tissues were consistent with the different functions of each organ, and RT- and RI-enriched genes revealed clues to molecular mechanisms of rhizome development. Plant hormones, including ABA, GA, and SA, function as key regulators of rhizome gene expression and development. To shed further light on the identities of rhizome-specific genes, rhizome-enriched candidates were identified using QTL co-localization and comparative analysis.

## Supporting Information

Figure S1 **An overview of rhizome tip up regulated genes in** ***Sorghum propinquum*** **and** ***O. longistaminata*** **mapped to major metabolic pathways in rice (ssp.** ***japonica*** **).**
(PPT)

Table S1 **Primer list for the real-time PCR analysis.**
(DOC)

Table S2 **Commonly and uniquely expressed genes in the five tissues.**
(DOC)

Table S3 **A complete list of 548 differentially expressed genes in five tissues of** ***Sorghum propinquum*** **.**
(DOC)

Table S4 **The list of genes enriched specifically in the young leaf relative to other tissues.**
(DOC)

Table S5 **The list of genes enriched specifically in shoot tips relative to other tissues.**
(DOC)

Table S6 **The list of genes enriched specifically in shoot internodes relative to other tissues.**
(DOC)

Table S7 **The list of genes enriched specifically in rhizome internodes relative to other tissues.**
(DOC)

Table S8 **Rhizome-enriched genes on the rhizome-related QTLs regions identified in sorghum and rice.**
(DOC)

Table S9 **The list of down regulated genes in the comparison of the rhizome related DEGs.**
(DOC)
