# Supplementary material for: Tissue-Specific Transcriptomic Profiling of Sorghum propinquum using a Rice Genome Array
Source: PLoS One. 2013 Mar 25;8(3):e60202. doi: 10.1371/journal.pone.0060202 (PMC3607598; doi:10.1371/journal.pone.0060202)
Supplement: Figure S1 — An overview of rhizome tip up regulated genes in Sorghum propinquum and O. longistaminata mapped to major metabolic pathways in rice (ssp. japonica ). (PPT) [file pone.0060202.s001.ppt]

## Slide 1
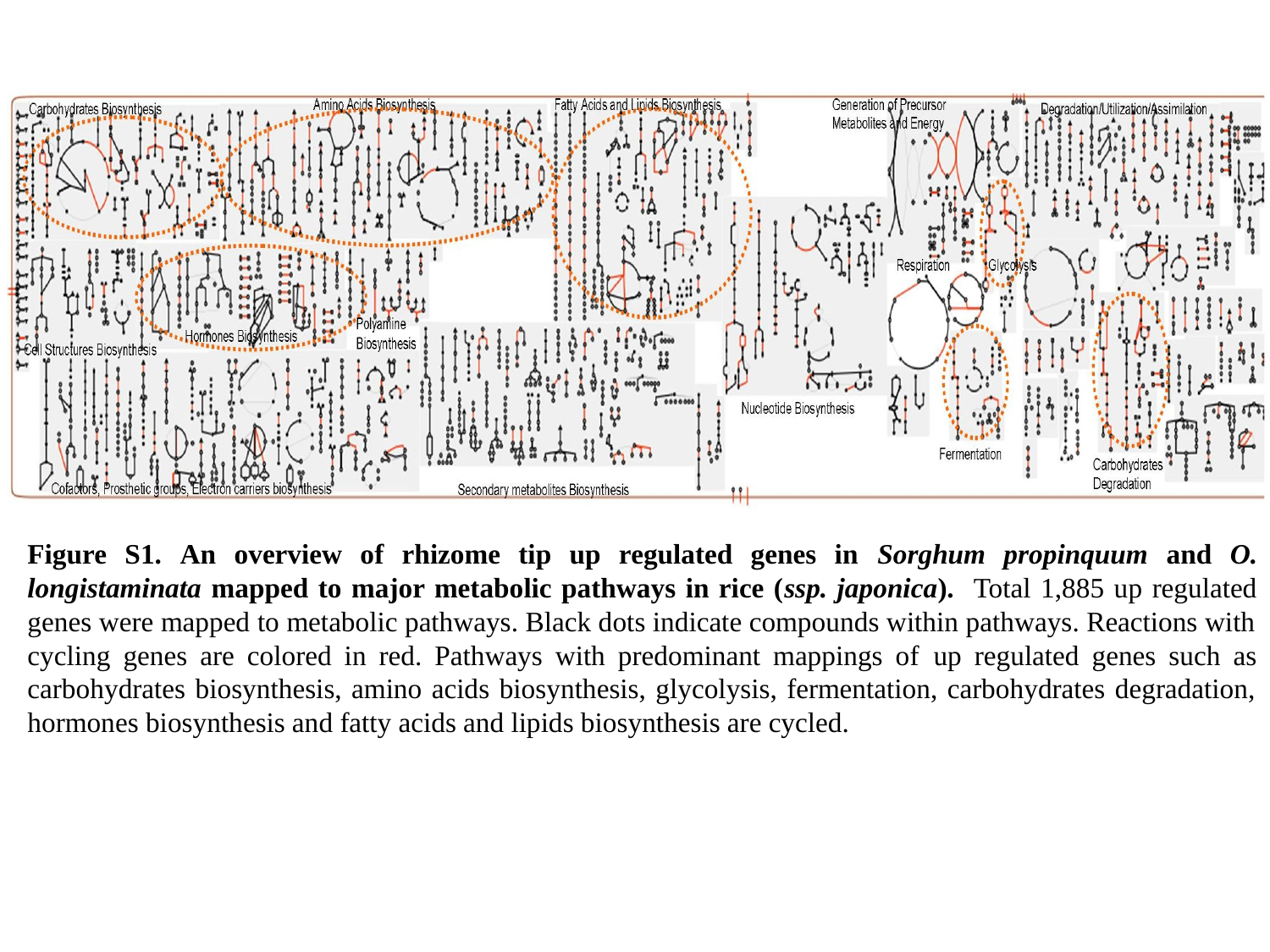

Figure S1. An overview of rhizome tip up regulated genes in Sorghum propinquum and O. longistaminata mapped to major metabolic pathways in rice (ssp. japonica). Total 1,885 up regulated genes were mapped to metabolic pathways. Black dots indicate compounds within pathways. Reactions with cycling genes are colored in red. Pathways with predominant mappings of up regulated genes such as carbohydrates biosynthesis, amino acids biosynthesis, glycolysis, fermentation, carbohydrates degradation, hormones biosynthesis and fatty acids and lipids biosynthesis are cycled.
