# Supplementary material for: Tissue-Specific Transcriptomic Profiling of Sorghum propinquum using a Rice Genome Array
Source: PLoS One. 2013 Mar 25;8(3):e60202. doi: 10.1371/journal.pone.0060202 (PMC3607598; doi:10.1371/journal.pone.0060202)
Supplement: Table S1 — Primer list for the real-time PCR analysis. (DOC) [file pone.0060202.s002.doc]

**Table S1.** Primer list for the real-time PCR analysis.

| **Gene** | **Primer** | **Sequence** |
| --- | --- | --- |
| Sb01g047010 | Sb01g047010F | 5' CCCAGTGTTTTACGTGTATTGG 3' |
|  | Sb01g047010R | 5' CTTCATCTTTTTAACCTTGCTT 3' |
| Sb03g027395 | Sb03g027395F | 5' GCTTTCTTTCGGCATCAGCG 3' |
|  | Sb03g027395R | 5' ATACCCAGCAATCATCAAGTTCC 3' |
| Sb03g032400 | Sb03g032400F | 5' GAGAATAGATTTGCGTTCACCA 3' |
|  | Sb03g032400R | 5' GAAATAAGGACCAGGCTTGATA 3' |
| Sb01g036550 | Sb01g036550F | 5' TCATTGATGCCACATACTACCG 3' |
|  | Sb01g036550R | 5' AAAATATCCTTTCCCTCCTGCT 3' |
| Sb10g006995 | Sb10g006995F | 5' TGAGTATCTTAGGGGTTGTATC 3' |
|  | Sb10g006995R | 5' ATCTTTATCTCTTTGGGGTTTA 3' |
| Sb04g006980 | Sb04g006980F | 5' GTATTCGTTTTGGATGAGGTGG 3' |
|  | Sb04g006980R | 5' CTGATTTGGTTGGTCTGTTTGG 3' |
| Sb02g035520 | Sb02g035520F | 5' CCCATGCTAGGGTTCCTGGAGA 3' |
|  | Sb02g035520R | 5' GCACGCGGTTGATGACGTTGTT 3' |
| Sb06g028810 | Sb06g028810F | 5' TGGGTGTGATAAGAATGGACAG 3' |
|  | Sb06g028810R | 5' ACAAGAGGAGGCGATTGAGATG 3' |
| Sb01g047150 | Sb01g047150F | 5' GGGGTGCTCATGGGATTATTCT 3' |
|  | Sb01g047150R | 5' TTCTCCACGTTTTCTCTTGTTT 3' |
| Sb06g028820 | Sb06g028820F | 5' TGGCATCGTTGAGCACTGGGTG 3' |
|  | Sb06g028820R | 5' GCCTGGGCAGGTTCATGTCTGG 3' |
| Sb09g024510 | Sb09g024510F | 5' GGTGCCCTCCTACTGCTCCTCC 3' |
|  | Sb09g024510R | 5' CCACTCGATCCGCTGCTGCTAA 3' |
| Sb09g019370 | Sb09g019370F | 5' TGATGGAAACACTTGAAGATAA 3' |
|  | Sb09g019370R | 5' CTACAACAGACTGGACAAAACC 3' |
| actin | actinF | 5' GACCTCACCGACCACCTAATGAA 3' |
|  | actinR | 5' AGAGAAGCCAAAATAGAGCCACC 3' |
