# Supplementary material for: Tissue-Specific Transcriptomic Profiling of Sorghum propinquum using a Rice Genome Array
Source: PLoS One. 2013 Mar 25;8(3):e60202. doi: 10.1371/journal.pone.0060202 (PMC3607598; doi:10.1371/journal.pone.0060202)
Supplement: Table S2 — Commonly and uniquely expressed genes in the five tissues. (DOC) [file pone.0060202.s003.doc]

**Table S2.** Commonly and uniquely expressed genes in the five tissues of *Sorghum propinquum* detected by the Agilent oligomer chips.

| **Tissue** | **N_1_^a^** | **N_2_** | **vs ST^b^** | **vs RI** | **vs SI** | **vs YL** |
| --- | --- | --- | --- | --- | --- | --- |
| Rhizome tips (RT) | 3405 | 31 | 344 | 1091 | 1343 | 1453 |
| Shoot tips (ST) | 3870 | 26 | - | 1225 | 1478 | 1531 |
| Rhizome internodes (RI) | 3803 | 114 | - | - | 1210 | 1324 |
| Shoot internodes (SI) | 3509 | 159 | - | - | - | 803 |
| Young leaves (YL) | 3452 | 218 | - | - | - | - |
| Total | 4234 | 548 | - | - | - | - |

^a^ N_1_ and N_2_ are the numbers of the total expressed genes and tissue-specifically expressed genes, respectively. ^b^ Total number of differentially expressed genes between two tissues.
