# Supplementary material for: Tissue-Specific Transcriptomic Profiling of Sorghum propinquum using a Rice Genome Array
Source: PLoS One. 2013 Mar 25;8(3):e60202. doi: 10.1371/journal.pone.0060202 (PMC3607598; doi:10.1371/journal.pone.0060202)
Supplement: Table S3 — A complete list of 548 differentially expressed genes in five tissues of Sorghum propinquum . (DOC) [file pone.0060202.s004.doc]

***Table S3.*** *A complete list of 548 differentially expressed genes in five tissues of Sorghum propinquum.*

| **Name** | **q-value(%)^a^** | **Fold Change^b^** | **Special Group^c^** | **Avg_A^d^** | **Avg_B** | **Avg_C** | **Avg_D** | **Avg_E** |
| --- | --- | --- | --- | --- | --- | --- | --- | --- |
| AK066454 | 0.00 | 12.65 | C | 0.55 | 0.39 | 6.90 | 0.32 | 0.22 |
| AK107519 | 0.00 | 12.04 | C | 0.57 | 0.37 | 6.88 | 0.36 | 0.22 |
| AK109814 | 0.00 | 1.52 | B | 1.44 | 2.19 | 0.04 | 0.19 | 0.81 |
| AK061292 | 0.00 | 4.82 | E | 0.02 | 0.05 | 0.03 | 1.61 | 7.78 |
| AK062526 | 0.00 | 5.72 | E | 0.03 | 0.03 | 0.03 | 0.80 | 4.59 |
| AK073528 | 0.00 | 1.82 | A | 3.00 | 1.65 | 0.03 | 0.52 | 0.24 |
| AK106415 | 0.00 | 5.00 | E | 0.22 | 0.17 | 0.50 | 0.83 | 4.13 |
| AK107975 | 0.00 | 1.55 | A | 2.80 | 1.81 | 0.04 | 0.57 | 0.21 |
| AK108674 | 0.00 | 7.44 | E | 0.25 | 0.24 | 0.07 | 0.48 | 3.57 |
| AK067292 | 0.00 | 1.86 | B | 1.24 | 2.31 | 0.83 | 1.15 | 0.04 |
| AK058798 | 0.00 | 2.22 | D | 0.03 | 0.04 | 0.09 | 3.13 | 1.41 |
| AK067445 | 0.00 | 2.24 | D | 0.05 | 0.05 | 0.11 | 3.88 | 1.73 |
| AK074000 | 0.00 | 9.07 | E | 0.26 | 0.21 | 0.23 | 0.44 | 4.00 |
| AK069053 | 0.00 | 4.83 | E | 0.11 | 0.18 | 0.13 | 1.33 | 6.43 |
| AK106439 | 0.00 | 2.45 | D | 0.43 | 0.76 | 1.89 | 4.63 | 0.34 |
| AK108458 | 0.00 | 1.84 | B | 1.39 | 2.56 | 0.08 | 0.16 | 0.38 |
| AK061089 | 0.00 | 13.68 | E | 0.05 | 0.02 | 0.09 | 0.54 | 7.41 |
| AK073869 | 0.00 | 4.25 | C | 0.08 | 0.03 | 4.65 | 0.78 | 1.09 |
| AK103452 | 0.00 | 2.05 | E | 0.46 | 0.33 | 0.11 | 1.31 | 2.69 |
| AK060537 | 0.00 | 7.73 | E | 0.02 | 0.02 | 0.13 | 0.67 | 5.21 |
| AK111879 | 0.00 | 2.84 | D | 0.23 | 0.17 | 0.26 | 6.72 | 2.37 |
| AK063764 | 0.00 | 1.57 | B | 1.53 | 2.41 | 0.30 | 0.30 | 0.28 |
| AK061510 | 0.00 | 4.26 | E | 0.07 | 0.09 | 0.13 | 1.06 | 4.51 |
| AK062372 | 0.00 | 9.37 | E | 0.49 | 0.38 | 0.19 | 0.40 | 4.54 |
| AK099349 | 0.00 | 338.89 | E | 0.02 | 0.02 | 0.01 | 0.01 | 7.61 |
| AK100477 | 0.00 | 6.23 | E | 0.26 | 0.21 | 0.39 | 0.49 | 3.05 |
| AK110880 | 0.00 | 15.42 | E | 0.16 | 0.14 | 0.11 | 0.36 | 5.53 |
| AK111012 | 0.00 | 7.46 | E | 0.14 | 0.18 | 0.10 | 0.65 | 4.87 |
| AK103242 | 0.00 | 2.41 | D | 0.15 | 0.19 | 1.64 | 3.95 | 0.69 |
| AK101304 | 0.00 | 1.78 | D | 0.14 | 0.38 | 1.53 | 2.71 | 1.52 |
| AK072705 | 0.00 | 12.65 | E | 0.07 | 0.04 | 0.21 | 0.48 | 6.04 |
| AK060688 | 0.00 | 8.26 | E | 0.29 | 0.26 | 0.21 | 0.96 | 7.90 |
| AK109701 | 0.00 | 2.16 | C | 0.09 | 0.12 | 3.04 | 1.02 | 1.41 |
| AK059688 | 0.00 | 3.50 | E | 0.75 | 0.59 | 0.44 | 0.48 | 2.63 |
| AK071874 | 0.00 | 5.40 | E | 0.32 | 0.45 | 0.60 | 1.43 | 7.73 |
| AK068432 | 0.00 | 1.58 | B | 1.55 | 2.44 | 0.32 | 0.27 | 0.21 |
| AK066957 | 0.00 | 7.99 | E | 0.32 | 0.29 | 0.17 | 0.53 | 4.22 |
| AK109532 | 0.00 | 2.67 | E | 0.17 | 0.28 | 0.21 | 1.05 | 2.81 |
| AK059891 | 0.00 | 1.55 | D | 1.24 | 1.26 | 0.25 | 1.94 | 0.39 |
| AK072680 | 0.00 | 1.50 | E | 0.54 | 0.27 | 0.70 | 1.77 | 2.66 |
| AK065824 | 0.00 | 11.49 | E | 0.06 | 0.09 | 0.03 | 0.35 | 3.98 |
| AK103903 | 0.00 | 6.42 | E | 0.21 | 0.27 | 0.17 | 0.66 | 4.25 |
| AK105319 | 0.00 | 4.60 | D | 0.45 | 0.56 | 0.70 | 3.20 | 0.47 |
| AK111236 | 0.00 | 6.45 | E | 0.28 | 0.46 | 0.21 | 0.32 | 2.99 |
| AK066208 | 0.00 | 1.79 | B | 1.51 | 2.71 | 0.12 | 0.12 | 0.18 |
| AK065638 | 0.00 | 8.38 | E | 0.43 | 0.27 | 0.21 | 0.33 | 3.63 |
| AK110675 | 0.00 | 4.35 | E | 0.07 | 0.09 | 0.94 | 0.89 | 4.09 |
| AK102697 | 0.00 | 3.53 | C | 1.42 | 1.37 | 5.00 | 0.79 | 0.43 |
| AK106800 | 0.00 | 1.89 | E | 1.36 | 0.49 | 0.12 | 0.18 | 2.57 |
| AK062918 | 0.00 | 1.63 | B | 1.77 | 2.88 | 0.58 | 0.70 | 0.54 |
| AK068336 | 0.00 | 5.44 | C | 0.25 | 0.21 | 4.41 | 0.54 | 0.81 |
| AK068516 | 0.00 | 1.50 | E | 0.54 | 0.30 | 1.41 | 0.87 | 2.13 |
| AK073881 | 0.00 | 12.00 | E | 0.31 | 0.23 | 0.25 | 0.83 | 9.98 |
| AK111868 | 0.00 | 7.46 | E | 0.33 | 0.22 | 0.37 | 0.32 | 2.80 |
| AK100314 | 0.00 | 2.02 | D | 0.45 | 0.55 | 0.70 | 2.30 | 1.14 |
| AK071006 | 0.00 | 1.82 | B | 1.55 | 2.83 | 0.31 | 0.25 | 0.16 |
| AK099867 | 0.00 | 1.69 | C | 1.48 | 1.02 | 2.49 | 0.23 | 0.14 |
| AK101209 | 0.00 | 3.32 | D | 0.31 | 0.42 | 0.40 | 2.91 | 0.88 |
| AK071558 | 0.00 | 8.05 | E | 0.29 | 0.27 | 0.41 | 0.43 | 3.44 |
| AK109943 | 0.00 | 3.71 | E | 0.08 | 0.08 | 1.17 | 0.99 | 4.34 |
| AK108806 | 0.00 | 5.66 | C | 0.84 | 0.88 | 4.97 | 0.39 | 0.30 |
| AK059864 | 0.00 | 1.61 | D | 0.36 | 0.24 | 0.94 | 2.51 | 1.55 |
| AK072350 | 0.00 | 5.61 | E | 0.03 | 0.12 | 0.45 | 0.70 | 3.95 |
| AK108512 | 0.00 | 2.12 | C | 0.38 | 0.23 | 3.07 | 1.40 | 1.45 |
| AK064911 | 0.00 | 2.81 | E | 0.13 | 0.17 | 0.43 | 0.97 | 2.73 |
| AK100638 | 0.00 | 1.81 | C | 1.02 | 1.29 | 2.34 | 0.52 | 0.34 |
| AK102181 | 0.00 | 4.63 | E | 0.66 | 0.43 | 0.28 | 0.47 | 3.05 |
| AK070857 | 0.00 | 1.62 | C | 1.16 | 1.26 | 2.04 | 0.48 | 0.30 |
| AK105170 | 0.00 | 1.96 | C | 1.37 | 0.76 | 2.69 | 0.25 | 0.27 |
| AK071914 | 0.00 | 5.49 | E | 0.42 | 0.42 | 0.58 | 0.52 | 3.16 |
| AK100944 | 0.00 | 5.88 | E | 0.21 | 0.29 | 0.19 | 0.69 | 4.03 |
| AK058331 | 0.00 | 1.52 | D | 0.21 | 0.40 | 1.87 | 2.83 | 0.82 |
| AK100685 | 0.00 | 1.76 | D | 0.31 | 0.33 | 0.83 | 2.44 | 1.39 |
| AK099963 | 0.00 | 11.45 | E | 0.28 | 0.28 | 0.21 | 0.73 | 8.37 |
| AK111051 | 0.00 | 6.61 | E | 0.43 | 0.41 | 0.29 | 0.47 | 3.12 |
| AK063422 | 0.00 | 12.23 | E | 0.35 | 0.31 | 0.23 | 0.88 | 10.78 |
| AK111351 | 0.00 | 9.16 | E | 0.15 | 0.13 | 0.17 | 0.40 | 3.68 |
| AK062801 | 0.00 | 1.92 | D | 0.36 | 0.54 | 1.48 | 2.84 | 1.27 |
| AK108397 | 0.00 | 4.96 | E | 0.21 | 0.28 | 0.16 | 0.82 | 4.06 |
| AK102113 | 0.00 | 2.34 | D | 0.57 | 0.83 | 0.97 | 2.27 | 0.35 |
| AK062634 | 0.00 | 2.83 | E | 0.23 | 0.32 | 0.55 | 1.27 | 3.60 |
| AK067526 | 0.00 | 2.46 | D | 0.47 | 0.45 | 0.50 | 2.74 | 1.11 |
| AK110824 | 0.00 | 3.10 | D | 0.29 | 0.24 | 0.22 | 3.58 | 1.15 |
| AK059130 | 0.00 | 1.53 | C | 1.08 | 1.33 | 2.04 | 0.47 | 0.34 |
| AK104993 | 0.00 | 5.59 | E | 0.61 | 0.51 | 0.63 | 0.51 | 3.55 |
| AK110871 | 0.00 | 2.01 | B | 1.12 | 2.24 | 0.36 | 0.39 | 0.33 |
| AK107510 | 0.00 | 1.55 | D | 0.48 | 0.27 | 0.29 | 2.52 | 1.63 |
| AK067991 | 0.00 | 7.78 | E | 0.16 | 0.19 | 0.14 | 0.92 | 7.18 |
| AK099234 | 0.00 | 1.60 | C | 1.02 | 1.34 | 2.14 | 0.42 | 0.36 |
| AK061068 | 0.00 | 1.58 | E | 0.36 | 0.58 | 0.97 | 1.65 | 2.62 |
| AK060943 | 0.00 | 1.52 | B | 1.60 | 2.43 | 0.41 | 0.49 | 0.41 |
| AK066175 | 0.00 | 5.73 | E | 0.22 | 0.25 | 0.22 | 0.71 | 4.06 |
| AK107902 | 0.00 | 5.76 | E | 0.44 | 0.25 | 0.20 | 0.50 | 2.86 |
| AK101509 | 0.00 | 1.65 | C | 1.08 | 1.38 | 2.27 | 0.48 | 0.34 |
| AK068163 | 0.00 | 1.89 | C | 1.54 | 1.43 | 2.92 | 0.43 | 0.53 |
| AK072782 | 0.00 | 5.58 | E | 0.22 | 0.27 | 0.18 | 0.71 | 3.95 |
| AK108015 | 0.00 | 3.23 | E | 0.42 | 0.71 | 0.53 | 0.66 | 2.31 |
| AK070903 | 0.00 | 1.64 | D | 0.32 | 0.25 | 0.87 | 2.57 | 1.57 |
| AK065770 | 0.00 | 1.78 | C | 0.40 | 0.35 | 2.74 | 1.13 | 1.54 |
| AK067128 | 0.00 | 2.72 | C | 1.74 | 1.37 | 4.73 | 0.91 | 0.49 |
| AK069092 | 0.00 | 2.91 | C | 0.30 | 0.39 | 3.94 | 1.35 | 0.94 |
| AK073888 | 0.00 | 4.07 | E | 0.69 | 0.55 | 0.47 | 0.28 | 2.79 |
| AK065299 | 0.00 | 1.79 | A | 3.35 | 1.87 | 0.63 | 0.39 | 0.17 |
| AK103139 | 0.00 | 5.63 | E | 0.78 | 0.49 | 0.48 | 0.37 | 4.37 |
| AK060291 | 0.00 | 3.45 | E | 0.66 | 0.80 | 0.73 | 0.77 | 2.76 |
| AK107750 | 0.00 | 2.98 | E | 0.42 | 0.47 | 0.59 | 0.95 | 2.84 |
| AK108524 | 0.00 | 9.79 | C | 0.09 | 0.15 | 4.94 | 0.26 | 0.50 |
| AK058767 | 0.00 | 1.51 | C | 1.03 | 1.32 | 1.99 | 0.55 | 0.33 |
| AK111303 | 0.00 | 1.59 | A | 1.87 | 1.15 | 1.17 | 0.41 | 0.89 |
| AK107086 | 0.00 | 2.38 | C | 0.47 | 0.25 | 3.92 | 1.65 | 0.92 |
| AK109473 | 0.00 | 1.74 | B | 1.41 | 2.44 | 0.30 | 0.26 | 0.25 |
| AK065797 | 0.00 | 3.87 | E | 0.31 | 0.62 | 0.36 | 0.42 | 2.39 |
| AK071859 | 0.00 | 1.62 | D | 0.26 | 0.38 | 0.81 | 2.83 | 1.75 |
| AK108770 | 0.00 | 1.55 | C | 1.31 | 1.29 | 2.04 | 0.51 | 0.37 |
| AK059972 | 0.00 | 7.40 | E | 0.46 | 0.29 | 0.25 | 0.43 | 3.37 |
| AK062810 | 0.00 | 1.65 | C | 1.18 | 1.31 | 2.17 | 0.56 | 0.34 |
| AK107560 | 0.00 | 6.24 | E | 0.23 | 0.25 | 0.35 | 0.83 | 5.19 |
| AK071000 | 0.00 | 9.91 | E | 0.34 | 0.26 | 0.23 | 0.90 | 8.94 |
| AK059965 | 0.00 | 1.51 | B | 1.45 | 2.19 | 0.44 | 0.41 | 0.39 |
| AK058220 | 0.00 | 2.69 | E | 0.85 | 0.57 | 0.48 | 0.28 | 2.28 |
| AK068803 | 0.00 | 2.54 | A | 3.40 | 0.92 | 1.34 | 0.56 | 0.59 |
| AK106378 | 0.00 | 5.19 | C | 0.78 | 0.77 | 4.03 | 0.57 | 0.52 |
| AK072728 | 0.00 | 1.55 | D | 0.64 | 0.73 | 0.97 | 1.81 | 1.17 |
| AK108786 | 0.00 | 2.51 | D | 0.34 | 0.30 | 1.15 | 4.40 | 1.75 |
| AK110269 | 0.00 | 4.39 | E | 0.19 | 0.21 | 0.29 | 0.86 | 3.78 |
| AK100940 | 0.00 | 4.05 | E | 0.60 | 0.75 | 0.92 | 0.61 | 3.72 |
| AK068331 | 0.00 | 7.25 | E | 0.37 | 0.30 | 0.28 | 0.62 | 4.49 |
| AK068661 | 0.00 | 1.66 | C | 1.05 | 1.39 | 2.32 | 0.48 | 0.35 |
| AK102087 | 0.00 | 1.83 | E | 0.25 | 0.16 | 0.70 | 1.52 | 2.78 |
| AK071034 | 0.00 | 5.92 | E | 0.45 | 0.42 | 0.39 | 0.27 | 2.65 |
| AK100299 | 0.00 | 1.79 | C | 1.12 | 1.30 | 2.34 | 0.51 | 0.39 |
| AK061250 | 0.00 | 3.55 | D | 0.53 | 0.43 | 0.30 | 3.26 | 0.92 |
| AK100448 | 0.00 | 1.55 | C | 1.14 | 0.54 | 1.77 | 0.68 | 1.05 |
| AK058385 | 0.00 | 11.50 | E | 0.62 | 0.29 | 0.28 | 0.62 | 7.14 |
| AK073278 | 0.00 | 2.81 | E | 0.82 | 0.54 | 0.69 | 0.17 | 2.29 |
| AK064122 | 0.00 | 2.07 | E | 0.50 | 0.54 | 0.59 | 1.22 | 2.52 |
| AK058603 | 0.00 | 2.66 | E | 0.33 | 0.33 | 0.55 | 0.90 | 2.39 |
| AK102518 | 0.00 | 1.52 | C | 1.00 | 1.35 | 2.04 | 0.50 | 0.33 |
| AK060496 | 0.00 | 1.51 | C | 0.46 | 0.41 | 5.55 | 0.77 | 3.69 |
| AK109580 | 0.00 | 1.52 | C | 1.38 | 1.31 | 2.10 | 0.47 | 0.35 |
| AK106427 | 0.00 | 15.66 | E | 0.08 | 0.18 | 0.07 | 0.64 | 10.00 |
| AK072062 | 0.00 | 1.52 | A | 2.29 | 1.50 | 0.78 | 0.76 | 0.28 |
| AK067485 | 0.00 | 4.30 | E | 0.14 | 0.09 | 0.73 | 0.46 | 3.12 |
| AK069464 | 0.00 | 2.97 | D | 0.69 | 0.38 | 0.58 | 3.03 | 1.02 |
| AK110703 | 0.00 | 2.96 | D | 0.25 | 0.47 | 0.87 | 2.58 | 0.85 |
| AK065598 | 0.00 | 2.19 | E | 0.30 | 0.40 | 0.45 | 1.05 | 2.29 |
| AK071565 | 0.00 | 2.01 | E | 0.33 | 0.25 | 1.23 | 1.28 | 2.58 |
| AK107338 | 0.00 | 2.54 | D | 0.44 | 0.55 | 0.70 | 2.08 | 0.82 |
| AK059469 | 0.00 | 3.08 | C | 0.44 | 0.24 | 3.28 | 0.98 | 1.06 |
| AK059969 | 0.00 | 4.17 | E | 0.32 | 0.29 | 0.26 | 0.68 | 2.84 |
| AK070643 | 0.00 | 2.42 | D | 0.14 | 0.31 | 1.96 | 4.76 | 0.37 |
| AK106309 | 0.00 | 1.68 | D | 0.18 | 0.27 | 0.32 | 1.99 | 1.18 |
| AK104720 | 0.00 | 1.77 | E | 0.29 | 0.14 | 1.43 | 1.60 | 2.83 |
| AK069746 | 0.00 | 4.10 | E | 0.20 | 0.27 | 0.27 | 0.96 | 3.94 |
| AK061806 | 0.00 | 2.20 | D | 0.26 | 0.41 | 0.58 | 2.57 | 1.17 |
| AK071546 | 0.00 | 7.55 | E | 0.39 | 0.25 | 0.16 | 0.28 | 2.92 |
| AK062879 | 0.00 | 1.50 | E | 0.70 | 0.77 | 0.53 | 1.04 | 1.56 |
| AK110892 | 0.00 | 1.67 | C | 0.44 | 1.42 | 2.90 | 1.74 | 1.58 |
| AK066341 | 0.00 | 4.20 | E | 0.41 | 0.50 | 0.22 | 0.98 | 4.09 |
| AK111391 | 0.00 | 1.61 | C | 1.06 | 1.37 | 2.20 | 0.53 | 0.34 |
| AK068261 | 0.00 | 3.12 | E | 0.20 | 0.23 | 0.08 | 0.95 | 2.97 |
| AK065460 | 0.00 | 2.19 | C | 0.65 | 0.58 | 2.63 | 1.20 | 0.59 |
| AK068857 | 0.00 | 6.24 | E | 0.34 | 0.33 | 0.46 | 0.46 | 2.87 |
| AK067007 | 0.00 | 2.11 | C | 1.19 | 1.31 | 2.77 | 0.65 | 0.64 |
| AK072777 | 0.00 | 1.70 | E | 0.24 | 0.15 | 1.66 | 1.51 | 2.81 |
| AK108713 | 0.00 | 1.56 | C | 0.39 | 0.54 | 1.91 | 1.22 | 0.81 |
| AK102845 | 0.00 | 1.82 | D | 0.57 | 0.40 | 1.24 | 2.26 | 1.02 |
| AK070763 | 0.00 | 1.73 | D | 0.42 | 0.54 | 0.62 | 1.61 | 0.93 |
| AK058892 | 0.00 | 6.12 | E | 0.23 | 0.21 | 0.20 | 0.46 | 2.80 |
| AK106457 | 0.00 | 2.79 | E | 0.82 | 0.79 | 0.31 | 0.50 | 2.28 |
| AK067282 | 0.00 | 2.24 | C | 0.41 | 0.47 | 3.15 | 1.41 | 0.80 |
| AK065137 | 0.00 | 4.88 | A | 4.21 | 0.62 | 0.59 | 0.86 | 0.25 |
| AK101504 | 0.00 | 1.68 | D | 0.50 | 0.59 | 0.63 | 1.59 | 0.95 |
| AK109196 | 0.00 | 4.62 | C | 0.69 | 0.66 | 3.18 | 0.52 | 0.49 |
| AK101097 | 0.00 | 1.98 | E | 0.49 | 0.42 | 1.31 | 1.05 | 2.59 |
| AK070032 | 0.00 | 1.67 | D | 1.12 | 1.16 | 0.40 | 1.93 | 0.74 |
| AK102648 | 0.00 | 2.83 | D | 0.37 | 0.48 | 0.60 | 3.25 | 1.15 |
| AK103715 | 0.00 | 1.70 | C | 1.01 | 1.30 | 2.21 | 0.47 | 0.35 |
| AK100587 | 0.00 | 2.58 | D | 0.44 | 0.42 | 0.67 | 3.01 | 1.16 |
| AK107040 | 0.00 | 1.83 | B | 0.76 | 2.22 | 0.60 | 0.55 | 1.21 |
| AK071527 | 0.00 | 2.15 | E | 0.23 | 0.16 | 1.51 | 1.54 | 3.31 |
| AK064207 | 0.00 | 2.93 | D | 0.53 | 0.24 | 0.21 | 2.84 | 0.97 |
| AK100008 | 0.00 | 2.97 | E | 0.61 | 0.64 | 0.77 | 0.95 | 2.81 |
| AK070013 | 0.00 | 3.09 | C | 0.90 | 0.46 | 3.41 | 1.10 | 0.35 |
| AK066728 | 0.00 | 1.51 | E | 0.50 | 0.37 | 0.66 | 0.86 | 1.29 |
| AK105244 | 0.00 | 1.71 | D | 0.59 | 0.35 | 1.67 | 4.14 | 2.41 |
| AK108828 | 0.00 | 2.34 | E | 0.80 | 0.91 | 0.39 | 0.66 | 2.12 |
| AK072890 | 0.00 | 2.37 | C | 1.10 | 1.48 | 3.52 | 0.88 | 0.64 |
| AK071590 | 0.00 | 2.93 | E | 1.25 | 0.85 | 0.48 | 0.86 | 3.65 |
| AK073402 | 0.00 | 2.66 | E | 0.53 | 0.52 | 0.53 | 0.93 | 2.48 |
| AK069249 | 0.00 | 2.15 | E | 0.23 | 0.16 | 1.51 | 1.44 | 3.23 |
| AK062570 | 0.00 | 2.19 | E | 0.38 | 0.31 | 0.73 | 1.33 | 2.91 |
| AK058320 | 0.00 | 1.65 | A | 1.91 | 1.16 | 0.42 | 0.50 | 0.44 |
| AK100550 | 0.00 | 6.42 | E | 0.04 | 0.10 | 0.25 | 0.69 | 4.44 |
| AK070795 | 0.00 | 2.03 | D | 0.84 | 1.34 | 1.11 | 2.72 | 0.18 |
| AK071118 | 0.00 | 1.82 | D | 0.38 | 0.53 | 1.15 | 2.40 | 1.32 |
| AK064010 | 0.00 | 1.58 | A | 2.45 | 1.54 | 0.06 | 0.97 | 0.11 |
| AK106807 | 0.00 | 2.65 | E | 0.28 | 0.33 | 0.79 | 0.84 | 2.22 |
| AK069992 | 0.00 | 5.82 | D | 0.53 | 0.39 | 0.82 | 4.78 | 0.05 |
| AK105248 | 0.00 | 1.77 | E | 0.28 | 0.16 | 1.41 | 1.48 | 2.62 |
| AK073394 | 0.00 | 2.65 | E | 0.57 | 0.82 | 0.40 | 0.90 | 2.37 |
| AK059143 | 0.00 | 1.64 | E | 1.12 | 0.86 | 0.47 | 1.40 | 2.29 |
| AK072876 | 0.00 | 1.59 | E | 0.24 | 0.45 | 0.37 | 1.49 | 2.37 |
| AK058804 | 0.00 | 1.56 | A | 2.04 | 1.30 | 1.19 | 0.38 | 0.52 |
| AK069027 | 0.00 | 2.53 | E | 0.53 | 0.53 | 0.38 | 1.00 | 2.53 |
| AK068540 | 0.00 | 2.14 | C | 0.47 | 0.62 | 2.48 | 1.16 | 0.50 |
| AK060204 | 0.00 | 1.95 | D | 0.42 | 0.71 | 0.98 | 2.00 | 1.03 |
| AK072532 | 0.00 | 3.27 | E | 0.35 | 0.44 | 0.71 | 0.55 | 2.32 |
| AK064274 | 0.00 | 3.58 | E | 0.20 | 0.25 | 0.28 | 1.01 | 3.63 |
| AK099305 | 0.00 | 1.74 | E | 0.32 | 0.29 | 0.39 | 1.03 | 1.80 |
| AK058540 | 0.00 | 1.54 | C | 0.22 | 0.47 | 1.86 | 1.16 | 1.21 |
| AK109002 | 0.00 | 1.54 | C | 0.98 | 1.61 | 2.47 | 0.54 | 0.48 |
| AK071075 | 0.00 | 1.74 | E | 0.67 | 0.60 | 0.94 | 1.17 | 2.04 |
| AK103837 | 0.00 | 1.56 | E | 0.84 | 1.70 | 0.46 | 0.71 | 2.64 |
| AK066992 | 0.00 | 3.56 | E | 0.45 | 0.72 | 0.55 | 0.88 | 3.15 |
| AK101925 | 0.00 | 5.13 | E | 0.82 | 0.58 | 0.50 | 0.79 | 4.21 |
| AK105366 | 0.00 | 1.50 | D | 1.65 | 0.98 | 0.30 | 2.48 | 0.34 |
| AK111498 | 0.00 | 1.59 | E | 0.25 | 0.39 | 0.40 | 1.43 | 2.28 |
| AK108715 | 0.00 | 1.70 | C | 0.96 | 1.10 | 2.03 | 0.42 | 1.20 |
| AK111028 | 0.00 | 4.43 | C | 0.83 | 0.79 | 3.66 | 0.56 | 0.50 |
| AK105549 | 0.00 | 1.51 | D | 1.09 | 1.17 | 0.25 | 1.77 | 0.56 |
| AK103539 | 0.00 | 6.14 | E | 0.36 | 0.38 | 0.40 | 0.74 | 4.56 |
| AK072534 | 0.00 | 2.12 | D | 1.70 | 0.82 | 0.30 | 3.61 | 0.44 |
| AK100513 | 0.00 | 1.94 | E | 0.25 | 0.37 | 0.47 | 1.22 | 2.37 |
| AK060302 | 0.00 | 3.77 | E | 0.93 | 0.76 | 0.60 | 0.30 | 3.50 |
| AK065686 | 0.00 | 2.21 | D | 0.64 | 0.37 | 1.23 | 2.72 | 1.06 |
| AK063303 | 0.00 | 1.73 | D | 0.90 | 1.33 | 1.06 | 2.29 | 0.16 |
| AK073497 | 0.00 | 2.06 | D | 0.83 | 0.74 | 1.25 | 2.58 | 0.58 |
| AK063068 | 0.00 | 1.54 | E | 0.50 | 0.54 | 1.46 | 0.90 | 2.25 |
| AK108125 | 0.00 | 10.44 | E | 0.28 | 0.28 | 0.23 | 0.62 | 6.42 |
| AK064901 | 0.00 | 4.52 | C | 0.71 | 0.65 | 3.19 | 0.52 | 0.42 |
| AK106596 | 0.00 | 2.32 | D | 0.46 | 0.58 | 0.70 | 2.02 | 0.87 |
| AK073574 | 0.00 | 4.00 | E | 0.49 | 0.36 | 0.70 | 0.75 | 3.00 |
| AK065696 | 0.00 | 2.93 | E | 0.39 | 0.39 | 0.56 | 0.79 | 2.32 |
| AK071598 | 0.00 | 2.07 | D | 0.95 | 0.93 | 0.68 | 1.98 | 0.70 |
| AK060218 | 0.00 | 3.57 | E | 0.80 | 0.56 | 0.63 | 0.46 | 2.85 |
| AK111988 | 0.00 | 1.79 | D | 0.35 | 0.50 | 0.81 | 1.80 | 1.01 |
| AK106334 | 0.00 | 1.67 | D | 0.20 | 0.35 | 0.52 | 2.46 | 1.47 |
| AK060305 | 0.00 | 1.89 | D | 0.89 | 0.69 | 0.68 | 2.43 | 1.28 |
| AK064687 | 0.00 | 1.59 | D | 0.87 | 0.63 | 1.66 | 2.63 | 0.76 |
| AK099628 | 0.00 | 1.57 | E | 0.44 | 0.47 | 0.65 | 1.25 | 1.96 |
| AK103687 | 0.00 | 4.55 | C | 0.76 | 0.65 | 3.44 | 0.52 | 0.50 |
| AK102049 | 0.00 | 2.10 | E | 0.34 | 0.38 | 0.97 | 0.24 | 2.03 |
| AK067992 | 0.00 | 1.58 | E | 0.40 | 0.45 | 0.96 | 1.43 | 2.26 |
| AK067754 | 0.00 | 1.80 | D | 0.60 | 0.60 | 1.18 | 2.44 | 1.36 |
| AK073365 | 0.00 | 1.86 | D | 0.51 | 0.50 | 0.73 | 2.93 | 1.57 |
| AK073991 | 0.00 | 1.53 | B | 1.42 | 2.18 | 0.82 | 1.15 | 0.64 |
| AK065838 | 0.00 | 2.87 | D | 0.68 | 1.04 | 0.40 | 2.99 | 0.78 |
| AK109649 | 0.00 | 3.21 | E | 0.57 | 0.72 | 0.57 | 0.55 | 2.31 |
| AK060668 | 0.00 | 3.14 | C | 0.80 | 0.71 | 3.29 | 1.05 | 0.73 |
| AK063406 | 0.00 | 3.49 | C | 0.78 | 0.51 | 3.32 | 0.95 | 0.78 |
| AK062628 | 0.00 | 1.68 | D | 1.04 | 1.08 | 0.39 | 2.55 | 1.52 |
| AK109320 | 0.00 | 5.87 | E | 0.53 | 0.46 | 0.39 | 0.83 | 4.90 |
| AK072730 | 0.00 | 3.66 | D | 0.44 | 0.46 | 0.31 | 2.96 | 0.81 |
| AK102204 | 0.00 | 2.23 | C | 1.33 | 0.88 | 2.96 | 0.88 | 0.63 |
| AK064688 | 0.00 | 2.94 | E | 0.86 | 0.83 | 0.93 | 0.84 | 2.73 |
| AK063762 | 0.00 | 1.51 | B | 1.22 | 1.85 | 0.76 | 0.90 | 0.43 |
| AK109394 | 0.00 | 1.68 | D | 0.16 | 0.57 | 1.81 | 3.04 | 0.59 |
| AK063934 | 0.00 | 4.63 | E | 0.23 | 0.29 | 0.32 | 0.69 | 3.20 |
| AK101185 | 0.00 | 1.75 | D | 1.09 | 1.11 | 0.58 | 1.95 | 0.97 |
| AK062751 | 0.00 | 1.80 | E | 0.94 | 0.90 | 0.70 | 0.47 | 1.70 |
| AK063435 | 0.00 | 3.05 | E | 0.29 | 0.44 | 1.04 | 1.12 | 3.42 |
| AK100910 | 0.00 | 2.12 | D | 1.04 | 1.45 | 0.28 | 3.07 | 0.84 |
| AK060457 | 0.00 | 1.65 | B | 1.85 | 3.06 | 0.02 | 0.04 | 0.12 |
| AK065424 | 0.00 | 2.39 | D | 0.82 | 1.21 | 0.93 | 2.91 | 0.80 |
| AK070840 | 0.00 | 2.07 | E | 0.93 | 0.89 | 0.72 | 0.46 | 1.92 |
| AK066003 | 0.00 | 3.14 | D | 0.48 | 0.37 | 0.87 | 2.74 | 0.85 |
| AK061094 | 0.00 | 1.63 | D | 0.94 | 0.47 | 0.21 | 2.24 | 1.37 |
| AK103787 | 0.00 | 1.93 | D | 0.60 | 0.51 | 0.58 | 2.44 | 1.26 |
| AK102912 | 0.00 | 3.63 | E | 0.52 | 0.57 | 0.48 | 0.57 | 2.06 |
| AK109120 | 0.00 | 4.56 | E | 0.02 | 0.08 | 0.09 | 0.94 | 4.30 |
| AK071791 | 0.00 | 3.12 | E | 0.36 | 0.50 | 0.57 | 0.85 | 2.66 |
| AK071927 | 0.00 | 1.59 | A | 2.09 | 1.32 | 1.09 | 0.33 | 0.52 |
| AK073203 | 0.00 | 1.75 | A | 3.22 | 1.84 | 0.01 | 0.12 | 0.02 |
| AK108287 | 0.00 | 1.77 | D | 0.59 | 0.41 | 1.22 | 2.17 | 0.96 |
| AK070057 | 0.00 | 1.58 | D | 0.65 | 0.42 | 1.33 | 2.10 | 0.91 |
| AK101105 | 0.00 | 3.13 | E | 0.79 | 0.84 | 0.28 | 0.50 | 2.63 |
| AK058766 | 0.00 | 1.73 | D | 0.69 | 0.55 | 0.60 | 3.60 | 2.08 |
| AK111062 | 0.00 | 1.80 | B | 0.91 | 1.76 | 0.97 | 0.84 | 0.60 |
| AK106130 | 0.00 | 7.59 | E | 0.47 | 0.50 | 0.37 | 0.52 | 3.91 |
| AK107184 | 0.00 | 2.82 | E | 1.51 | 1.06 | 1.70 | 0.80 | 4.79 |
| AK073406 | 0.00 | 1.77 | E | 0.70 | 0.76 | 0.76 | 1.14 | 2.02 |
| AK106375 | 0.00 | 1.51 | C | 0.46 | 0.55 | 1.81 | 1.20 | 0.87 |
| AK111437 | 0.00 | 5.50 | D | 0.56 | 0.30 | 0.87 | 7.29 | 1.33 |
| AK066987 | 0.00 | 2.40 | D | 0.84 | 1.33 | 0.31 | 3.18 | 0.90 |
| AK071181 | 0.00 | 1.81 | D | 0.85 | 0.67 | 1.38 | 2.49 | 0.81 |
| AK101099 | 0.00 | 1.64 | C | 0.45 | 0.46 | 2.08 | 1.27 | 1.15 |
| AK062954 | 0.00 | 1.59 | A | 2.00 | 1.12 | 0.10 | 1.26 | 0.25 |
| AK064432 | 0.00 | 5.12 | E | 0.58 | 0.44 | 0.38 | 0.60 | 3.08 |
| AK110482 | 0.00 | 1.91 | E | 1.51 | 0.99 | 0.74 | 0.40 | 2.88 |
| AK103158 | 0.00 | 6.06 | E | 0.22 | 0.35 | 0.28 | 0.58 | 3.49 |
| AK105584 | 0.00 | 4.46 | D | 0.53 | 0.43 | 0.36 | 4.21 | 0.94 |
| AK103122 | 0.00 | 1.62 | C | 0.75 | 0.50 | 2.45 | 1.51 | 0.80 |
| AK064752 | 0.00 | 1.75 | B | 1.18 | 2.10 | 0.89 | 1.20 | 0.60 |
| AK059647 | 0.00 | 1.85 | D | 0.72 | 0.70 | 0.55 | 2.16 | 1.17 |
| AK106534 | 0.00 | 1.67 | E | 0.19 | 0.43 | 0.86 | 1.19 | 1.99 |
| AK065412 | 0.00 | 2.00 | C | 1.26 | 0.71 | 2.52 | 0.29 | 0.36 |
| AK067223 | 0.00 | 4.08 | E | 0.67 | 0.49 | 0.69 | 0.77 | 3.15 |
| AK110966 | 0.00 | 2.05 | E | 0.55 | 0.48 | 0.54 | 1.15 | 2.37 |
| AK066324 | 0.00 | 1.50 | E | 0.53 | 0.55 | 0.91 | 1.05 | 1.58 |
| AK106834 | 0.00 | 1.89 | D | 0.72 | 0.86 | 0.40 | 2.24 | 1.18 |
| AK064230 | 0.00 | 2.30 | E | 0.59 | 0.83 | 0.35 | 0.72 | 1.92 |
| AK058342 | 0.00 | 3.73 | D | 0.56 | 0.47 | 0.34 | 2.91 | 0.78 |
| AK105594 | 0.00 | 1.91 | D | 0.56 | 0.59 | 0.86 | 1.95 | 1.02 |
| AK110053 | 0.00 | 2.44 | C | 0.34 | 0.35 | 2.83 | 1.16 | 0.79 |
| AK073452 | 0.00 | 1.67 | E | 0.43 | 0.90 | 1.03 | 1.23 | 2.05 |
| AK064677 | 0.00 | 1.95 | D | 1.52 | 0.91 | 1.38 | 2.96 | 0.53 |
| AK072355 | 0.00 | 1.58 | D | 0.54 | 0.46 | 1.15 | 2.86 | 1.81 |
| AK101462 | 0.00 | 2.81 | E | 0.23 | 0.30 | 0.12 | 0.96 | 2.69 |
| AK071489 | 0.00 | 3.18 | D | 0.51 | 0.52 | 0.55 | 3.81 | 1.20 |
| AK069270 | 0.00 | 1.59 | E | 1.74 | 0.95 | 0.83 | 0.45 | 2.77 |
| AK066607 | 0.00 | 1.54 | D | 0.73 | 0.92 | 1.64 | 2.53 | 0.85 |
| AK067112 | 0.00 | 1.53 | D | 0.73 | 0.49 | 1.26 | 1.94 | 1.09 |
| AK100913 | 0.00 | 1.53 | D | 0.60 | 0.67 | 1.50 | 2.30 | 0.70 |
| AK064254 | 0.00 | 2.12 | E | 0.50 | 0.66 | 0.75 | 0.85 | 1.80 |
| AK064547 | 0.00 | 1.61 | D | 0.62 | 0.69 | 0.87 | 1.40 | 0.83 |
| AK062659 | 0.00 | 4.75 | E | 0.73 | 0.69 | 0.91 | 0.69 | 4.34 |
| AK058313 | 0.00 | 4.73 | E | 0.33 | 0.56 | 0.24 | 0.72 | 3.39 |
| AK072652 | 0.00 | 1.63 | E | 0.92 | 1.10 | 1.18 | 0.80 | 1.92 |
| AK107291 | 0.00 | 2.41 | E | 0.86 | 0.82 | 0.76 | 0.75 | 2.07 |
| AK100709 | 0.00 | 1.54 | D | 0.46 | 0.47 | 1.66 | 2.55 | 1.55 |
| AK070653 | 0.00 | 1.86 | C | 0.21 | 0.35 | 2.39 | 1.29 | 1.26 |
| AK071855 | 0.00 | 2.57 | E | 1.06 | 0.88 | 0.50 | 0.97 | 2.72 |
| AK070341 | 0.00 | 1.75 | A | 2.09 | 1.19 | 1.05 | 0.84 | 0.24 |
| AK067080 | 0.00 | 1.60 | B | 1.11 | 1.99 | 1.08 | 1.24 | 0.43 |
| AK099471 | 0.00 | 1.76 | A | 3.81 | 2.17 | 1.79 | 0.31 | 0.19 |
| AK102308 | 0.00 | 1.51 | E | 1.14 | 1.08 | 0.91 | 0.47 | 1.73 |
| AK066134 | 0.00 | 1.62 | A | 2.47 | 1.52 | 1.26 | 0.45 | 0.29 |
| AK106448 | 0.00 | 2.19 | D | 0.16 | 0.25 | 0.49 | 2.13 | 0.97 |
| AK108509 | 0.00 | 1.51 | D | 0.84 | 0.51 | 0.67 | 1.74 | 1.15 |
| AK068500 | 0.00 | 2.14 | D | 0.43 | 0.67 | 1.09 | 3.86 | 1.81 |
| AK064433 | 0.00 | 1.51 | D | 0.69 | 0.65 | 1.09 | 1.68 | 1.11 |
| AK107473 | 0.00 | 1.99 | C | 0.94 | 0.73 | 2.42 | 1.21 | 0.68 |
| AK099811 | 0.00 | 1.93 | D | 0.71 | 0.61 | 1.46 | 3.08 | 1.60 |
| AK060930 | 0.00 | 1.64 | D | 0.68 | 0.58 | 1.19 | 1.95 | 0.57 |
| AK105195 | 0.00 | 1.69 | D | 0.52 | 0.51 | 0.99 | 2.00 | 1.18 |
| AK063358 | 0.00 | 1.67 | E | 0.31 | 0.47 | 1.44 | 1.77 | 2.94 |
| AK070539 | 0.00 | 1.93 | B | 0.77 | 1.63 | 0.85 | 0.84 | 0.56 |
| AK108671 | 0.00 | 2.51 | C | 0.86 | 0.71 | 2.15 | 0.58 | 0.82 |
| AK101646 | 0.00 | 2.03 | D | 0.93 | 0.98 | 1.28 | 2.60 | 1.05 |
| AK107970 | 0.00 | 2.93 | E | 0.69 | 0.73 | 0.72 | 1.02 | 2.98 |
| AK103012 | 0.00 | 1.73 | D | 0.51 | 0.39 | 1.14 | 1.97 | 1.03 |
| AK061200 | 0.00 | 1.73 | E | 0.86 | 0.83 | 1.11 | 0.62 | 1.91 |
| AK108542 | 0.00 | 1.77 | C | 1.54 | 1.15 | 3.14 | 1.78 | 0.75 |
| AK073170 | 0.00 | 1.64 | E | 0.77 | 0.73 | 1.07 | 0.84 | 1.75 |
| AK101696 | 0.00 | 1.90 | D | 0.39 | 0.48 | 1.24 | 2.37 | 1.22 |
| AK066237 | 0.00 | 2.00 | B | 0.93 | 1.86 | 0.43 | 0.53 | 0.82 |
| AK098912 | 0.00 | 2.20 | C | 0.58 | 0.61 | 2.05 | 0.93 | 0.67 |
| AK070877 | 0.00 | 1.51 | C | 1.24 | 1.16 | 1.87 | 0.60 | 0.68 |
| AK064686 | 0.00 | 5.12 | E | 0.49 | 0.62 | 0.39 | 0.55 | 3.15 |
| AK111039 | 0.00 | 2.13 | A | 2.33 | 1.09 | 0.36 | 0.51 | 0.61 |
| AK062338 | 0.00 | 1.54 | E | 0.60 | 0.62 | 0.84 | 1.19 | 1.82 |
| AK066480 | 0.00 | 1.70 | D | 0.64 | 1.12 | 0.40 | 2.15 | 1.26 |
| AK101859 | 0.00 | 1.67 | E | 1.78 | 1.02 | 0.89 | 0.47 | 2.97 |
| AK071844 | 0.00 | 1.72 | D | 0.66 | 0.75 | 0.78 | 1.94 | 1.13 |
| AK107051 | 0.00 | 2.06 | D | 0.50 | 0.48 | 0.83 | 2.43 | 1.18 |
| AK067222 | 0.00 | 2.22 | E | 0.85 | 0.68 | 1.15 | 0.76 | 2.56 |
| AK072902 | 0.00 | 2.35 | D | 0.38 | 0.62 | 0.72 | 3.16 | 1.34 |
| AK064424 | 0.00 | 4.50 | E | 0.20 | 0.27 | 0.59 | 0.57 | 2.68 |
| AK069014 | 0.00 | 1.63 | D | 0.52 | 0.42 | 1.17 | 1.91 | 0.92 |
| AK100027 | 0.00 | 1.84 | A | 2.12 | 1.16 | 0.98 | 0.58 | 0.47 |
| AK061091 | 0.00 | 1.65 | C | 0.83 | 0.68 | 1.92 | 1.05 | 1.16 |
| AK101717 | 0.00 | 1.75 | D | 0.71 | 0.67 | 0.94 | 2.37 | 1.36 |
| AK061534 | 0.00 | 1.56 | D | 0.55 | 0.80 | 0.82 | 1.32 | 0.85 |
| AK100114 | 0.00 | 1.91 | C | 0.94 | 0.87 | 3.12 | 1.64 | 0.62 |
| AK108453 | 0.00 | 1.69 | D | 0.72 | 0.80 | 1.16 | 1.97 | 0.91 |
| AK108564 | 0.00 | 2.14 | C | 1.04 | 0.73 | 2.21 | 1.03 | 0.72 |
| AK058496 | 0.00 | 1.94 | A | 2.02 | 0.67 | 0.46 | 1.04 | 1.03 |
| AK058726 | 0.00 | 1.55 | B | 1.33 | 2.06 | 0.32 | 0.95 | 0.19 |
| AK068043 | 0.00 | 2.06 | E | 0.80 | 0.69 | 0.88 | 0.46 | 1.82 |
| AK071684 | 0.00 | 3.88 | E | 0.03 | 0.08 | 0.16 | 1.00 | 3.87 |
| AK063182 | 0.00 | 7.84 | E | 0.12 | 0.24 | 0.38 | 0.40 | 3.17 |
| AK109578 | 0.00 | 2.58 | E | 0.59 | 0.53 | 0.72 | 0.77 | 1.99 |
| AK073446 | 0.00 | 1.88 | C | 1.32 | 1.36 | 2.56 | 1.00 | 1.18 |
| AK071496 | 0.00 | 2.46 | D | 0.92 | 0.83 | 0.51 | 2.27 | 0.46 |
| AK062968 | 0.00 | 2.20 | D | 1.22 | 1.19 | 0.55 | 3.34 | 1.52 |
| AK099903 | 0.00 | 2.03 | E | 0.68 | 0.61 | 0.60 | 0.90 | 1.81 |
| AK107013 | 0.00 | 4.48 | E | 0.65 | 0.50 | 0.40 | 0.67 | 2.99 |
| AK100669 | 0.00 | 3.12 | C | 1.05 | 0.68 | 3.28 | 0.91 | 0.95 |
| AK063225 | 0.00 | 1.89 | E | 1.31 | 0.56 | 0.20 | 0.39 | 2.48 |
| AK070674 | 0.00 | 1.53 | E | 0.64 | 0.59 | 0.59 | 1.09 | 1.67 |
| AK099550 | 0.00 | 1.99 | D | 0.59 | 0.66 | 0.83 | 1.65 | 0.83 |
| AK073973 | 0.00 | 2.24 | D | 1.06 | 0.64 | 0.64 | 2.37 | 0.83 |
| AK062775 | 0.00 | 2.48 | E | 0.41 | 0.65 | 0.47 | 0.30 | 1.62 |
| AK063655 | 0.00 | 1.52 | C | 1.22 | 0.96 | 1.85 | 0.85 | 0.91 |
| AK111132 | 0.00 | 1.99 | C | 1.14 | 0.93 | 2.30 | 1.16 | 1.02 |
| AK061580 | 0.00 | 1.50 | D | 1.16 | 0.99 | 0.94 | 1.74 | 0.40 |
| AK106640 | 0.00 | 2.07 | C | 0.97 | 0.95 | 2.19 | 1.06 | 0.99 |
| AK061497 | 0.00 | 11.74 | E | 0.07 | 0.11 | 0.30 | 0.39 | 4.59 |
| AK108392 | 0.00 | 1.77 | E | 0.97 | 1.10 | 1.10 | 0.76 | 1.95 |
| AK066114 | 0.00 | 1.63 | D | 0.53 | 0.42 | 1.29 | 2.11 | 0.97 |
| AK070827 | 0.00 | 1.58 | E | 0.61 | 0.64 | 0.74 | 1.05 | 1.67 |
| AK061260 | 0.00 | 1.70 | C | 1.17 | 1.06 | 2.00 | 0.91 | 0.87 |
| AK100353 | 0.00 | 2.16 | D | 0.51 | 0.73 | 0.81 | 1.97 | 0.91 |
| AK059026 | 0.00 | 1.53 | C | 1.90 | 1.84 | 2.92 | 0.87 | 1.07 |
| AK105353 | 0.00 | 1.72 | C | 1.26 | 0.97 | 2.16 | 1.22 | 0.63 |
| AK062177 | 0.00 | 2.93 | C | 0.85 | 0.87 | 2.54 | 0.73 | 0.71 |
| AK071695 | 0.00 | 2.22 | C | 0.80 | 0.76 | 3.02 | 1.17 | 1.36 |
| AK101263 | 0.00 | 1.84 | E | 0.79 | 0.89 | 1.01 | 0.49 | 1.86 |
| AK073525 | 0.00 | 1.71 | E | 0.96 | 1.00 | 0.90 | 0.68 | 1.71 |
| AK069774 | 0.00 | 2.83 | D | 0.56 | 1.02 | 1.00 | 2.87 | 0.56 |
| AK073830 | 0.00 | 7.11 | E | 0.20 | 0.22 | 0.42 | 0.33 | 2.99 |
| AK102182 | 0.00 | 1.52 | D | 0.61 | 0.85 | 0.87 | 1.33 | 0.83 |
| AK071802 | 0.00 | 1.95 | E | 0.49 | 0.48 | 0.57 | 0.74 | 1.44 |
| AK062985 | 0.00 | 1.51 | C | 0.87 | 0.87 | 1.58 | 1.05 | 0.98 |
| AK060158 | 0.00 | 2.33 | E | 0.22 | 0.31 | 1.12 | 0.79 | 2.60 |
| AK066862 | 0.00 | 1.72 | E | 0.75 | 0.75 | 1.05 | 0.97 | 1.81 |
| AK061128 | 0.00 | 1.91 | D | 0.87 | 0.79 | 1.02 | 1.95 | 0.86 |
| AK064980 | 0.00 | 2.19 | E | 0.48 | 0.79 | 0.61 | 0.91 | 1.99 |
| AK103572 | 0.00 | 1.52 | E | 0.62 | 0.61 | 0.59 | 1.02 | 1.54 |
| AK067149 | 0.00 | 1.78 | C | 1.24 | 1.03 | 2.21 | 0.71 | 0.74 |
| AK110849 | 0.00 | 1.62 | C | 1.00 | 0.83 | 1.62 | 0.67 | 0.66 |
| AK068418 | 0.00 | 1.50 | E | 0.72 | 0.59 | 0.70 | 0.95 | 1.43 |
| AK070394 | 0.00 | 2.31 | E | 0.60 | 0.83 | 0.82 | 0.87 | 2.01 |
| AK101693 | 0.00 | 1.87 | C | 0.94 | 0.81 | 2.67 | 1.43 | 0.78 |
| AK101106 | 0.00 | 1.82 | C | 1.02 | 0.96 | 1.85 | 0.71 | 0.72 |
| AK066757 | 0.00 | 1.64 | E | 0.58 | 0.60 | 0.66 | 0.92 | 1.51 |
| AK061184 | 0.00 | 1.90 | D | 1.27 | 0.94 | 0.88 | 2.40 | 0.55 |
| AK099376 | 0.00 | 1.71 | C | 0.89 | 0.53 | 1.87 | 1.07 | 1.09 |
| AK110924 | 0.00 | 2.60 | E | 0.68 | 0.82 | 0.58 | 0.51 | 2.12 |
| AK060613 | 0.00 | 2.24 | D | 0.88 | 0.58 | 0.60 | 2.50 | 1.12 |
| AK068725 | 0.00 | 1.86 | D | 1.05 | 0.79 | 0.37 | 2.55 | 1.37 |
| AK071797 | 0.00 | 2.08 | A | 2.21 | 1.06 | 0.44 | 0.87 | 0.40 |
| AK061464 | 0.00 | 1.82 | C | 1.07 | 1.17 | 2.12 | 0.62 | 0.56 |
| AK099846 | 0.00 | 1.84 | D | 1.14 | 1.03 | 0.96 | 2.10 | 0.76 |
| AK064806 | 0.00 | 1.56 | E | 0.76 | 0.80 | 0.67 | 0.84 | 1.31 |
| AK109132 | 0.00 | 2.49 | E | 0.75 | 0.69 | 0.61 | 0.69 | 1.86 |
| AK103470 | 0.00 | 1.86 | A | 2.29 | 1.24 | 0.88 | 0.49 | 0.64 |
| AK111777 | 0.00 | 1.79 | E | 1.31 | 1.11 | 1.24 | 1.02 | 2.35 |
| AK107029 | 0.00 | 1.69 | C | 0.67 | 0.63 | 1.44 | 0.79 | 0.85 |
| AK058384 | 0.00 | 2.01 | E | 0.39 | 0.59 | 1.05 | 0.77 | 2.12 |
| AK066076 | 0.00 | 1.56 | E | 0.69 | 0.84 | 0.62 | 0.75 | 1.32 |
| AK102751 | 0.00 | 2.16 | B | 0.73 | 2.28 | 0.45 | 0.45 | 1.06 |
| AK101553 | 0.00 | 1.80 | E | 0.43 | 0.57 | 1.00 | 0.31 | 1.80 |
| AK060169 | 0.00 | 1.54 | D | 0.60 | 0.76 | 1.44 | 2.22 | 0.84 |
| AK061268 | 0.00 | 1.56 | D | 0.81 | 0.66 | 0.94 | 1.47 | 0.92 |
| AK103087 | 0.00 | 1.51 | D | 0.78 | 0.68 | 0.73 | 1.66 | 1.10 |
| AK068988 | 0.00 | 2.15 | E | 0.70 | 0.75 | 0.82 | 0.78 | 1.76 |
| AK105338 | 0.00 | 1.55 | E | 0.60 | 0.56 | 0.77 | 0.94 | 1.46 |
| AK063924 | 0.00 | 1.95 | D | 0.81 | 0.70 | 0.88 | 1.92 | 0.99 |
| AK060346 | 0.00 | 2.84 | E | 2.10 | 1.38 | 2.14 | 1.06 | 6.07 |
| AK068426 | 0.00 | 1.52 | A | 1.72 | 1.13 | 0.91 | 0.84 | 0.95 |
| AK063644 | 0.00 | 1.56 | C | 0.89 | 0.73 | 1.62 | 1.04 | 0.96 |
| AK101318 | 0.00 | 1.61 | D | 1.16 | 0.62 | 0.75 | 1.87 | 0.89 |
| AK103630 | 0.00 | 2.26 | D | 0.38 | 0.28 | 0.53 | 2.30 | 1.02 |
| AK069230 | 0.00 | 1.53 | E | 0.38 | 0.47 | 1.56 | 0.27 | 2.39 |
| AK066604 | 0.00 | 1.59 | D | 0.41 | 0.57 | 0.63 | 1.70 | 1.07 |
| AK072274 | 0.00 | 1.76 | E | 0.77 | 0.62 | 0.95 | 0.62 | 1.68 |
| AK111069 | 0.00 | 1.53 | C | 1.59 | 1.25 | 2.43 | 0.98 | 1.00 |
| AK101786 | 0.00 | 1.68 | C | 1.18 | 1.06 | 1.99 | 0.71 | 0.68 |
| AK073696 | 0.00 | 2.17 | C | 1.04 | 0.89 | 2.61 | 0.99 | 1.21 |
| AK062845 | 0.00 | 1.54 | A | 2.44 | 1.58 | 0.52 | 0.44 | 1.05 |
| AK100347 | 0.00 | 1.87 | D | 1.06 | 0.79 | 0.52 | 2.10 | 1.13 |
| AK070935 | 0.00 | 1.71 | D | 0.87 | 1.02 | 1.11 | 1.90 | 1.04 |
| AK110228 | 0.00 | 1.84 | E | 0.82 | 0.73 | 0.35 | 0.67 | 1.52 |
| AK102969 | 0.00 | 1.66 | B | 1.16 | 1.93 | 0.48 | 0.69 | 0.96 |
| AK102694 | 0.00 | 1.65 | D | 0.71 | 0.69 | 0.70 | 1.40 | 0.85 |
| AK072173 | 0.00 | 1.57 | D | 0.82 | 0.85 | 1.31 | 2.62 | 1.67 |
| AK102532 | 0.00 | 1.87 | E | 0.66 | 0.95 | 0.78 | 0.46 | 1.77 |
| AK099785 | 0.00 | 2.51 | D | 0.81 | 0.88 | 0.99 | 2.49 | 0.87 |
| AK107147 | 0.00 | 3.43 | D | 0.51 | 1.04 | 0.84 | 3.56 | 0.67 |
| AK065744 | 0.00 | 2.80 | E | 0.60 | 0.74 | 0.81 | 0.64 | 2.27 |
| AK105215 | 0.00 | 1.64 | E | 0.93 | 0.59 | 1.18 | 0.89 | 1.94 |
| AK061668 | 0.00 | 1.70 | D | 0.90 | 0.80 | 1.04 | 1.76 | 0.59 |
| AK103667 | 0.00 | 1.55 | A | 3.07 | 1.99 | 0.45 | 0.10 | 0.40 |
| AK071732 | 0.00 | 1.58 | E | 0.88 | 0.78 | 0.93 | 0.87 | 1.47 |
| AK067886 | 0.00 | 1.74 | C | 1.05 | 1.08 | 1.87 | 0.94 | 0.93 |
| AK072368 | 0.00 | 1.52 | D | 0.61 | 0.68 | 0.30 | 1.62 | 1.07 |
| AK099043 | 0.00 | 1.59 | D | 0.63 | 0.65 | 1.09 | 1.73 | 1.02 |
| AK101202 | 0.00 | 3.45 | D | 0.54 | 1.01 | 0.97 | 3.50 | 0.47 |
| AK099612 | 0.00 | 1.53 | C | 2.24 | 2.58 | 3.94 | 0.68 | 1.02 |
| AK101294 | 0.00 | 1.60 | E | 0.88 | 0.74 | 0.90 | 1.02 | 1.62 |
| AK067749 | 0.00 | 1.83 | E | 0.39 | 0.54 | 0.94 | 0.57 | 1.73 |
| AK101160 | 0.00 | 1.65 | D | 1.22 | 0.80 | 0.52 | 2.02 | 1.20 |
| AK106233 | 0.00 | 1.66 | A | 2.03 | 1.22 | 0.54 | 0.73 | 0.46 |
| AK063608 | 0.00 | 1.51 | D | 1.00 | 1.31 | 1.24 | 2.29 | 1.52 |
| AK111425 | 0.00 | 1.97 | C | 1.03 | 0.94 | 2.03 | 0.81 | 0.91 |
| AK058864 | 0.00 | 1.76 | C | 1.23 | 1.21 | 2.16 | 1.02 | 1.14 |
| AK060308 | 0.00 | 1.52 | C | 0.74 | 0.69 | 1.88 | 1.23 | 1.21 |
| AK102727 | 0.00 | 1.52 | C | 0.84 | 0.83 | 1.46 | 0.96 | 0.76 |
| AK063542 | 0.00 | 1.93 | E | 0.76 | 0.93 | 1.10 | 1.67 | 3.23 |
| AK059480 | 0.00 | 1.51 | D | 0.80 | 1.05 | 0.71 | 1.58 | 0.86 |
| AK066024 | 0.00 | 1.62 | C | 0.96 | 0.60 | 1.62 | 0.92 | 1.00 |
| AK069479 | 0.00 | 1.90 | E | 0.70 | 0.72 | 0.77 | 0.81 | 1.54 |
| AK100963 | 0.00 | 1.59 | A | 2.88 | 1.81 | 1.32 | 0.61 | 0.43 |
| AK073475 | 0.00 | 1.56 | E | 0.59 | 0.94 | 1.31 | 1.71 | 2.66 |
| AK100900 | 0.00 | 1.51 | D | 0.70 | 1.30 | 1.33 | 2.01 | 1.06 |
| AK070863 | 0.00 | 1.52 | E | 1.05 | 0.89 | 0.62 | 0.75 | 1.59 |
| AK106802 | 0.00 | 1.71 | C | 0.77 | 0.76 | 1.96 | 0.89 | 1.15 |
| AK107526 | 0.00 | 1.77 | A | 2.70 | 1.19 | 1.52 | 1.18 | 1.37 |
| AK102047 | 0.00 | 1.80 | C | 1.18 | 1.04 | 2.13 | 0.85 | 0.57 |
| AK102745 | 0.00 | 1.94 | D | 1.25 | 0.88 | 0.59 | 2.43 | 1.17 |
| AK073964 | 0.00 | 1.61 | E | 0.91 | 0.86 | 0.80 | 0.79 | 1.46 |
| AK067120 | 0.00 | 2.38 | D | 1.29 | 1.32 | 0.79 | 3.16 | 1.27 |
| AK063060 | 0.00 | 1.56 | E | 0.61 | 0.83 | 0.66 | 0.70 | 1.29 |
| AK062031 | 0.00 | 1.79 | D | 0.94 | 0.85 | 0.52 | 2.04 | 1.14 |
| AK069826 | 0.01 | 1.80 | C | 1.14 | 1.29 | 2.31 | 0.87 | 0.98 |
| AK058425 | 0.01 | 1.83 | E | 0.75 | 0.70 | 0.76 | 1.00 | 1.84 |
| AK062914 | 0.01 | 2.02 | E | 1.63 | 1.20 | 1.77 | 0.85 | 3.57 |
| AK107065 | 0.01 | 1.54 | C | 1.73 | 1.51 | 2.67 | 0.35 | 0.20 |
| AK066452 | 0.01 | 1.58 | C | 1.15 | 1.16 | 1.83 | 0.98 | 0.94 |
| AK106389 | 0.01 | 1.53 | A | 1.65 | 1.08 | 1.06 | 0.76 | 0.67 |
| AK110390 | 0.01 | 1.86 | D | 0.77 | 0.73 | 0.52 | 1.43 | 0.70 |
| AK068577 | 0.01 | 1.52 | D | 0.96 | 0.88 | 0.46 | 1.90 | 1.25 |
| AK101034 | 0.01 | 1.71 | C | 1.22 | 0.80 | 2.10 | 0.59 | 0.80 |
| AK110527 | 0.01 | 1.80 | D | 1.26 | 0.95 | 0.98 | 2.26 | 1.12 |
| AK071936 | 0.01 | 2.23 | D | 0.76 | 1.23 | 1.21 | 2.74 | 1.10 |
| AK101136 | 0.01 | 1.60 | C | 0.72 | 0.65 | 1.88 | 1.17 | 0.95 |
| AK109805 | 0.01 | 1.99 | D | 0.73 | 0.67 | 0.73 | 1.82 | 0.91 |
| AK063196 | 0.02 | 1.63 | E | 0.81 | 0.86 | 0.68 | 0.84 | 1.41 |
| AK107160 | 0.02 | 2.34 | D | 0.80 | 1.00 | 1.01 | 2.37 | 0.97 |
| AK105303 | 0.02 | 1.56 | E | 0.60 | 0.73 | 0.63 | 0.77 | 1.20 |
| AK108068 | 0.02 | 1.66 | C | 1.07 | 1.18 | 1.96 | 0.85 | 1.12 |
| AK068683 | 0.02 | 2.65 | D | 0.22 | 0.11 | 0.06 | 2.71 | 1.02 |
| AK102252 | 0.02 | 1.95 | D | 0.86 | 0.87 | 0.73 | 1.69 | 0.83 |
| AK068583 | 0.02 | 1.75 | E | 0.80 | 0.59 | 0.85 | 0.93 | 1.63 |
| AK059773 | 0.02 | 1.53 | C | 1.04 | 0.72 | 1.60 | 0.90 | 1.01 |
| AK068548 | 0.02 | 2.31 | E | 1.55 | 0.90 | 1.42 | 0.50 | 3.58 |
| AK105596 | 0.02 | 1.56 | C | 1.22 | 1.18 | 1.91 | 1.09 | 1.08 |
| AK070416 | 0.02 | 1.87 | D | 0.58 | 0.71 | 1.04 | 1.93 | 0.81 |
| AK066642 | 0.03 | 1.55 | A | 1.44 | 0.93 | 0.90 | 0.83 | 0.76 |
| AK101523 | 0.03 | 1.55 | C | 1.18 | 1.01 | 1.83 | 0.82 | 0.89 |
| AK109084 | 0.03 | 1.79 | A | 1.73 | 0.67 | 0.85 | 0.97 | 0.89 |
| AK064682 | 0.03 | 1.53 | D | 0.65 | 0.99 | 1.04 | 1.58 | 1.02 |
| AK111054 | 0.03 | 1.78 | D | 1.05 | 0.88 | 1.11 | 2.01 | 1.13 |
| AK071964 | 0.03 | 1.62 | E | 0.85 | 0.86 | 0.73 | 0.86 | 1.40 |
| AK105352 | 0.07 | 1.50 | C | 1.12 | 1.13 | 1.70 | 0.86 | 0.93 |
| AK109716 | 0.07 | 1.56 | C | 1.16 | 1.11 | 1.81 | 1.07 | 1.06 |
| AK106994 | 0.07 | 1.66 | D | 1.01 | 0.86 | 1.08 | 1.79 | 0.65 |
| AK100384 | 0.07 | 1.62 | D | 0.94 | 0.93 | 0.73 | 1.53 | 0.83 |
| AK068710 | 0.07 | 1.55 | A | 1.56 | 0.89 | 0.85 | 1.01 | 0.58 |
| AK107452 | 0.07 | 1.61 | E | 0.68 | 0.73 | 0.65 | 0.64 | 1.17 |
| AK106045 | 0.07 | 1.51 | C | 1.00 | 1.26 | 1.89 | 0.82 | 1.09 |
| AK100001 | 0.07 | 1.53 | D | 0.92 | 0.85 | 0.92 | 1.45 | 0.94 |
| AK071064 | 0.14 | 1.74 | C | 0.87 | 0.76 | 1.51 | 0.74 | 0.71 |
| AK060454 | 0.14 | 1.61 | D | 0.98 | 0.92 | 0.63 | 1.58 | 0.69 |
| AK101326 | 0.27 | 1.61 | E | 0.76 | 0.70 | 0.82 | 0.59 | 1.32 |
| AK068020 | 0.27 | 1.66 | C | 1.23 | 1.19 | 2.29 | 1.39 | 1.22 |
| AK099826 | 0.27 | 1.61 | D | 1.22 | 1.49 | 1.43 | 2.40 | 1.23 |
| AK065129 | 0.51 | 1.52 | C | 1.01 | 0.99 | 1.57 | 1.03 | 0.91 |
| AK072950 | 0.51 | 1.72 | D | 0.93 | 0.61 | 0.78 | 1.98 | 1.15 |
| AK060786 | 0.51 | 1.52 | B | 0.94 | 1.57 | 1.04 | 0.78 | 1.02 |
| AK066994 | 0.51 | 1.80 | C | 1.40 | 1.28 | 2.51 | 0.86 | 0.62 |
| AK073621 | 0.51 | 1.56 | B | 1.06 | 2.14 | 1.31 | 1.11 | 1.38 |
| AK059358 | 0.51 | 1.54 | D | 0.96 | 1.20 | 0.84 | 1.85 | 0.62 |
| AK058389 | 0.84 | 1.52 | D | 0.94 | 1.05 | 0.66 | 1.59 | 0.83 |
| AK103500 | 0.84 | 1.59 | C | 1.35 | 1.34 | 2.15 | 1.00 | 0.81 |
| AK067734 | 1.43 | 1.67 | E | 0.85 | 0.97 | 1.05 | 1.01 | 1.76 |

^a^ q-value is False Discovery Rate（FDR），q default 0.05, the less the q is, the more significant the genes enriched.

^b^ Fold Change indicates multiclass analysis of Significance Analysis of Microarrays (SAM).

^c^ Special Group indicates the specified tissue.

^d^ Avg_x represents the average ratio of the three biological replicates while A for Rhizome tips, B for Shoot tips, C for Rhizome internodes, D for Stem internodes and E for Young leaves.
