# Supplementary material for: Tissue-Specific Transcriptomic Profiling of Sorghum propinquum using a Rice Genome Array
Source: PLoS One. 2013 Mar 25;8(3):e60202. doi: 10.1371/journal.pone.0060202 (PMC3607598; doi:10.1371/journal.pone.0060202)
Supplement: Table S4 — The list of genes enriched specifically in the young leaf relative to other tissues. (DOC) [file pone.0060202.s005.doc]

**Table S4.** The list of genes enriched specifically in young leaf relative to other tissues.

| **Name** | **Oryza GI** | **Fold Change^a^** | **q-value (%)** | **Best Sorghum BLAST hit** | **Function Annotation** |
| --- | --- | --- | --- | --- | --- |
| AK074000 | LOC_Os07g26440 | 9.07 | 0 | Sb0073s002020 | expressed protein |
| AK064911 | LOC_Os04g23820 | 2.81 | 0 | Sb01g001060 | tRNA binding domain containing protein |
| AK106415 | LOC_Os03g58740 | 5 | 0 | Sb01g004790 | expressed protein |
| AK111777 | LOC_Os07g09000 | 1.79 | 0 | Sb01g004950 | WD domain, G-beta repeat domain containing protein |
| AK068261 | LOC_Os03g51430 | 3.12 | 0 | Sb01g009680 | SEC14 cytosolic factor family protein |
| AK103539 | LOC_Os03g48260 | 6.14 | 0 | Sb01g011640 | expressed protein |
| AK059143 | LOC_Os03g45710 | 1.64 | 0 | Sb01g012850 | 2Fe-2S iron-sulfur cluster binding domain containing protein |
| AK062338 | LOC_Os03g45340 | 1.54 | 0 | Sb01g012930 | hsp20/alpha crystallin family protein |
| AK062751 | LOC_Os07g01620 | 1.8 | 0 | Sb01g016760 | Dirigent-like protein |
| AK067223 | LOC_Os10g36870 | 4.08 | 0 | Sb01g017250 | src homology-3 domain protein 3 |
| AK102087 | LOC_Os10g34710 | 1.83 | 0 | Sb01g018770 | myosin-2 heavy chain, non muscle |
| AK059688 | LOC_Os10g22380 | 3.5 | 0 | Sb01g019490 | glutamyl-tRNA synthetase, cytoplasmic |
| AK062570 | LOC_Os03g07530 | 2.19 | 0 | Sb01g022460 | OsFBK12 - F-box domain and kelch repeat containing protein |
| AK073394 | LOC_Os10g07290 | 2.65 | 0 | Sb01g025310 | glycosyl hydrolases family 17 |
| AK073475 | LOC_Os10g42690 | 1.56 | 0 | Sb01g027940 | transcription factor jumonji |
| AK071546 | LOC_Os04g10160 | 7.55 | 0 | Sb01g032600 | cytochrome P450 |
| AK108125 | LOC_Os03g26490 | 10.44 | 0 | Sb01g033920 | expressed protein |
| AK063182 | LOC_Os03g24460 | 7.84 | 0 | Sb01g034820 | aminotransferase domain containing protein |
| AK058603 | LOC_Os03g26030 | 2.66 | 0 | Sb01g035060 | expressed protein |
| AK067222 | LOC_Os03g20640 | 2.22 | 0 | Sb01g036820 | expressed protein |
| AK071034 | LOC_Os03g17230 | 5.92 | 0 | Sb01g039050 | NAD dependent epimerase/dehydratase family protein |
| AK070827 | LOC_Os03g10620 | 1.58 | 0 | Sb01g043630 | hydrolase, alpha/beta fold family domain containing protein |
| AK072532 | LOC_Os08g25310 | 3.27 | 0 | Sb01g045510 | phosphatidylinositol transfer |
| AK065797 | LOC_Os03g05040 | 3.87 | 0 | Sb01g047320 | expressed protein |
| AK061292 | LOC_Os03g04660 | 4.82 | 0 | Sb01g047610 | Cytochrome P450 family protein |
| AK067991 | LOC_Os03g03650 | 7.78 | 0 | Sb01g048590 | POLD2 - Putative DNA polymerase delta complex subunit |
| AK101925 | LOC_Os03g02820 | 5.13 | 0 | Sb01g049210 | expressed protein |
| AK071075 | LOC_Os07g07550 | 1.74 | 0 | Sb02g004010 | galactose-1-phosphate uridyl transferase |
| AK100008 | LOC_Os07g08180 | 2.97 | 0 | Sb02g004410 | PPR repeat containing protein |
| AK071558 | LOC_Os09g39390 | 8.05 | 0 | Sb02g011895 | monodehydroascorbate reductase |
| AK069270 | LOC_Os09g39960 | 1.59 | 0 | Sb02g012940 | dynamin family protein |
| AK068988 | LOC_Os09g10940 | 2.15 | 0 | Sb02g018890 | ras-related protein |
| AK069027 | LOC_Os09g15430 | 2.53 | 0 | Sb02g021790 | zinc finger family protein |
| AK063358 | LOC_Os09g27910 | 1.67 | 0 | Sb02g026340 | aspartic proteinase nepenthesin precursor |
| AK071684 | LOC_Os09g37280 | 3.88 | 0 | Sb02g031670 | peroxisomal multifunctional enzyme type 2 |
| AK073402 | LOC_Os09g38310 | 2.66 | 0 | Sb02g032350 | pentatricopeptide |
| AK071565 | LOC_Os07g29640 | 2.01 | 0 | Sb02g033190 | expressed protein |
| AK063422 | LOC_Os07g29760 | 12.23 | 0 | Sb02g033270 | cysteine proteinase A494 precursor |
| AK106427 | LOC_Os12g21930 | 15.66 | 0 | Sb02g033980 | PPR repeat containing protein |
| AK110924 | LOC_Os07g31770 | 2.6 | 0 | Sb02g034010 | chalcone synthase |
| AK073881 | LOC_Os08g19694 | 12 | 0 | Sb02g034040 | NB-ARC domain containing protein |
| AK107291 | LOC_Os07g37690 | 2.41 | 0 | Sb02g034150 | UDP-glucoronosyl and UDP-glucosyl transferase |
| AK060302 | LOC_Os07g38830 | 3.77 | 0 | Sb02g037330 | hydrolase, alpha/beta fold family domain containing protein |
| AK065696 | LOC_Os07g41120 | 2.93 | 0 | Sb02g038590 | RNA recognition motif containing protein |
| AK100944 | LOC_Os01g11350 | 5.88 | 0 | Sb03g002050 | bZIP transcription factor domain containing protein |
| AK109578 | LOC_Os01g09100 | 2.58 | 0 | Sb03g003360 | OsWRKY10 - Superfamily of TFs having WRKY |
| AK072705 | LOC_Os01g07360 | 12.65 | 0 | Sb03g004710 | KIP1 |
| AK063435 | LOC_Os01g05830 | 3.05 | 0 | Sb03g005990 | expressed protein |
| AK108674 | LOC_Os05g47950 | 7.44 | 0 | Sb03g006390 | UDP-glucoronosyl and UDP-glucosyl transferase |
| AK110482 | LOC_Os01g04580 | 1.91 | 0 | Sb03g006700 | Ser/Thr protein kinase |
| AK103139 | LOC_Os01g17330 | 5.63 | 0 | Sb03g011410 | eukaryotic translation initiation factor 6 |
| AK109320 | LOC_Os09g25200 | 5.87 | 0 | Sb03g021250 | protein binding protein |
| AK101859 | LOC_Os01g39310 | 1.67 | 0 | Sb03g025850 | SEY1 |
| AK062372 | LOC_Os01g42280 | 9.37 | 0 | Sb03g027395 | pentatricopeptide |
| AK069479 | LOC_Os01g43590 | 1.9 | 0 | Sb03g028470 | HSF-type DNA-binding domain containing protein |
| AK106457 | LOC_Os01g47080 | 2.79 | 0 | Sb03g030110 | pyruvate kinase |
| AK103903 | LOC_Os01g50930 | 6.42 | 0 | Sb03g032400 | expressed protein |
| AK101326 | LOC_Os01g52630 | 1.61 | 0.27 | Sb03g033330 | regulator of chromosome condensation |
| AK111351 | LOC_Os01g53470 | 9.16 | 0 | Sb03g033900 | harpin-induced protein 1 domain containing protein |
| AK071964 | LOC_Os01g55750 | 1.62 | 0.03 | Sb03g035350 | TCP family transcription factor |
| AK103158 | LOC_Os01g59890 | 6.06 | 0 | Sb03g037820 | pentatricopeptide |
| AK072680 | LOC_Os01g64262 | 1.5 | 0 | Sb03g040680 | hydrolase |
| AK058384 | LOC_Os01g66420 | 2.01 | 0 | Sb03g042180 | PHD finger protein |
| AK107750 | LOC_Os04g32480 | 2.98 | 0 | Sb03g044485 | zinc-finger protein |
| AK108392 | LOC_Os01g72370 | 1.77 | 0 | Sb03g046090 | helix-loop-helix DNA-binding domain containing protein |
| AK068418 | LOC_Os02g07690 | 1.5 | 0 | Sb04g004860 | VQ domain containing protein |
| AK073830 | LOC_Os02g27950 | 7.11 | 0 | Sb04g019250 | ataxin-2 related protein |
| AK106534 | LOC_Os02g34850 | 1.67 | 0 | Sb04g022620 | histone-lysine N-methyltransferase ASHH2 |
| AK060291 | LOC_Os02g50480 | 3.45 | 0 | Sb04g028550 | histidine kinase |
| AK070394 | LOC_Os02g53670 | 2.31 | 0 | Sb04g034770 | MYB family transcription factor |
| AK069053 | LOC_Os02g55440 | 4.83 | 0 | Sb04g036080 | transmembrane 9 superfamily member |
| AK066076 | LOC_Os09g34040 | 1.56 | 0 | Sb04g037270 | TBC domain containing protein |
| AK061200 | LOC_Os11g27370 | 1.73 | 0 | Sb05g002840 | UDP-glucoronosyl and UDP-glucosyl transferase |
| AK064254 | LOC_Os11g05650 | 2.12 | 0 | Sb05g003425 | mRNA-decapping enzyme |
| AK102308 | LOC_Os11g07040 | 1.51 | 0 | Sb05g004610 | CAMK includes calcium/calmodulin depedent protein kinases |
| AK064424 | LOC_Os01g66230 | 4.5 | 0 | Sb05g004750 | csAtPR5 |
| AK068331 | LOC_Os11g10420 | 7.25 | 0 | Sb05g009420 | phosphatidylinositol kinase |
| AK107902 | LOC_Os04g57130 | 5.76 | 0 | Sb05g022150 | dirigent-like protein pDIR17 |
| AK068548 | LOC_Os11g38010 | 2.31 | 0.02 | Sb05g022990 | targeting protein for Xklp2 |
| AK065824 | LOC_Os11g39190 | 11.49 | 0 | Sb05g024020 | NB-ARC domain containing protein |
| AK066862 | LOC_Os04g20164 | 1.72 | 0 | Sb06g004290 | amine oxidase precursor |
| AK068516 | LOC_Os05g03140 | 1.5 | 0 | Sb06g016555 | tetraspanin family protein |
| AK063934 | LOC_Os04g35520 | 4.63 | 0 | Sb06g017080 | OsAPx7 - Stromal Ascorbate Peroxidase encoding gene 5 |
| AK060688 | LOC_Os04g39090 | 8.26 | 0 | Sb06g019260 | SAM domain containing protein |
| AK073406 | LOC_Os04g41110 | 1.77 | 0 | Sb06g020870 | Rad21 / Rec8 like protein |
| AK103837 | LOC_Os04g42330 | 1.56 | 0 | Sb06g021710 | Spc97 / Spc98 family protein |
| AK070863 | LOC_Os04g42770 | 1.52 | 0 | Sb06g021940 | expressed protein |
| AK071791 | LOC_Os04g43290 | 3.12 | 0 | Sb06g022330 | ARPC2B |
| AK103452 | LOC_Os04g56850 | 2.05 | 0 | Sb06g031900 | auxin response factor |
| AK069249 | LOC_Os04g57370 | 2.15 | 0 | Sb06g032240 | acyl-protein thioesterase |
| AK073170 | LOC_Os04g57520 | 1.64 | 0 | Sb06g032400 | UBX domain-containing protein |
| AK103572 | LOC_Os06g47000 | 1.52 | 0 | Sb07g003070 | external NADH-ubiquinone oxidoreductase 1 |
| AK100477 | LOC_Os01g05620 | 6.23 | 0 | Sb07g007000 | NBS-LRR disease resistance protein |
| AK108397 | LOC_Os08g29720 | 4.96 | 0 | Sb07g019430 | mitochondrial carrier protein |
| AK105338 | LOC_Os08g43670 | 1.55 | 0 | Sb07g024770 | RING-H2 finger protein ATL2B |
| AK071527 | LOC_Os08g42370 | 2.15 | 0 | Sb07g026030 | zinc finger DHHC domain-containing protein |
| AK060537 | LOC_Os02g19970 | 7.73 | 0 | Sb08g000600 | aminotransferase |
| AK111498 | LOC_Os12g18120 | 1.59 | 0 | Sb08g012360 | zinc finger C-x8-C-x5-C-x3-H type family protein |
| AK058220 | LOC_Os12g32620 | 2.69 | 0 | Sb08g016450 | OsWLIM1 - LIM domain protein |
| AK099628 | LOC_Os12g42860 | 1.57 | 0 | Sb08g022140 | 2-aminoethanethiol dioxygenase |
| AK100940 | LOC_Os12g43700 | 4.05 | 0 | Sb08g022830 | SCP-like extracellular protein |
| AK067749 | LOC_Os05g02010 | 1.83 | 0 | Sb09g001176 | expressed protein |
| AK101105 | LOC_Os05g05720 | 3.13 | 0 | Sb09g003840 | tetratricopeptide repeat domain containing protein |
| AK106800 | LOC_Os05g06660 | 1.89 | 0 | Sb09g004510 | OsSCP26 - Putative Serine Carboxypeptidase homologue |
| AK101553 | LOC_Os05g25840 | 1.8 | 0 | Sb09g011240 | ELMO/CED-12 family protein |
| AK101263 | LOC_Os05g28980 | 1.84 | 0 | Sb09g017540 | HRB1, drought induced 19 protein |
| AK064686 | LOC_Os05g30580 | 5.12 | 0 | Sb09g018380 | OsSub46 - Putative Subtilisin homologue |
| AK066175 | LOC_Os05g01990 | 5.73 | 0 | Sb09g020890 | DEAD-box ATP-dependent RNA helicase |
| AK109649 | LOC_Os05g40950 | 3.21 | 0 | Sb09g023820 | pentatricopeptide repeat-containing protein |
| AK069746 | LOC_Os05g41150 | 4.1 | 0 | Sb09g023980 | expressed protein |
| AK065638 | LOC_Os05g43300 | 8.38 | 0 | Sb09g024990 | expressed protein |
| AK072274 | LOC_Os05g43490 | 1.76 | 0 | Sb09g025120 | OsFBX169 - F-box domain containing protein |
| AK069230 | LOC_Os05g48210 | 1.53 | 0 | Sb09g027920 | expressed protein |
| AK067992 | LOC_Os05g51630 | 1.58 | 0 | Sb09g030770 | early-responsive to dehydration protein-related |
| AK073964 | LOC_Os05g37500 | 1.61 | 0 | Sb10g000410 | expressed protein |
| AK058892 | LOC_Os06g03682 | 6.12 | 0 | Sb10g001640 | calcium-dependent protein kinase isoform AK1 |
| AK105303 | LOC_Os06g04080 | 1.56 | 0.02 | Sb10g002130 | glycosyl hydrolases family 17 |
| AK109132 | LOC_Os06g04480 | 2.49 | 0 | Sb10g002440 | expressed protein |
| AK111868 | LOC_Os03g63260 | 7.46 | 0 | Sb10g002910 | pentatricopeptide |
| AK099903 | LOC_Os06g06014 | 2.03 | 0 | Sb10g003760 | expressed protein |
| AK101097 | LOC_Os06g06120 | 1.98 | 0 | Sb10g003820 | expressed protein |
| AK061497 | LOC_Os06g06250 | 11.74 | 0 | Sb10g003890 | GDSL-like lipase/acylhydrolase |
| AK065744 | LOC_Os06g06980 | 2.8 | 0 | Sb10g004540 | caffeoyl-CoA O-methyltransferase |
| AK058385 | LOC_Os06g12280 | 11.5 | 0 | Sb10g008060 | glycosyl transferase 8 domain containing protein |
| AK071914 | LOC_Os06g10170 | 5.49 | 0 | Sb10g009680 | FMO1, flavin-containing monooxygenase family protein |
| AK107013 | LOC_Os06g33330 | 4.48 | 0 | Sb10g020420 | powdery mildew resistant protein 5 |
| AK109943 | LOC_Os06g34430 | 3.71 | 0 | Sb10g021020 | zinc finger protein |
| AK070840 | LOC_Os06g41710 | 2.07 | 0 | Sb10g024390 | CW-type Zinc Finger |
| AK071732 | LOC_Os06g43630 | 1.58 | 0 | Sb10g025240 | sucrose-phosphate synthase |
| AK108015 | LOC_Os06g46570 | 3.23 | 0 | Sb10g027290 | galactosyltransferase |
| AK104993 | LOC_Os06g46820 | 5.59 | 0 | Sb10g027500 | solute carrier family 35 member F5 |
| AK071855 | LOC_Os06g47220 | 2.57 | 0 | Sb10g027850 | expressed protein |
| AK063542 | LOC_Os06g49670 | 1.93 | 0 | Sb10g029640 | pentatricopeptide |
| AK062526 | LOC_Os03g63390 | 5.72 | 0 | unknown | Chemocyanin precursor |
| AK061089 | LOC_Os05g27110 | 13.68 | 0 | unknown | hypothetical protein |
| AK061510 | LOC_Os07g36800 | 4.26 | 0 | unknown | Uncharacterised protein family containing protein |
| AK099349 | LOC_Os10g30970 | 338.89 | 0 | unknown | expressed protein |
| AK110880 | LOC_Os12g22600 | 15.42 | 0 | unknown | tRNA synthetases class II family protein |
| AK111012 | Os10g0128100 | 7.46 | 0 | unknown | unknown |
| AK071874 | LOC_Os12g07700 | 5.4 | 0 | unknown | nitrogen fixation protein |
| AK066957 | Os11g0471200 | 7.99 | 0 | unknown | Conserved hypothetical protein. |
| AK109532 | LOC_Os02g45270 | 2.67 | 0 | unknown | expressed protein |
| AK111236 | unknown | 6.45 | 0 | unknown | unknown |
| AK110675 | LOC_Os03g06674 | 4.35 | 0 | unknown | unknown |
| AK072350 | LOC_Os02g49670 | 5.61 | 0 | unknown | Zinc knuckle family protein |
| AK102181 | LOC_Os05g46950 | 4.63 | 0 | unknown | expressed protein |
| AK099963 | Os03g0265900 | 11.45 | 0 | unknown | unknown |
| AK111051 | LOC_Os09g23770 | 6.61 | 0 | unknown | expressed protein |
| AK062634 | LOC_Os07g44720 | 2.83 | 0 | unknown | retrotransposon protein |
| AK061068 | LOC_Os10g40710 | 1.58 | 0 | unknown | Beta-expansin 1a precursor |
| AK072782 | LOC_Os11g03070 | 5.58 | 0 | unknown | ATCHX |
| AK073888 | LOC_Os08g04240 | 4.07 | 0 | unknown | 33 kDa secretory protein |
| AK059972 | unknown | 7.4 | 0 | unknown | unknown |
| AK107560 | LOC_Os06g46480 | 6.24 | 0 | unknown | Beige/BEACH domain containing protein |
| AK071000 | LOC_Os08g19670 | 9.91 | 0 | unknown | expressed protein |
| AK110269 | LOC_Os10g25170 | 4.39 | 0 | unknown | AP2 domain containing protein |
| AK073278 | LOC_Os07g43240 | 2.81 | 0 | unknown | Skp1 family |
| AK064122 | LOC_Os02g56830 | 2.07 | 0 | unknown | transposon protein |
| AK067485 | Os07g0194400 | 4.3 | 0 | unknown | Conserved hypothetical protein. |
| AK065598 | LOC_Os11g35980 | 2.19 | 0 | unknown | Leucine Rich Repeat family protein |
| AK059969 | LOC_Os11g03390 | 4.17 | 0 | unknown | FHA domain containing protein |
| AK104720 | unknown | 1.77 | 0 | unknown | unknown |
| AK062879 | LOC_Os02g21470 | 1.5 | 0 | unknown | expressed protein |
| AK066341 | LOC_Os06g49360 | 4.2 | 0 | unknown | NB-ARC domain containing protein |
| AK068857 | LOC_Os09g28040 | 6.24 | 0 | unknown | unknown |
| AK072777 | LOC_Os02g37862 | 1.7 | 0 | unknown | unknown |
| AK066728 | LOC_Os07g01904 | 1.51 | 0 | unknown | unknown |
| AK108828 | unknown | 2.34 | 0 | unknown | unknown |
| AK071590 | LOC_Os10g05790 | 2.93 | 0 | unknown | expressed protein |
| AK100550 | LOC_Os01g06600 | 6.42 | 0 | unknown | Acyl-coenzyme A oxidase 4 |
| AK106807 | Os09g0567600 | 2.65 | 0 | unknown | Hypothetical protein. |
| AK105248 | LOC_Os01g12810 | 1.77 | 0 | unknown | leaf protein |
| AK072876 | LOC_Os11g29500 | 1.59 | 0 | unknown | expressed protein |
| AK064274 | LOC_Os09g37344 | 3.58 | 0 | unknown | unknown |
| AK099305 | unknown | 1.74 | 0 | unknown | unknown |
| AK066992 | Os04g0461400 | 3.56 | 0 | unknown | Hypothetical protein. |
| AK100513 | LOC_Os03g21740 | 1.94 | 0 | unknown | Valyl-tRNA synthetase |
| AK063068 | unknown | 1.54 | 0 | unknown | unknown |
| AK073574 | LOC_Os05g16670 | 4 | 0 | unknown | SHR5-receptor-like kinase |
| AK060218 | LOC_Os07g32760 | 3.57 | 0 | unknown | Bromodomain containing protein |
| AK102049 | LOC_Os06g41670 | 2.1 | 0 | unknown | NB-ARC domain containing protein |
| AK064688 | LOC_Os03g64014 | 2.94 | 0 | unknown | hypothetical protein |
| AK102912 | LOC_Os01g32849 | 3.63 | 0 | unknown | hypothetical protein |
| AK109120 | unknown | 4.56 | 0 | unknown | unknown |
| AK106130 | LOC_Os05g51590 | 7.59 | 0 | unknown | B2 protein |
| AK107184 | LOC_Os04g44780 | 2.82 | 0 | unknown | Rare lipoprotein A like double-psi beta-barrel containing protein |
| AK064432 | LOC_Os10g20480 | 5.12 | 0 | unknown | transposon protein |
| AK110966 | LOC_Os05g38280 | 2.05 | 0 | unknown | hypothetical protein |
| AK066324 | LOC_Os02g20330 | 1.5 | 0 | unknown | hypothetical protein |
| AK064230 | Os01g0719200 | 2.3 | 0 | unknown | Conserved hypothetical protein. |
| AK073452 | Os02g0180800 | 1.67 | 0 | unknown | hypothetical protein |
| AK101462 | Os04g0367800 | 2.81 | 0 | unknown | HAT dimerisation domain containing protein. |
| AK062659 | LOC_Os05g01330 | 4.75 | 0 | unknown | expressed protein |
| AK058313 | LOC_Os01g10400 | 4.73 | 0 | unknown | expressed protein |
| AK072652 | Os12g0254500 | 1.63 | 0 | unknown | hypothetical protein |
| AK107970 | Os01g0679400 | 2.93 | 0 | unknown | hypothetical protein |
| AK068043 | LOC_Os02g06790 | 2.06 | 0 | unknown | expressed protein |
| AK063225 | LOC_Os09g04924 | 1.89 | 0 | unknown | Hypothetical protein. |
| AK070674 | LOC_Os02g16680 | 1.53 | 0 | unknown | bZIP transcription factor family protein |
| AK062775 | Os04g0546900 | 2.48 | 0 | unknown | ethylene-responsive transcription factor |
| AK073525 | LOC_Os01g49110 | 1.71 | 0 | unknown | protein prenyltransferase alpha subunit |
| AK071802 | Os10g0578700 | 1.95 | 0 | unknown | Conserved hypothetical protein. |
| AK060158 | LOC_Os07g44840 | 2.33 | 0 | unknown | GAMMA_CA2, Protein yrdA |
| AK064980 | LOC_Os07g06500 | 2.19 | 0 | unknown | F-box domain containing protein |
| AK066757 | LOC_Os01g71930 | 1.64 | 0 | unknown | beta-1 |
| AK064806 | LOC_Os09g17329 | 1.56 | 0 | unknown | hypothetical protein |
| AK060346 | unknown | 2.84 | 0 | unknown | unknown |
| AK110228 | unknown | 1.84 | 0 | unknown | unknown |
| AK102532 | LOC_Os05g18274 | 1.87 | 0 | unknown | hypothetical protein |
| AK105215 | LOC_Os12g35590 | 1.64 | 0 | unknown | expressed protein |
| AK101294 | LOC_Os02g32230 | 1.6 | 0 | unknown | retrotransposon protein |
| AK063060 | LOC_Os12g11660 | 1.56 | 0 | unknown | expressed protein |
| AK058425 | LOC_Os06g03120 | 1.83 | 0.01 | unknown | Aspartic proteinase nepenthesin-2 precursor |
| AK062914 | LOC_Os09g25720 | 2.02 | 0.01 | unknown | expressed protein |
| AK063196 | Os10g0505700 | 1.63 | 0.02 | unknown | LTPL160 - Protease inhibitor/seed storage |
| AK068583 | LOC_Os02g42320 | 1.75 | 0.02 | unknown | Proteasome subunit alpha type 2 |
| AK107452 | Os04g0435200 | 1.61 | 0.07 | unknown | Hypothetical protein. |
| AK067734 | LOC_Os02g48400 | 1.67 | 1.43 | unknown | expressed protein |

^a^ Fold Change represents the ratio of Avg_YL vs. MAX (Avg_ST, Avg_RI, Avg_SI, and Avg_RT), and q-value (%) ≤5 %, while Avg_x represents the average ratio of the

three biological replicates while RT for Rhizome tips/control, ST for Shoot tips/control, RI for Rhizome internodes/control, SI for Stem internodes/control and YL for Young

leaves/control.
