# Supplementary material for: Tissue-Specific Transcriptomic Profiling of Sorghum propinquum using a Rice Genome Array
Source: PLoS One. 2013 Mar 25;8(3):e60202. doi: 10.1371/journal.pone.0060202 (PMC3607598; doi:10.1371/journal.pone.0060202)
Supplement: Table S5 — The list of genes enriched specifically in shoot tips relative to other tissues. (DOC) [file pone.0060202.s006.doc]

Table S5. The list of genes enriched specifically in shoot tips relative to other tissues.
Name	Oryza GI	Fold Changea	q-value(%)	Best Sorghum BLAST hit	Function Annotation	
AK109814	LOC_Os03g62860	1.52	0	Sb01g001430	Glycosyl hydrolases family 17 protein, expressed	
AK066208	LOC_Os10g25430	1.79	0	Sb01g023200	hypothetical protein	
AK063764	LOC_Os01g43750	1.57	0	Sb01g036520	Cytochrome P450 family protein, expressed	
AK060786	LOC_Os09g25850	1.52	0.51	Sb02g025220	Gl1 protein, putative, expressed	
AK060943	LOC_Os01g13570	1.52	0	Sb03g000470	phosphoglycerate mutase family protein, expressed	
AK059965	LOC_Os01g50460	1.51	0	Sb03g032190	MtN3/saliva family protein, expressed	
AK068432	LOC_Os01g67720	1.58	0	Sb03g043040	ABC1 family protein, expressed	
AK071006	LOC_Os02g03330	1.82	0	Sb04g002190	Lil3 protein, putative, expressed	
AK110871	LOC_Os02g10770	2.01	0	Sb04g006980	DEAD/DEAH box helicase family protein, putative, expressed	
AK058726	LOC_Os02g49680	1.55	0	Sb04g029100	calcium sensing receptor, putative, expressed	
AK070539	LOC_Os04g58570	1.93	0	Sb06g033270	C2 domain protein At1g63220, putative, expressed	
AK073621	LOC_Os12g31510	1.56	0.51	Sb08g015490	expressed protein	
AK060457	LOC_Os05g32570	1.65	0	Sb09g019370	E3 ubiquitin-protein ligase KEG	
AK064752	LOC_Os06g11140	1.75	0	Sb10g007230	NADH dehydrogenase, putative, expressed	
AK067292	LOC_Os04g41510 	1.86	0	unknown	RWD domain containing protein, expressed	
AK108458	LOC_Os09g36750	1.84	0	unknown	L-ascorbate peroxidase 4, putative, expressed	
AK062918	LOC_Os01g13090	1.63	0	unknown	expressed protein	
AK109473	Os06g0647600	1.74	0	unknown	unknown	
AK107040	LOC_Os08g16720 	1.83	0	unknown	transposon protein, putative, CACTA, En/Spm sub-class, expressed	
AK073991	Os03g0602500	1.53	0	unknown	Hypothetical protein.	
AK063762	LOC_Os06g23870	1.51	0	unknown	Acyl-coenzyme A oxidase 4, peroxisomal, putative, expressed	
AK111062	Os12g0442700	1.80	0	unknown	Hypothetical protein.	
AK067080	Os11g0139700	1.60	0	unknown	unknown	
AK066237	LOC_Os07g35510	2.00	0	unknown	unknown	
AK102751		2.16	0	unknown	unknown	
AK102969	Os07g0220600	1.66	0	unknown	unknown	
aFold Change represents the ratio of Avg_ST vs. MAX (Avg_RT, Avg_RI, Avg_SI, and Avg_YL), and q-value (%) ¡Ü5 %, while Avg_x represents the average ratio of the three biological replicates while RT for Rhizome tips/control, ST for Shoot tips/control, RI for Rhizome internodes/control, SI for Stem internodes/control and YL for Young leaves/control.
