# Supplementary material for: Tissue-Specific Transcriptomic Profiling of Sorghum propinquum using a Rice Genome Array
Source: PLoS One. 2013 Mar 25;8(3):e60202. doi: 10.1371/journal.pone.0060202 (PMC3607598; doi:10.1371/journal.pone.0060202)
Supplement: Table S6 — The list of genes enriched specifically in shoot internodes relative to other tissues. (DOC) [file pone.0060202.s007.doc]

**Table S6.** The list of genes enriched specifically in shoot internodes relative to other tissues.

| **Name** | **Oryza GI** | **Fold Change^a^** | **q-value(%)** | **Best Sorghum BLAST hit** | **Function Annotation** |
| --- | --- | --- | --- | --- | --- |
| AK066480 | LOC_Os10g10170 | 1.7 | 0 | Sb0012s017730 | pentatricopeptide, putative, expressed |
| AK099043 | LOC_Os03g61260 | 1.59 | 0 | Sb01g002595 | 50S ribosomal protein L18, chloroplast precursor, putative, expressed |
| AK071181 | LOC_Os03g55420 | 1.81 | 0 | Sb01g007230 | Peroxidase 35 precursor, putative, expressed |
| AK101646 | LOC_Os03g55350 | 2.03 | 0 | Sb01g007310 | cucumisin-like serine protease, putative, expressed |
| AK062628 | LOC_Os03g52400 | 1.68 | 0 | Sb01g008970 | Ribosomal protein L7/L12 C-terminal domain containing protein, expressed |
| AK111437 | LOC_Os03g48380 | 5.5 | 0 | Sb01g011540 | expressed protein |
| AK063303 | LOC_Os10g28610 | 1.73 | 0 | Sb01g021680 | expressed protein |
| AK067754 | LOC_Os10g27450 | 1.8 | 0 | Sb01g022060 | expressed protein |
| AK101318 | LOC_Os10g39220 | 1.61 | 0 | Sb01g030490 | tobamovirus multiplication protein 3, putative, expressed |
| AK066604 | LOC_Os03g21980 | 1.59 | 0 | Sb01g035980 | tRNA pseudouridine synthase family protein, expressed |
| AK105244 | LOC_Os03g21710 | 1.71 | 0 | Sb01g036180 | WRKY DNA binding domain containing protein |
| AK068500 | LOC_Os03g07340 | 2.14 | 0 | Sb01g045880 | 50S ribosomal protein L18, chloroplast precursor, putative, expressed |
| AK109394 | LOC_Os03g05570 | 1.68 | 0 | Sb01g046930 | Zinc finger, C3HC4 type family protein, expressed |
| AK061250 | LOC_Os03g05280 | 3.55 | 0 | Sb01g047150 | Ras family protein, expressed |
| AK066607 | LOC_Os03g04960 | 1.54 | 0 | Sb01g047370 | Cysteinyl-tRNA synthetase, putative, expressed |
| AK063608 | LOC_Os03g04080 | 1.51 | 0 | Sb01g048120 | expressed protein |
| AK102745 | LOC_Os01g05960 | 1.94 | 0 | Sb02g006250 | Leucine Rich Repeat family protein, expressed |
| AK071844 | LOC_Os07g19460 | 1.72 | 0 | Sb02g009480 | Mitochondrial carrier protein, expressed |
| AK059358 | LOC_Os09g08130 | 1.54 | 0.51 | Sb02g020430 | Indole-3-glycerol phosphate synthase, chloroplast precursor |
| AK099826 | LOC_Os09g19560 | 1.61 | 0.27 | Sb02g022770 | Protein arginine N-methyltransferase 1, putative, expressed |
| AK099811 | LOC_Os09g27210 | 1.93 | 0 | Sb02g026010 | lecithine cholesterol acyltransferase, putative, expressed |
| AK099846 | LOC_Os12g17880 | 1.84 | 0 | Sb02g032190 | U-box domain containing protein, expressed |
| AK059647 | LOC_Os02g18550 | 1.85 | 0 | Sb02g038380 | 40S ribosomal protein S3a, putative, expressed |
| AK063924 | LOC_Os07g41260 | 1.95 | 0 | Sb02g038690 | pentatricopeptide, putative, expressed |
| AK064433 | LOC_Os04g46310 | 1.51 | 0 | Sb03g005100 | HEAT repeat family protein, expressed |
| AK060305 | LOC_Os01g06220 | 1.89 | 0 | Sb03g005560 | PrMC3, putative, expressed |
| AK108287 | LOC_Os01g01350 | 1.77 | 0 | Sb03g009000 | SNF7 family protein, expressed |
| AK105594 | LOC_Os01g21610 | 1.91 | 0 | Sb03g013060 | Chaperone-activity of bc1 complex-like, mitochondrial precursor |
| AK101185 | LOC_Os02g27360 | 1.75 | 0 | Sb03g026140 | aspartyl protease family protein, putative, expressed |
| AK069774 | LOC_Os01g40280 | 2.83 | 0 | Sb03g026180 | integral to membrane protein, putative, expressed |
| AK110703 | LOC_Os01g44394 | 2.96 | 0 | Sb03g028970 | Unknown |
| AK101504 | LOC_Os01g45550 | 1.68 | 0 | Sb03g029320 | auxin efflux carrier protein, putative, expressed |
| AK060930 | LOC_Os01g48820 | 1.64 | 0 | Sb03g030980 | expressed protein |
| AK069992 | LOC_Os01g50810 | 5.82 | 0 | Sb03g032300 | pectinesterase inhibitor domain containing protein, expressed |
| AK059864 | LOC_Os01g57890 | 1.61 | 0 | Sb03g036820 | START domain containing protein, expressed |
| AK071859 | LOC_Os01g59930 | 1.62 | 0 | Sb03g037870 | oxidoreductase, FAD-binding family protein, expressed |
| AK071598 | LOC_Os01g60740 | 2.07 | 0 | Sb03g038280 | Nonspecific lipid-transfer protein precursor, putative, expressed |
| AK060613 | LOC_Os01g61010 | 2.24 | 0 | Sb03g038420 | Nodulin-like family protein, expressed |
| AK111988 | LOC_Os01g63460 | 1.79 | 0 | Sb03g040160 | myb family transcription factor, putative, expressed |
| AK061128 | LOC_Os01g63930 | 1.91 | 0 | Sb03g040440 | Cytochrome P450 family protein, expressed |
| AK102845 | LOC_Os01g65986 | 1.82 | 0 | Sb03g041800 | Unknown |
| AK070795 | LOC_Os01g69870 | 2.03 | 0 | Sb03g044190 | Unknown |
| AK066987 | LOC_Os01g72820 | 2.4 | 0 | Sb03g046420 | CRS1/YhbY domain containing protein, expressed |
| AK069014 | LOC_Os02g01960 | 1.63 | 0 | Sb04g000780 | Kinesin light chain, putative, expressed |
| AK070032 | LOC_Os02g07490 | 1.67 | 0 | Sb04g004750 | Glyceraldehyde-3-phosphate dehydrogenase, cytosolic, putative, expressed |
| AK102694 | LOC_Os02g09930 | 1.65 | 0 | Sb04g006260 | glycosyltransferase 10, putative, expressed |
| AK072950 | LOC_Os11g33240 | 1.72 | 0.51 | Sb04g006440 | Citrate synthase 4, mitochondrial precursor, putative, expressed |
| AK072368 | LOC_Os02g10770 | 1.52 | 0 | Sb04g006980 | DEAD/DEAH box helicase family protein, putative, expressed |
| AK108786 | LOC_Os02g30810 | 2.51 | 0 | Sb04g020740 | Auxin responsive protein, expressed |
| AK102182 | LOC_Os02g36350 | 1.52 | 0 | Sb04g023650 | expressed protein |
| AK060204 | LOC_Os02g37830 | 1.95 | 0 | Sb04g024470 | Protein kinase domain containing protein, expressed |
| AK106596 | LOC_Os02g42300 | 2.32 | 0 | Sb04g026890 | expressed protein |
| AK100709 | LOC_Os02g47170 | 1.54 | 0 | Sb04g030860 | expressed protein |
| AK101160 | LOC_Os02g43430 | 1.65 | 0 | Sb04g033310 | Protein kinase APK1B, chloroplast precursor, putative, expressed |
| AK099785 | LOC_Os03g52594 | 2.51 | 0 | Sb04g035110 | Unknown |
| AK073365 | LOC_Os02g56120 | 1.86 | 0 | Sb04g036430 | AUX/IAA family protein, expressed |
| AK058331 | LOC_Os02g57100 | 1.52 | 0 | Sb04g037110 | haloacid dehalogenase-like hydrolase family protein, putative, expressed |
| AK061268 | LOC_Os02g58480 | 1.56 | 0 | Sb04g038410 | Sucrose synthase 2, putative, expressed |
| AK107147 | LOC_Os02g58610 | 3.43 | 0 | Sb04g038550 | Unknown |
| AK070935 | LOC_Os11g03060 | 1.71 | 0 | Sb05g001480 | SNF7 family protein, expressed |
| AK061668 | LOC_Os11g05110 | 1.7 | 0 | Sb05g002900 | pyruvate kinase family protein, expressed |
| AK058389 | LOC_Os12g05550 | 1.52 | 0.84 | Sb05g003150 | sialyltransferase, putative, expressed |
| AK106448 | LOC_Os12g07590 | 2.19 | 0 | Sb05g005210 | Protein-tyrosine phosphatase containing protein, expressed |
| AK058342 | LOC_Os11g10780 | 3.73 | 0 | Sb05g009053 | Ulp1 protease family protein, putative, expressed |
| AK100587 | LOC_Os03g36830 | 2.58 | 0 | Sb05g021062 | expressed protein |
| AK070903 | LOC_Os11g38980 | 1.64 | 0 | Sb05g023750 | kelch repeat-containing F-box family protein, putative, expressed |
| AK111054 | LOC_Os04g01570 | 1.78 | 0.03 | Sb06g000550 | pectinesterase inhibitor domain containing protein, expressed |
| AK068725 | LOC_Os04g35700 | 1.86 | 0 | Sb06g017240 | mitogen-activated kinase kinase kinase alpha, putative, expressed |
| AK100347 | LOC_Os04g37670 | 1.87 | 0 | Sb06g018270 | expressed protein |
| AK100685 | LOC_Os02g57270 | 1.76 | 0 | Sb06g019970 | Myb-like DNA-binding domain containing protein, expressed |
| AK105549 | LOC_Os04g40540 | 1.51 | 0 | Sb06g020300 | Protein-L-isoaspartate O-methyltransferase, putative, expressed |
| AK058766 | LOC_Os08g13890 | 1.73 | 0 | Sb06g020650 | Exonuclease, putative, expressed |
| AK103012 | LOC_Os04g45160 | 1.73 | 0 | Sb06g023680 | expressed protein |
| AK071118 | LOC_Os04g47780 | 1.82 | 0 | Sb06g025470 | Amino acid transport protein, putative, expressed |
| AK101209 | LOC_Os04g49450 | 3.32 | 0 | Sb06g026500 | myb-like DNA-binding domain, SHAQKYF class family protein, expressed |
| AK072902 | LOC_Os04g50110 | 2.35 | 0 | Sb06g026990 | RNA recognition motif family protein, expressed |
| AK060169 | LOC_Os04g51786 | 1.54 | 0 | Sb06g027860 | Unknown |
| AK100900 | LOC_Os04g13170 | 1.51 | 0 | Sb06g030200 | F-box domain containing protein, expressed |
| AK072534 | LOC_Os04g56450 | 2.12 | 0 | Sb06g031490 | Protein phosphatase 2C containing protein, expressed |
| AK067526 | LOC_Os08g01220 | 2.46 | 0 | Sb07g000370 | Harpin-induced protein 1 containing protein, expressed |
| AK059891 | LOC_Os08g02420 | 1.55 | 0 | Sb07g001680 | EF hand family protein, expressed |
| AK061094 | LOC_Os08g04790 | 1.63 | 0 | Sb07g003140 | glycosyltransferase family 14 protein, putative, expressed |
| AK106834 | LOC_Os01g29409 | 1.89 | 0 | Sb07g004640 | Unknown |
| AK101717 | LOC_Os08g08060 | 1.75 | 0 | Sb07g004940 | Pep3/Vps18/deep orange family protein, expressed |
| AK061580 | LOC_Os08g29500 | 1.5 | 0 | Sb07g019330 | repressor protein, putative, expressed |
| AK062031 | LOC_Os08g31410 | 1.79 | 0 | Sb07g020050 | Sulfate transporter 1.2, putative, expressed |
| AK103087 | LOC_Os08g32600 | 1.51 | 0 | Sb07g020580 | Protein kinase domain containing protein, expressed |
| AK059480 | LOC_Os08g34070 | 1.51 | 0 | Sb07g021520 | DCN1-like protein 4, putative, expressed |
| AK064682 | LOC_Os11g04440 | 1.53 | 0.03 | Sb08g001270 | Mitochondrial glycoprotein, expressed |
| AK105366 | LOC_Os12g31350 | 1.5 | 0 | Sb08g015330 | SSXT protein containing protein, expressed |
| AK067120 | LOC_Os12g42060 | 2.38 | 0 | Sb08g021525 | wall-associated kinase 3, putative, expressed |
| AK102252 | LOC_Os01g18290 | 1.95 | 0.02 | Sb09g003090 | Helix-loop-helix DNA-binding domain containing protein, expressed |
| AK067112 | LOC_Os05g05200 | 1.53 | 0 | Sb09g003400 | MBOAT family protein, expressed |
| AK073497 | LOC_Os05g06110 | 2.06 | 0 | Sb09g004120 | Villin-1, putative, expressed |
| AK108453 | LOC_Os05g22614 | 1.69 | 0 | Sb09g010000 | Unknown |
| AK066003 | LOC_Os05g32970 | 3.14 | 0 | Sb09g019550 | expressed protein |
| AK102648 | LOC_Os05g45310 | 2.83 | 0 | Sb09g026380 | ER lumen protein retaining receptor, putative, expressed |
| AK100910 | LOC_Os05g50380 | 2.12 | 0 | Sb09g029610 | Glucose-1-phosphate adenylyltransferase large subunit 1 |
| AK068577 | LOC_Os11g19250 | 1.52 | 0.01 | Sb09g030040 | LOC495115 protein, putative, expressed |
| AK103630 | LOC_Os06g05250 | 2.26 | 0 | Sb10g003070 | GTP-binding protein lepA, putative, expressed |
| AK058798 | LOC_Os06g05880 | 2.22 | 0 | Sb10g003670 | Profilin-2, putative, expressed |
| AK069464 | LOC_Os06g06870 | 2.97 | 0 | Sb10g004465 | MIZ zinc finger family protein, expressed |
| AK061806 | LOC_Os06g12220 | 2.2 | 0 | Sb10g008020 | HVA22-like protein a, putative, expressed |
| AK071489 | LOC_Os06g51460 | 3.18 | 0 | Sb10g020390 | ABC transporter family protein, putative, expressed |
| AK103787 | LOC_Os06g41390 | 1.93 | 0 | Sb10g024340 | Zinc finger C-x8-C-x5-C-x3-H type family protein, expressed |
| AK072728 | LOC_Os06g50960 | 1.55 | 0 | Sb10g030575 | Pollen allergen family protein, expressed |
| AK067445 | LOC_Os11g28410 | 2.24 | 0 | Unknown | retrotransposon protein, putative, unclassified |
| AK106439 | LOC_Os08g17500 | 2.45 | 0 | Unknown | NmrA-like family protein, expressed |
| AK111879 | LOC_Os02g11930 | 2.84 | 0 | Unknown | Leucine Rich Repeat family protein, expressed |
| AK103242 | LOC_Os04g12980 | 2.41 | 0 | Unknown | Indole-3-acetate beta-glucosyltransferase, putative, expressed |
| AK101304 | Os11g0173600 | 1.78 | 0 | Unknown | Unknown |
| AK105319 | Os04g0462700 | 4.6 | 0 | Unknown | Unknown |
| AK100314 | LOC_Os01g09450 | 2.02 | 0 | Unknown | AUX/IAA family protein, expressed |
| AK062801 | LOC_Os11g04920 | 1.92 | 0 | Unknown | expressed protein |
| AK102113 | LOC_Os10g07400 | 2.34 | 0 | Unknown | NB-ARC domain containing protein, expressed |
| AK110824 | LOC_Os12g17320 | 3.1 | 0 | Unknown | Prenyl transferase, putative, expressed |
| AK107510 | Os05g0141100 | 1.55 | 0 | Unknown | THH1. |
| AK107338 | Unknown | 2.54 | 0 | Unknown | Unknown |
| AK070643 | LOC_Os07g10920 | 2.42 | 0 | Unknown | expressed protein |
| AK106309 | LOC_Os06g16240 | 1.68 | 0 | Unknown | expressed protein |
| AK070763 | LOC_Os06g20200 | 1.73 | 0 | Unknown | serine hydrolase, putative, expressed |
| AK064207 | Os10g0560600 | 2.93 | 0 | Unknown | Unknown |
| AK065686 | LOC_Os09g28230 | 2.21 | 0 | Unknown | 2-Hydroxyisoflavanone dehydratase, putative, expressed |
| AK106334 | Unknown | 1.67 | 0 | Unknown | Unknown |
| AK064687 | LOC_Os05g10550 | 1.59 | 0 | Unknown | hypothetical protein |
| AK065838 | LOC_Os12g06380 | 2.87 | 0 | Unknown | transposon protein, putative, unclassified, expressed |
| AK072730 | LOC_Os01g28690 | 3.66 | 0 | Unknown | expressed protein |
| AK065424 | Os06g0667400 | 2.39 | 0 | Unknown | Unknown |
| AK070057 | LOC_Os01g35920 | 1.58 | 0 | Unknown | Unknown |
| AK105584 | LOC_Os08g27250 | 4.46 | 0 | Unknown | expressed protein |
| AK064677 | LOC_Os04g02570 | 1.95 | 0 | Unknown | hypothetical protein |
| AK072355 | LOC_Os11g11810 | 1.58 | 0 | Unknown | NB-ARC domain containing protein, expressed |
| AK100913 | LOC_Os02g31922 | 1.53 | 0 | Unknown | Unknown |
| AK064547 | Os04g0353200 | 1.61 | 0 | Unknown | Conserved hypothetical protein. |
| AK108509 | Os03g0718400 | 1.51 | 0 | Unknown | Hypothetical protein. |
| AK105195 | Os01g0278100 | 1.69 | 0 | Unknown | Conserved hypothetical protein. |
| AK101696 | LOC_Os05g16824 | 1.9 | 0 | Unknown | Unknown |
| AK107051 | LOC_Os09g31000 | 2.06 | 0 | Unknown | EF hand family protein, expressed |
| AK061534 | LOC_Os01g03810 | 1.56 | 0 | Unknown | expressed protein |
| AK071496 | LOC_Os06g28630 | 2.46 | 0 | Unknown | expressed protein |
| AK062968 | Os05g0581000 | 2.2 | 0 | Unknown | Unknown |
| AK099550 | LOC_Os04g28480 | 1.99 | 0 | Unknown | expressed protein |
| AK073973 | LOC_Os05g29010 | 2.24 | 0 | Unknown | DNA primase small subunit, putative, expressed |
| AK066114 | LOC_Os01g62750 | 1.63 | 0 | Unknown | expressed protein |
| AK100353 | Os05g0374000 | 2.16 | 0 | Unknown | Thioredoxin-related domain containing protein. |
| AK061184 | Unknown | 1.9 | 0 | Unknown | Unknown |
| AK072173 | Os11g0211400 | 1.57 | 0 | Unknown | Ankyrin repeat containing protein. |
| AK101202 | Os04g0632400 | 3.45 | 0 | Unknown | Conserved hypothetical protein. |
| AK110390 | Unknown | 1.86 | 0.01 | Unknown | Unknown |
| AK110527 | Unknown | 1.8 | 0.01 | Unknown | Unknown |
| AK071936 | Os09g0116900 | 2.23 | 0.01 | Unknown | Hypothetical protein. |
| AK109805 | Os05g0324400 | 1.99 | 0.01 | Unknown | Hypothetical protein. |
| AK107160 | LOC_Os03g61310 | 2.34 | 0.02 | Unknown | D-mannose binding lectin family protein, expressed |
| AK068683 | LOC_Os11g07470 | 2.65 | 0.02 | Unknown | expressed protein |
| AK070416 | LOC_Os03g01870 | 1.87 | 0.02 | Unknown | NAC-domain containing protein 21/22, putative, expressed |
| AK106994 | LOC_Os02g29590 | 1.66 | 0.07 | Unknown | expressed protein |
| AK100384 | Os06g0188500 | 1.62 | 0.07 | Unknown | Unknown |
| AK100001 | Os12g0562800 | 1.53 | 0.07 | Unknown | Unknown |
| AK060454 | Unknown | 1.61 | 0.14 | Unknown | Unknown |

^a^ Fold Change represents the ratio of Avg_SI vs. MAX (Avg_ST, Avg_RI, Avg_RT, and Avg_YL), and q-value (%) ≤5 %, while Avg_x represents the average ratio of the three biological replicates while RT for Rhizome tips/control, ST for Shoot tips/control, RI for Rhizome internodes/control, SI for Stem internodes/control and YL for Young leaves/control.
