# Supplementary material for: Tissue-Specific Transcriptomic Profiling of Sorghum propinquum using a Rice Genome Array
Source: PLoS One. 2013 Mar 25;8(3):e60202. doi: 10.1371/journal.pone.0060202 (PMC3607598; doi:10.1371/journal.pone.0060202)
Supplement: Table S7 — The list of genes enriched specifically in rhizome internodes relative to other tissues. (DOC) [file pone.0060202.s008.doc]

**Table S7.** The list of genes enriched specifically in rhizome internodes relative to other tissues.

| **Name** | **Oryza GI** | **Fold Change^a^** | **q-value(%)** | **Best Sorghum BLAST hit** | **Function Annotation** |
| --- | --- | --- | --- | --- | --- |
| AK101509 | LOC_Os05g02210 | 1.65 | 0 | Sb01g000980 | pentatricopeptide |
| AK060496 | LOC_Os03g63400 | 1.51 | 0 | Sb01g001100 | NAC domain containing protein |
| AK069826 | LOC_Os03g63270 | 1.8 | 0.01 | Sb01g001260 | regulatory protein |
| AK101786 | LOC_Os03g38010 | 1.68 | 0 | Sb01g001320 | hypothetical protein |
| AK066994 | LOC_Os03g62240 | 1.8 | 0.51 | Sb01g001940 | expressed protein |
| AK068540 | LOC_Os03g54780 | 2.14 | 0 | Sb01g007720 | Serine/threonine kinase |
| AK102204 | LOC_Os03g37640 | 2.23 | 0 | Sb01g016100 | MATE efflux family protein |
| AK100448 | LOC_Os04g21610 | 1.55 | 0 | Sb01g022170 | hypothetical protein |
| AK107029 | LOC_Os10g25140 | 1.69 | 0 | Sb01g023740 | Alanine aminotransferase 2 |
| AK108770 | LOC_Os10g06680 | 1.55 | 0 | Sb01g025850 | expressed protein |
| AK101034 | LOC_Os07g41330 | 1.71 | 0.01 | Sb01g032490 | Mitochondrial import inner membrane translocase subunit Tim17 B |
| AK059026 | LOC_Os03g22880 | 1.53 | 0 | Sb01g035290 | Nucleolar protein Nop56 |
| AK059469 | LOC_Os03g21080 | 3.08 | 0 | Sb01g036550 | Guanine nucleotide exchange factor |
| AK100114 | LOC_Os03g15240 | 1.91 | 0 | Sb01g040610 | expressed protein |
| AK073446 | LOC_Os03g10210 | 1.88 | 0 | Sb01g043910 | Homeobox domain containing protein |
| AK103687 | LOC_Os03g09860 | 4.55 | 0 | Sb01g044190 | acetyltransferase GNAT family protein |
| AK105352 | LOC_Os03g08880 | 1.5 | 0.07 | Sb01g044690 | expressed protein |
| AK068020 | LOC_Os07g01130 | 1.66 | 0.27 | Sb02g000310 | expressed protein |
| AK098912 | LOC_Os07g05570 | 2.2 | 0 | Sb02g003010 | ERD4 protein |
| AK101136 | LOC_Os05g01230 | 1.6 | 0.01 | Sb02g008650 | zinc finger family protein |
| AK099867 | LOC_Os01g32280 | 1.69 | 0 | Sb02g035910 | hypothetical protein |
| AK063406 | LOC_Os07g37920 | 3.49 | 0 | Sb02g036620 | NAM |
| AK101099 | LOC_Os07g41250 | 1.64 | 0 | Sb02g038680 | POT family protein |
| AK111132 | LOC_Os07g44310 | 1.99 | 0 | Sb02g040590 | DnaJ domain containing protein |
| AK060668 | LOC_Os06g19640 | 3.14 | 0 | Sb02g041260 | expressed protein |
| AK065412 | LOC_Os07g47490 | 2 | 0 | Sb02g042520 | Indole-3-acetic acid-amido synthetase GH3.9 |
| AK107065 | LOC_Os07g48830 | 1.54 | 0.01 | Sb02g043450 | galactinol synthase 3 |
| AK066024 | LOC_Os01g12880 | 1.62 | 0 | Sb03g000910 | VHS domain containing protein |
| AK108512 | LOC_Os01g12740 | 2.12 | 0 | Sb03g001040 | Cytochrome P450 family protein |
| AK103122 | LOC_Os12g06640 | 1.62 | 0 | Sb03g013050 | expressed protein |
| AK071695 | LOC_Os01g40630 | 2.22 | 0 | Sb03g026430 | Lysine Decarboxylase |
| AK061091 | LOC_Os01g51210 | 1.65 | 0 | Sb03g032570 | lysine decarboxylase |
| AK108715 | LOC_Os05g45350 | 1.7 | 0 | Sb03g033630 | DnaJ domain containing protein |
| AK062985 | LOC_Os05g45220 | 1.51 | 0 | Sb03g033790 | 50S ribosomal protein L20 |
| AK067886 | LOC_Os01g70020 | 1.74 | 0 | Sb03g044330 | expressed protein |
| AK101693 | LOC_Os05g02060 | 1.87 | 0 | Sb03g047420 | amino acid selective channel protein |
| AK106802 | LOC_Os02g03750 | 1.71 | 0 | Sb04g002530 | Polygalacturonase family protein |
| AK065460 | LOC_Os02g27340 | 2.19 | 0 | Sb04g018500 | Riboflavin biosynthesis protein ribD |
| AK063644 | LOC_Os02g33560 | 1.56 | 0 | Sb04g022020 | expressed protein |
| AK108806 | LOC_Os02g48810 | 5.66 | 0 | Sb04g029710 | PHD-finger family protein |
| AK067007 | LOC_Os02g47470 | 2.11 | 0 | Sb04g030660 | Cytochrome P450 family protein |
| AK068163 | LOC_Os02g57290 | 1.89 | 0 | Sb04g037300 | Cytochrome P450 family protein |
| AK102697 | LOC_Os11g04740 | 3.53 | 0 | Sb05g002676 | L-galactono-1 |
| AK066452 | LOC_Os11g25780 | 1.58 | 0.01 | Sb05g012960 | PB1 domain containing protein |
| AK108542 | LOC_Os11g31940 | 1.77 | 0 | Sb05g018910 | GDSL-like Lipase/Acylhydrolase family protein |
| AK099612 | LOC_Os11g43510 | 1.53 | 0 | Sb05g025890 | Lipase family protein |
| AK101106 | LOC_Os04g31030 | 1.82 | 0 | Sb06g012960 | Nitrate-induced NOI protein |
| AK107473 | LOC_Os04g32670 | 1.99 | 0 | Sb06g014730 | exostosin family protein |
| AK100669 | LOC_Os04g32920 | 3.12 | 0 | Sb06g014930 | Potassium transporter 1 |
| AK110053 | LOC_Os04g39680 | 2.44 | 0 | Sb06g019790 | C2 domain containing protein |
| AK100299 | LOC_Os04g40910 | 1.79 | 0 | Sb06g020660 | F-box domain containing protein |
| AK062810 | LOC_Os04g41900 | 1.65 | 0 | Sb06g021400 | expressed protein |
| AK099234 | LOC_Os04g53230 | 1.6 | 0 | Sb06g029020 | Aminomethyltransferase |
| AK109716 | LOC_Os04g54400 | 1.56 | 0.07 | Sb06g030040 | transposon protein |
| AK105170 | LOC_Os04g55080 | 1.96 | 0 | Sb06g030420 | Cyclic nucleotide-gated ion channel 7 |
| AK103715 | LOC_Os06g43600 | 1.7 | 0 | Sb06g030490 | Protease inhibitor/seed storage/LTP family protein |
| AK070857 | LOC_Os04g57600 | 1.62 | 0 | Sb06g032480 | Zinc finger C-x8-C-x5-C-x3-H type family protein |
| AK070877 | LOC_Os08g08070 | 1.51 | 0 | Sb07g004950 | Glucose transporter |
| AK105596 | LOC_Os08g10080 | 1.56 | 0.02 | Sb07g005610 | NAC-domain containing protein 21/22 |
| AK105353 | LOC_Os09g08670 | 1.72 | 0 | Sb07g013790 | expressed protein |
| AK067128 | LOC_Os04g23890 | 2.72 | 0 | Sb07g014860 | Nonphototropic hypocotyl protein 1 |
| AK061260 | LOC_Os08g30820 | 1.7 | 0 | Sb07g019840 | pre-mrna cleavage factor |
| AK067282 | LOC_Os08g34060 | 2.24 | 0 | Sb07g021510 | expressed protein |
| AK059773 | LOC_Os08g36630 | 1.53 | 0.02 | Sb07g022860 | Eukaryotic-type carbonic anhydrase family protein |
| AK111028 | LOC_Os08g41670 | 4.43 | 0 | Sb07g026430 | Uncharacterized protein family UPF0016 containing protein |
| AK111069 | LOC_Os08g37520 | 1.53 | 0 | Sb07g029130 | pentatricopeptide |
| AK072890 | LOC_Os08g37570 | 2.37 | 0 | Sb07g029140 | U-box domain containing protein |
| AK073696 | LOC_Os12g19350 | 2.17 | 0 | Sb08g010820 | expressed protein |
| AK070653 | LOC_Os05g03550 | 1.86 | 0 | Sb09g002290 | Myb-like DNA-binding domain containing protein |
| AK069092 | LOC_Os05g19500 | 2.91 | 0 | Sb09g008200 | cation/hydrogen exchanger |
| AK102727 | LOC_Os05g27950 | 1.52 | 0 | Sb09g016210 | cp protein |
| AK068336 | LOC_Os05g45180 | 5.44 | 0 | Sb09g026270 | UDP-glucoronosyl and UDP-glucosyl transferase family protein |
| AK065770 | LOC_Os05g45300 | 1.78 | 0 | Sb09g026370 | enoyl-CoA hydratase/isomerase family protein |
| AK103500 | LOC_Os06g02600 | 1.59 | 0.84 | Sb10g001390 | DAG protein |
| AK108564 | LOC_Os06g44220 | 2.14 | 0 | Sb10g025810 | Uncharacterized protein family protein |
| AK101523 | LOC_Os12g25200 | 1.55 | 0.03 | Sb10g026090 | Chloride channel protein CLC-a |
| AK066454 | LOC_Os02g04730 | 12.65 | 0 | unknown | Cycloartenol synthase |
| AK107519 | LOC_Os11g26770 | 12.04 | 0 | unknown | transposon protein |
| AK073869 | Os11g0579300 | 4.25 | 0 | unknown | hypothetical protein |
| AK109701 | LOC_Os01g14514 | 2.16 | 0 | unknown | hypothetical protein |
| AK100638 | LOC_Os10g35090 | 1.81 | 0 | unknown | hypothetical protein |
| AK059130 | LOC_Os08g03020 | 1.53 | 0 | unknown | Legume lectins beta domain containing protein |
| AK108524 | unknown | 9.79 | 0 | unknown | unknown |
| AK058767 | LOC_Os01g05780 | 1.51 | 0 | unknown | expressed protein |
| AK107086 | LOC_Os03g10334 | 2.38 | 0 | unknown | hypothetical protein |
| AK106378 | LOC_Os09g03190 | 5.19 | 0 | unknown | expressed protein |
| AK068661 | Os01g0647200 | 1.66 | 0 | unknown | hypothetical protein |
| AK102518 | LOC_Os11g47830 | 1.52 | 0 | unknown | RNA recognition motif family protein |
| AK109580 | LOC_Os02g28470 | 1.52 | 0 | unknown | Transferase family protein |
| AK110892 | LOC_Os04g12690 | 1.67 | 0 | unknown | Indole-3-acetate beta-glucosyltransferase |
| AK111391 | LOC_Os04g47670 | 1.61 | 0 | unknown | expressed protein |
| AK108713 | LOC_Os11g43980 | 1.56 | 0 | unknown | Peroxidase 1 precursor |
| AK109196 | Os04g0617200 | 4.62 | 0 | unknown | TPR-like domain containing protein. |
| AK070013 | LOC_Os09g15389 | 3.09 | 0 | unknown | hypothetical protein |
| AK058540 | unknown | 1.54 | 0 | unknown | unknown |
| AK109002 | unknown | 1.54 | 0 | unknown | unknown |
| AK064901 | LOC_Os08g29020 | 4.52 | 0 | unknown | wall-associated kinase 2 |
| AK106375 | LOC_Os12g36610 | 1.51 | 0 | unknown | Patatin-like phospholipase family protein |
| AK108671 | LOC_Os08g03470 | 2.51 | 0 | unknown | BTB/POZ domain containing protein |
| AK063655 | LOC_Os09g21290 | 1.52 | 0 | unknown | expressed protein |
| AK106640 | LOC_Os02g31074 | 2.07 | 0 | unknown | hypothetical protein |
| AK062177 | unknown | 2.93 | 0 | unknown | unknown |
| AK067149 | LOC_Os03g06379 | 1.78 | 0 | unknown | hypothetical protein |
| AK110849 | LOC_Os04g28234 | 1.62 | 0 | unknown | hypothetical protein |
| AK099376 | LOC_Os06g30440 | 1.71 | 0 | unknown | GH3 auxin-responsive promoter family protein |
| AK061464 | Os03g0177500 | 1.82 | 0 | unknown | EF-1 alpha. |
| AK111425 | Os09g0519000 | 1.97 | 0 | unknown | Conserved hypothetical protein. |
| AK058864 | LOC_Os11g37040 | 1.76 | 0 | unknown | expressed protein |
| AK060308 | LOC_Os08g35319 | 1.52 | 0 | unknown | hypothetical protein |
| AK102047 | LOC_Os02g35720 | 1.8 | 0 | unknown | Microsomal signal peptidase 25 kDa subunit family protein |
| AK108068 | LOC_Os02g25680 | 1.66 | 0.02 | unknown | expressed protein |
| AK106045 | Os06g0229900 | 1.51 | 0.07 | unknown | Conserved hypothetical protein. |
| AK071064 | LOC_Os08g01324 | 1.74 | 0.14 | unknown | hypothetical protein |
| AK065129 | LOC_Os11g10070 | 1.52 | 0.51 | unknown | expressed protein |

^a^ Fold Change represents the ratio of Avg_RI vs. MAX (Avg_ST, Avg_RT, Avg_SI, and Avg_YL), and q-value (%) ≤5 %, while Avg_x represents the average ratio of the three biological replicates while RT for Rhizome tips/control, ST for Shoot tips/control, RI for Rhizome internodes/control, SI for Stem internodes/control and YL for Young leaves/control.
