# Supplementary material for: Tissue-Specific Transcriptomic Profiling of Sorghum propinquum using a Rice Genome Array
Source: PLoS One. 2013 Mar 25;8(3):e60202. doi: 10.1371/journal.pone.0060202 (PMC3607598; doi:10.1371/journal.pone.0060202)
Supplement: Table S8 — Rhizome-enriched genes on the rhizome-related QTLs regions identified in sorghum and rice. (DOC) [file pone.0060202.s009.doc]

**Table S8.** Rhizome-enriched genes on the rhizome-related QTLs regions identified in sorghum and rice.

| **Trait/LG:Interval** | **TIGR** | **UG/AG^c^** | **Best Sorghum homologous** | **Corresponding QTL intervals in the rice genome** | **Function annotation** |
| --- | --- | --- | --- | --- | --- |
| LAR^a^ |  |  |  |  |  |
| pSB643b-pSB041 | LOC_Os03g38010 | RI | Sb01g001320 |  | nuf2 family protein, expressed |
|  | LOC_Os03g62240 | RI | Sb01g001940 |  | expressed protein |
|  | LOC_Os03g63270 | RI | Sb01g001260 |  | regulatory protein, putative, expressed |
|  | LOC_Os03g63400 | RI | Sb01g001100 |  | transcription factor BTF3, putative, expressed |
|  | LOC_Os05g02210 | RI | Sb01g000980 |  | pentatricopeptide, putative |
|  | LOC_Os02g28170 | 1.67 | Sb01g000340 |  | Transferase family protein. |
|  | LOC_Os03g61060 | 1.64 | Sb01g002800 |  | Tyrosine protein kinase domain containing protein. |
|  | LOC_Os03g62580 | 2.30 | Sb01g001730 |  | Vacuolar sorting protein 9 domain |
| LAR;LSR |  |  |  |  |  |
| pSB195-SHO68 | LOC_Os03g37640 | RI | Sb01g016100 |  | MATE efflux family protein, putative, expressed |
|  | LOC_Os04g21610 | RI | Sb01g022170 |  | expressed protein |
|  | LOC_Os10g06680 | RI | Sb01g025850 |  | expressed protein |
|  | LOC_Os10g25140 | RI | Sb01g023740 |  | aminotransferase, classes I and II, domain |
|  | LOC_Os10g42670 | RI | Sb01g027960 |  | glycosyl hydrolases family 16, putative, expressed |
|  | LOC_Os06g09870 | 7.39 |  |  | Glycine-rich cell wall structural protein 2 precursor |
|  | LOC_Os10g05730 | 1.76 | Sb01g026210 |  | ARM repeat fold domain containing protein |
|  | LOC_Os10g09240 | 1.67 | Sb01g015750 |  | Conserved hypothetical protein |
|  | LOC_Os10g20250 | 1.55 | Sb01g024850 |  | Protein of unknown function DUF588 family protein |
|  | LOC_Os10g27170 | 1.67 | Sb01g022210 |  | IQ calmodulin-binding region domain containing |
|  | LOC_Os10g32900 | 1.60 | Sb01g019780 | RM271–RM269 | CONSTANS-like protein CO9 (Fragment). |
| pSB300a-pSB088 | LOC_Os03g07480 | RT | Sb01g045720 |  | sucrose transporter, putativ, expressed |
|  | LOC_Os03g08880 | RI | Sb01g044690 |  | purine permease, putative, expressed |
|  | LOC_Os03g09860 | RI | Sb01g044190 |  | acetyltransferase, GNAT family, putative, expressed |
|  | LOC_Os03g10210 | RI | Sb01g043910 |  | homeobox domain containing protein, expressed |
|  | LOC_Os03g15240 | RI | Sb01g040610 |  | hypothetical protein |
|  | LOC_Os03g21080 | RI | Sb01g036556 |  | guanine nucleotide exchange factor |
|  | LOC_Os03g09080 | 2.08 | Sb01g044530 |  | Ubiquitin-specific protease 4 (UBP4) |
|  | LOC_Os03g14120 | 2.00 | Sb01g041240 |  | Dihydrodipicolinate reductase family protein |
|  | LOC_Os06g28950 | 1.63 | Sb01g038960 |  | Hypothetical protein. |
|  | LOC_Os09g39570 | 1.53 | Sb01g045866 |  |  |
| pSB102-pSB158 | LOC_Os03g54780 | RI | Sb01g007720 |  | STE_PAK_Ste20_KHSh_GCKh_HPKh.1 |
|  | LOC_Os08g03020 | RI |  |  |  |
| LSR |  |  |  |  |  |
| pSB188-pSB428 | LOC_Os04g31030 | RI | Sb06g012960 |  | nitrate-induced NOI protein |
| pSB445-pSB069 | LOC_Os05g10840 | RT | Sb09g006130 |  | calmodulin-binding family protein |
|  | LOC_Os05g39840 | RT | Sb09g023350 | RM161–RM274 | expressed protein |
|  | LOC_Os05g19500 | RI | Sb09g008200 |  | ATCHX, putative, expressed |
|  | LOC_Os05g27950 | RI | Sb09g016210 |  | DUF538 domain containing protein |
|  | LOC_Os05g31720 | 2.15 | Sb09g018900 |  | Arf GTPase activating protein family protein |
|  | LOC_Os05g38420 | 1.87 | Sb09g022510 | RM161–RM274 | Laccase (EC 1.10.3.2) |
|  | LOC_Os05g39210 | 1.95 |  | RM161–RM274 | Galactose oxidase, central domain containing |
| pSB077-Psb103 | LOC_Os07g33660 | RT | Sb02g034900 |  | expressed protein |
|  | LOC_Os09g38040 | 1.82 | Sb02g032250 |  | NUDIX hydrolase domain containing protein |
| LSR;Regrowth |  |  |  |  |  |
| pSB038-pSB512 | LOC_Os02g46750 | RT | Sb04g031140 |  | expressed protein |
|  | LOC_Os02g27340 | RI | Sb04g018500 |  | riboflavin biosynthesis protein ribD |
|  | LOC_Os02g33560 | RI | Sb04g022020 | RM341–RM327 | expressed protein |
|  | LOC_Os02g47470 | RI | Sb04g030660 |  | cytochrome P450, putative |
|  | LOC_Os02g48810 | RI | Sb04g029710 |  | PHD-finger family protein |
|  | LOC_OS11G26770 | RI |  |  |  |
|  | LOC_Os02g17970 | 1.54 | Sb04g011020 | RM71–RM300 | Protein phosphatase 2C family protein |
|  | LOC_Os02g30410 | 2.14 | Sb04g020600 |  | Protein of unknown function DUF493 family protein |
|  | LOC_Os02g31290 | 2.20 | Sb04g021130 |  |  |
|  | LOC_Os02g32750 | 2.17 | Sb04g021570 | RM341–RM327 | Xyloglucan 6-xylosyltransferase (EC 2.4.2.39) |
|  | LOC_Os02g35900 | 1.59 | Sb04g023500 |  | Thioredoxin-related domain containing protein |
|  | LOC_Os02g39070 | 2.07 | Sb04g025220 |  | Got1-like protein family protein |
|  | LOC_Os02g41650 | 1.57 | Sb04g026520 |  | Phenylalanine ammonia-lyase |
|  | LOC_Os02g51160 | 2.03 | Sb04g027980 |  | Hypothetical protein |
| pSB106-pSB430a | LOC_Os03g28960 | RT | Sb10g006995 |  | DNA-directed RNA polymerase III 130 kDa |
|  | LOC_Os05g41870 | RT | Sb10g009500 | RM161–RM274 | glycine-rich cell wall protein |
|  | LOC_Os06g02600 | RI | Sb10g001390 |  | DAG protein, chloroplast precursor |
|  | LOC_Os06g12450 | RI | Sb10g008200 |  | soluble starch synthase 2-3, chloroplast precursor |
|  | LOC_Os06g19640 | RI | Sb10g011040 |  | 39S ribosomal protein L46, mitochondrial precursor |
|  | LOC_Os06g03790 | 1.52 | Sb10g001740 |  | Mitochondrial 39-S ribosomal L47 family protein |
|  | LOC_Os06g05190 | 1.50 | Sb10g003040 |  | BRCT domain containing protein |
|  | LOC_Os06g07630 | 2.30 | Sb10g005010 |  | 26S protease regulatory subunit 6A |
|  | LOC_Os06g09370 | 1.59 | Sb10g006250 |  | Helix-loop-helix protein homolog |
|  | LOC_Os06g11240 | 1.94 | Sb10g007330 |  | Oxo-phytodienoic acid reductase |
|  | LOC_Os06g12310 | 1.51 | Sb10g008090 |  | Major intrinsic protein family protein |
|  | LOC_Os06g13220 | 2.20 | Sb10g008690 |  | Hypothetical protein |
| Regrowth |  |  |  |  |  |
| pSB614-pSB613 | LOC_Os01g40630 | RI | Sb03g026430 |  | uncharacterized protein PA4923 |
|  | LOC_Os01g39830 | 1.67 | Sb03g025990 |  | Beta-galactosidase precursor |
|  | LOC_Os01g42720 | 1.61 | Sb03g027760 |  | Conserved hypothetical protein |
|  | LOC_Os05g36070 | 1.76 | Sb03g026896 | RM161–RM274 | Peptidase A22B |
| pSB067-pSB784 | LOC_Os08g08070 | RI | Sb07g004950 |  | transporter family protein |
|  | LOC_Os08g10080 | RI | Sb07g005610 |  | no apical meristem protein |
|  | LOC_Os08g08830 | 1.97 | Sb07g005190 |  | Regulation of nuclear pre-mRNA protein |
|  |  |  |  |  |  |
| Rhz3^b^ | LOC_Os04g39680 | RI | Sb06g019790 | RM119–RM273 | anthranilate phosphoribosyltransferase |
|  | LOC_Os04g35540 | 1.51 | Sb06g017100 |  | Amino acid/polyamine transporter I family protein |
|  | LOC_Os04g39190 | 1.84 | Sb06g019360 |  | Cell division protein FtsH-like protein |
|  | LOC_Os04g39410 | 2.12 | Sb06g019570 |  | TPR-like domain containing protein |
|  | LOC_Os04g40100 | 1.88 | Sb06g020070 |  | NPH3 domain containing protein |
| QRn5 | LOC_Os05g45180 | RI | Sb09g026280 | RM161–RM274 | anthocyanidin 5,3-O-glucosyltransferase |
|  | LOC_Os05g45220 | RI | Sb09g026330 |  | 50S ribosomal protein L20 |
|  | LOC_Os05g45300 | RI | Sb09g026370 |  | enoyl-CoA hydratase/isomerase family protein |
|  | LOC_Os05g45350 | RI | Sb09g026410 |  | dnaJ domain containing protein |
|  | LOC_Os05g35340 | 1.94 |  |  | Heat shock protein DnaJ family protein |
|  | LOC_Os05g37460 | 1.76 |  |  | AtPH1-like protein |
|  | LOC_Os05g41090 | 1.99 | Sb10g029700 |  | Calcium/calmodulin-dependent protein kinase |
|  | LOC_Os05g43970 | 1.65 |  |  | 28 kDa heat- and acid-stable phosphoprotein |
|  | LOC_Os05g46420 | 2.15 |  |  | Ipomoelin |
| QRn10 | LOC_Os10g32830 | 1.59 |  | RM271–RM269 | Conserved hypothetical protein |
| QRn3 | LOC_Os03g22400 | 2.10 | Sb01g035640 | RM282–RM5551 | SUMO protease |
| QRl1 | LOC_OS01G45914 | RI |  | RM306-RM237 |  |
|  | LOC_Os01g45370 | 1.85 | Sb03g029230 |  | Conserved hypothetical protein 48 family protein |
| QRl6 | LOC_Os06g44220 | RI | Sb10g025810 | RM30–RM7309 | OsRCI2-9 - low temperature and salt responsive |
|  | LOC_Os06g44210 | 1.75 | Sb10g025800 |  | Protein phosphatase type-2C |
| QRl7 | LOC_Os07g37920 | RI | Sb02g036620 | RM336–RM234 | No apical meristem (NAM) protein |
|  | LOC_Os07g41250 | RI | Sb02g038680 |  | peptide transporter PTR2 |
|  | LOC_Os07g41330 | RI | Sb02g035670 |  | translocase subunit Tim17 |
|  | LOC_Os07g38490 | 2.16 | Sb02g037060 |  | Inorganic pyrophosphatase family protein |
|  | LOC_Os07g39710 | 1.78 | Sb02g037780 |  | NADH-ubiquinone oxidoreductase 18 kDa subunit |

^a^ Sorghum rhizome QTLs identified in the interspecific F_2_ population of *S. propinquum* (Paterson et al., 1995). LAR: the log (n +1) of the number of

rhizomes producing above-ground shoots; LSR: the log(n+1) of subterranean rhizomatousness.

^b^ Rhizome QTLs identified in the F_2_ population between RD23 (*O. sativa*) and *O. longistaminata* (Hu et al., 2003).

^c^ The ratio of the expression level in underground tissues (UG) including root tips and rhizome internodes over that in the above ground tissues (AG)

including shoot tips, shoot internodes and young leaf. RT or RI indicates genes specifically expressed in RT or root internodes (RI). When a gene was

both in RT or RI and UG/AG, we took it for RT or RI enriched.
