# Supplementary material for: Tissue-Specific Transcriptomic Profiling of Sorghum propinquum using a Rice Genome Array
Source: PLoS One. 2013 Mar 25;8(3):e60202. doi: 10.1371/journal.pone.0060202 (PMC3607598; doi:10.1371/journal.pone.0060202)
Supplement: Table S9 — The list of down regulated genes in the comparison of the rhizome related DEGs. (DOC) [file pone.0060202.s010.doc]

**Table S9.** The list of down regulated genes in the comparison of the rhizome related DEGs.

| **Oryza ID** | **E^a^** | **F** | **A** | **B** | **C** | **D** | **Annotation** |
| --- | --- | --- | --- | --- | --- | --- | --- |
| LOC_Os01g13570 | 0.66 |  |  | 0.18 |  | 0.02 | Phosphoglycerate/bisphosphoglycerate mutase family protein. |
| LOC_Os10g31810 | 0.65 |  |  | 0.79 |  |  | Conserved hypothetical protein. |
| LOC_Os02g02740 | 0.64 |  |  | 0.85 |  |  | TPR-like domain containing protein. |
| LOC_Os01g67720 | 0.63 |  | 0.90 |  | 0.5 |  | ABC-1 domain containing protein. |
| LOC_Os01g53670 | 0.63 | 0.39 | 0.92 |  |  |  | Epsin, N-terminal domain containing protein. |
| LOC_Os10g40710 | 0.62 | 0.41 |  | 0.20 |  |  | Major pollen allergen Lol pI family protein. |
| LOC_Os01g59880 | 0.62 |  | 0.52 | 0.40 |  |  | Inositol-1, 4, 5-trisphosphate 5-phosphatase-like protein. |
| LOC_Os01g13090 | 0.61 |  |  | 0.75 |  |  | Conserved hypothetical protein. |
| LOC_Os08g40060 | 0.61 |  |  | 0.27 |  |  | Conserved hypothetical protein. |
| LOC_Os03g63860 | 0.61 |  |  | 0.67 |  |  | Conserved hypothetical protein. |
| LOC_Os02g26370 | 0.6 |  |  | 0.80 |  |  | Hypothetical protein. |
| LOC_Os11g26850 | 0.6 |  |  | 0.40 |  |  | Adenosylhomocysteinase (EC 3.3.1.1) |
| LOC_Os02g37830 | 0.6 |  |  | 0.88 |  |  | Protein kinase domain containing protein. |
| LOC_Os07g40940 | 0.59 |  |  | 0.20 |  |  | Conserved hypothetical protein. |
| LOC_Os06g46570 | 0.59 | 0.39 |  | 0.95 |  |  | Avr9 elicitor response-like protein. |
| LOC_Os02g22380 | 0.58 |  |  | 0.26 |  |  | Protein of unknown function DUF563 family protein. |
| LOC_Os07g46910 | 0.57 |  |  | 0.40 |  |  | Glucose/ribitol dehydrogenase family protein. |
| LOC_Os10g10170 | 0.57 | 0.34 | 0.94 | 0.40 |  | 0.1 | TPR-like domain containing protein. |
| LOC_Os08g34910 | 0.56 |  | 1.00 |  |  |  | Pectinmethylesterase precursor (EC 3.1.1.11) |
| LOC_Os12g37840 | 0.56 |  |  | 0.38 |  |  | HCO3-transporter domain containing protein. |
| LOC_Os01g01280 | 0.56 |  |  | 0.47 |  |  | Conserved hypothetical protein. |
| LOC_Os02g02910 | 0.55 |  |  | 0.34 |  | 0.18 | Conserved hypothetical protein. |
| LOC_Os01g07240 | 0.55 |  |  | 0.14 |  |  | Conserved hypothetical protein. |
| LOC_Os06g49780 | 0.55 |  |  | 0.46 |  |  | Conserved hypothetical protein. |
| LOC_Os09g28300 | 0.54 |  |  | 0.35 |  |  | Remorin, C-terminal region domain containing protein. |
| LOC_Os06g49010 | 0.53 |  |  | 0.32 |  |  | SBP-domain protein 5 (Fragment). |
| LOC_Os03g05800 | 0.53 |  |  |  |  | 0.17 | Conserved hypothetical protein. |
| LOC_Os02g57100 | 0.52 |  |  | 0.68 |  |  | HAD-superfamily hydrolase, subfamily IA, variant 1 protein. |
| LOC_Os05g03190 | 0.52 | 0.63 |  | 0.60 |  |  | IQ calmodulin-binding region domain containing protein. |
| LOC_Os02g05000 | 0.49 |  | 0.33 |  |  |  | Conserved hypothetical protein. |
| LOC_Os10g42670 | 0.47 |  |  | 0.38 |  |  | Glycoside hydrolase, family 16 domain containing protein. |
| LOC_Os12g39120 | 0.44 |  | 0.84 |  |  |  | Protein phosphatase 2C-like protein. |
| LOC_Os08g35740 | 0.43 | 0.63 |  | 0.95 |  |  | 12-oxophytodienoate reductase 3 (EC 1.3.1.42). |
| LOC_Os01g24680 | 0.32 | 0.29 |  | 0.69 |  |  | Glyoxysomal fatty acid beta-oxidation multifunctional MFP |
| LOC_Os04g40320 |  | 0.66 |  | 0.63 |  |  | Conserved hypothetical protein. |
| LOC_Os03g04080 |  | 0.66 | 0.75 |  |  |  | Hypothetical protein. |
| LOC_Os06g50960 |  | 0.65 |  | 0.24 |  |  | Pollen allergen/expansin, C-terminal domain containing protein. |
| LOC_Os02g46260 |  | 0.65 |  | 0.40 |  |  | Peptidase S10, serine carboxypeptidase family protein. |
| LOC_Os04g38680 |  | 0.64 |  | 0.50 |  |  | Amino acid/polyamine transporter II family protein. |
| LOC_Os04g54850 |  | 0.63 |  | 0.28 |  |  | Pectinesterase (EC 3.1.1.11) (Fragment). |
| LOC_Os05g23880 |  | 0.63 | 0.51 | 0.29 |  |  | Linoleate:oxygen oxidoreductase (Fragment). |
| LOC_Os08g34270 |  | 0.58 |  | 0.88 |  |  | Conserved hypothetical protein. |
| LOC_Os06g47000 |  | 0.57 |  | 0.50 | 0.47 |  | External rotenone-insensitive NADPH dehydrogenase. |
| LOC_Os01g20830 |  | 0.54 |  |  |  | 0.03 | Heavy metal transport/detoxification protein |
| LOC_Os12g42060 |  | 0.54 |  | 0.92 |  |  | Protein kinase domain containing protein. |
| LOC_Os01g06220 |  | 0.54 |  | 0.09 |  |  | Esterase/lipase/thioesterase domain containing protein. |
| LOC_Os01g46510 |  | 0.53 |  | 0.88 |  |  | WD40-like domain containing protein. |
| LOC_Os03g07970 |  | 0.52 |  | 0.35 |  |  | Conserved hypothetical protein. |
| LOC_Os04g52920 |  | 0.46 |  | 0.27 | 0.43 |  | Remorin, C-terminal region domain containing protein. |
| LOC_Os03g17170 |  | 0.45 |  |  |  | 0.18 | Zn-finger, RING domain containing protein. |
| LOC_Os07g43800 |  | 0.43 |  | 0.19 |  |  | Calcium-binding EF-hand domain containing protein. |
| LOC_Os09g38310 |  | 0.4 |  | 0.58 |  |  | Protein prenyltransferase domain containing protein. |
| LOC_Os04g43290 |  | 0.35 |  | 0.52 |  |  | Conserved hypothetical protein. |
| LOC_Os08g01220 |  | 0.34 |  | 0.29 |  |  | Harpin-induced 1 domain containing protein. |
| LOC_Os11g03390 |  | 0.23 | 0.57 | 0.23 |  | 0.05 | Forkhead-associated domain containing protein. |
| LOC_Os06g49360 |  | 0.17 |  | 0.70 |  |  | Disease resistance protein family protein. |
| LOC_Os01g11350 |  | 0.12 |  | 0.28 |  |  | Basic-leucine zipper (bZIP) transcription factor |
| LOC_Os07g14910 |  | 0.1 |  | 0.08 |  |  | Conserved hypothetical protein. |
| LOC_Os03g15920 |  | 0.08 |  |  |  | 0.16037 | Conserved hypothetical protein. |
| LOC_Os12g22600 |  | 0.07 |  | 0.19 | 0.38 |  | tRNA synthetase, class II (D, K and N) domain protein. |
| LOC_Os02g19970 |  | 0.04 |  | 0.43 |  |  | Nicotianamine aminotransferase A. |

**^a^** A~G represent the expression level of the 7 gene sets including DEGs of rhizome tip verse shoot tip in *O. longistaminata* from transcriptome sequencing data (A),

microarray analysis data (C) and results of the present study in *Sorghum propinquum* (E), DEGs of underground tissues (rhizome tip and rhizome internode) verse above

ground tissues (shoot tip, shoot internode and young leaf) in *O. longistaminata* from transcriptome analysis (B), microarray analysis (D) and results of the present study in

*Sorghum propinquum* (F), and candidate rhizome-enriched genes in *S. Halepense* (pSH) and *S. propinquum* (G).
